# Personality change in a trial of psilocybin therapy v. escitalopram treatment for depression

**DOI:** 10.1017/S0033291723001514

**Published:** 2023-06-02

**Authors:** Brandon Weiss, Induni Ginige, Lu Shannon, Bruna Giribaldi, Ashleigh Murphy-Beiner, Roberta Murphy, Michelle Baker-Jones, Jonny Martell, David J. Nutt, Robin L. Carhart-Harris, David Erritzoe

**Affiliations:** 1 Centre for Psychedelic Research, Division of Academic Psychiatry, Imperial College London, London, UK; 2 Psychedelics Division, Neuroscape, Department of Neurology, University of California, San Francisco, CA, USA

**Keywords:** Absorption, escitalopram, five-factor model, impulsivity, personality change, personality, psilocybin therapy

## Abstract

**Background.:**

Psilocybin Therapy (PT) is being increasingly studied as a psychiatric intervention. Personality relates to mental health and can be used to probe the nature of PT’s therapeutic action.

**Methods.:**

In a phase 2, double-blind, randomized, active comparator controlled trial involving patients with moderate-to-severe major depressive disorder, we compared psilocybin with escitalopram, over a core 6-week trial period. Five-Factor model personality domains, Big Five Aspect Scale Openness aspects, Absorption, and Impulsivity were measured at Baseline, Week 6, and Month 6 follow-up.

**Results.:**

PT was associated with decreases in neuroticism (*B* = −0.63), introversion (*B* = −0.38), disagreeableness (*B* = −0.47), impulsivity (*B* = −0.40), and increases in absorption (*B* = 0.32), conscientiousness (*B* = 0.30), and openness (*B* = 0.23) at week 6, with neuroticism (*B* = −0.47) and agreeableness (*B* = 0.41) remaining decreased at month 6. Escitalopram was associated with decreases in neuroticism (*B* = −0.38), disagreeableness (*B* = −0.26), impulsivity (*B* = −0.35), and increases in openness (*B* = 0.28) and conscientiousness (*B* = 0.22) at week 6, with neuroticism (*B* = −0.46) remaining decreased at month 6. No significant between-condition differences were observed.

**Conclusions.:**

Personality changes across both conditions were in a direction consistent with improved mental health. With the possible exception of trait absorption, there were no compelling between-condition differences warranting conclusions regarding a selective action of PT (*v*. escitalopram) on personality; however, post-escitalopram changes in personality were significantly moderated by pre-trial positive expectancy for escitalopram, whereas expectancy did not moderate response to PT.

## Introduction

Depression has been ranked by The World Health Organization (WHO) as the fourth leading contributor to the global burden of disease (Reddy, 2010), with a forecast of becoming number one by 2030 (WHO, 2011). In recent years, psychedelic therapy has been increasingly studied as a psychiatric intervention, with ten published clinical trials demonstrating promising efficacy for depressive symptoms (e.g. Carhart-Harris et al., 2016a; Davis et al., 2021; Goodwin et al., 2022; Ross et al., 2016; Sloshower et al., 2023). In one recent trial involving an active comparator and ‘double-dummy’ and double-blind procedures, psilocybin therapy (PT) showed efficacy superior to escitalopram treatment (ET) across most depression outcomes measured – but not the primary outcome (Barba et al., 2022; Carhart-Harris et al., 2021). ET was a combination of escitalopram pharmacotherapy, a selective serotonin reuptake inhibitor (SSRI), psychological support, and two dosing days of low dose (1 mg) psilocybin, whereas PT was a combination of moderate-to-high dose (25 mg) psilocybin, psychological support, and placebo escitalopram capsules. PT’s comparable efficacy to ET on the primary outcome was notable given escitalopram and SSRI medication’s demonstrated efficacy in the treatment of depression (Cipriani et al., 2018; Kennedy, Andersen, & Lam, 2006; Kennedy, Andersen, & Thase, 2009), and the even greater efficacy of combined SSRI pharmacotherapy and psychotherapy (Cuijpers, Dekker, Hollon, & Andersson, 2009), which converges more closely with ET in the present study.

The purpose of the present study was to extend investigation of PT’s treatment effects using the Five-Factor model (FFM; Costa & McCrae, 1992) of personality within the Carhart-Harris et al., 2021 sample. This purpose is considered important for three reasons. First, we agree with others that personality domains bear relevance to mental illness (Kotov et al., 2017; Samuel & Widiger, 2008), and measuring personality could yield new insights on therapeutic mechanisms and psychiatric targets of treatment. Second, personality provides a useful taxonomy for probing with a different, complementary framework, the nature of PT’s *v.* ET’s influence on depression. Third, relative standing on personality domains have been linked to important life outcomes including relationship satisfaction, occupational attainment, and longevity (Ozer & Benet-Martinez, 2006), and as such represent useful outcomes with relevance for treatment evaluation.

### Personality as a signal of relevant treatment targets

The FFM model of personality is a widely-used and well-validated framework for characterizing individual differences in the human population. The FFM was used in the present study because it contains normal-range dimensions that show strong overlap with most symptom dimensions of psychopathology (Thimm, Jordan, & Bach, 2016; Thomas et al., 2013). Such evidence has persuaded many scientists that normal-range personality and psychopathology likely share a common underlying dimensional structure, while occupying different locations along each dimension (Hopwood, Wright, & Bleidorn, 2021). The following *normal-range traits*, *pathological personality symptom dimensions* [from the Alternative Model of Personality Disorder instantiated in DSM-5 (AMPD), containing personality disorders], and *psychopathological spectra* [from the Hierarchical Taxonomy of psychopathology (HiTOP) model, containing DSM-5 clinical and personality disorders] are considered to share the same dimensions (neuroticism <> negative affectivity <> internalizing; extraversion <> introversion <> detachment; openness <> psychoticism <> thought disorder; agreeableness <> antagonism <> antagonistic externalizing; conscientiousness <> disinhibition <> disinhibitory externalizing) (Clark & Watson, 1991; Kotov et al., 2017; Krueger & Markon, 2014).^†1^ Given this overlap, examining the effects of emerging treatments on personality domains holds potential for signaling clinically relevant targets that are often overlooked in favor of a singular target (e.g. major depression). Such examinations could provide preliminary evidence supporting clinical applications for additional spectra of psychopathology.

### Using personality to probe therapeutic response in depression

Personality can additionally provide a useful taxonomy for describing facets of depression and a framework for investigating how therapeutic response may differ by treatment. In the case of major depression, phenotypic evidence supports associations with neuroticism, introversion (or low extraversion), and (low) conscientiousness (Hayward, Taylor, Smoski, Steffens, & Payne, 2013), while genotypic evidence supports associations with neuroticism (primarily) and (low) conscientiousness (Kendler & Myers, 2010). Disagreeableness (or low agreeableness) has also been observed in individuals with persistent depressive symptoms following treatment (Harkness, Michael Bagby, Joffe, & Levitt, 2002).

Despite psychiatry’s dominant understanding of depression as a unidimensional construct, and the predominating use of scale sum-scores as primary outcomes in clinical trials, there is good empirical evidence that depression scales are in fact heterogeneous and reflect multiple symptom dimensions (Bagby, Ryder, Schuller, & Marshall, 2004; Ballard et al., 2018; Fried et al., 2016). The precise degree of heterogeneity varies across scales, but for many scales, it reflects distinct factors containing negative valence (e.g. depressed affect) and positive valence (e.g. hedonic functioning) that notably converge with FFM neuroticism and introversion (Clark & Watson, 1991; Shafer, 2006). Inasmuch as FFM domains usefully parse the heterogeneity of depression, FFM personality could be capable of illuminating which more granular components of depression respond to PT and ET.

### Evidence for personality change accompanying PT and SSRI therapies

Measuring change in personality traits to evaluate the nature of therapeutic action has a long-standing history in clinical psychology (Roberts et al., 2017; Tang et al., 2009). This stream of research incorporates personality trait measures to test the effectiveness of various forms of therapeutic interventions on clinical outcomes, such as depression and anxiety. For example, the efficacy of antidepressant pharmacotherapies containing SSRIs in remediating depression and anxiety may in part be related to reduced neuroticism, introversion, and disagreeableness, and increased conscientiousness. In a large meta-analysis of 81 studies examining pharmacotherapeutic effects on personality, Roberts et al. (2017) observed a large reduction in neuroticism, and small-sized decreases in introversion and disagreeableness and increases in conscientiousness; whereas openness was not observed to exhibit change after performing corrections for small study bias.

By comparison, research examining the effect of psychedelic therapy on personality is scarce, with just one open-label depression trial making this a focus (Erritzoe et al., 2018). The authors observed decreases in neuroticism and introversion, and increases in openness. They interpreted these findings as suggesting that PT may be uniquely advantageous for depressed patients in producing increased extraversion and openness, given that these traits are less sensitive to change with other conventional psychiatric interventions (Roberts et al., 2017).

The present study enables an enhanced test of these findings by comparing PT’s effects on personality to those of ET. Adaptive changes in neuroticism, introversion, and openness are additionally supported by other areas of psychedelic research. For example, despite lacking formal therapeutic interventions, controlled laboratory studies, involving supportive monitoring from clinicians, have observed increases in openness in healthy subjects (Barrett & Griffiths, 2017; Carhart-Harris et al., 2016b; Griffiths et al., 2018; MacLean, Johnson, & Griffiths, 2011; Madsen et al., 2020). More mixed findings of increased conscientiousness and agreeableness have additionally been reported (Barrett & Griffiths, 2017; Schmid & Liechti, 2018).

The ceremonial use of psychedelics may also inform hypotheses about PT efficacy, as ceremony-goers typically show a higher prevalence of psychopathology (40% endorsed a lifetime psychiatric disorder diagnosis; Weiss, Sleep, Miller, & Campbell, 2023), and shamans and facilitators tend to hold a healing-oriented focus. Consistent with Erritzoe et al. (2018), studies examining personality in these ‘naturalistic’ contexts have found substantive increases in openness and decreases in neuroticism, alongside increases in agreeableness and substantial increases in extraversion (though only captured by self-report, not informant-report data) (Netzband, Ruffell, Linton, Tsang, & Wolff, 2020; Weiss, Miller, Carter, & Keith Campbell, 2021). In sum, there is growing evidence from therapeutic contexts that psychedelic therapy may result primarily in adaptive changes to neuroticism, extraversion, and openness.

Direct comparisons between antidepressant pharmacotherapy and PT are difficult to form given differences in the effect size formulas used, but evidence is suggestive that PT-induced changes in extraversion and openness may exceed those of SSRI therapy (e.g. Erritzoe et al., 2018 *v.* Roberts et al., 2017). Introversion has shown particular relevance to depression given its relations to amotivation, anhedonia, social integration (Clark & Watson, 1991; Shafer, 2006), and may be particularly relevant to clinical impairment in relationships, work, and leisure, as e.g., increased extraversion may confer greater energy for interpersonal engagement.

Relative to other personality traits, openness bears comparatively lower relevance to psychological dysfunction (Ozer & Benet-Martinez, 2006) (exception: substance use) and may accordingly be less related to depressive symptomology. Nevertheless, openness alongside extraversion has been linked to the dopaminergic neurotransmitter system (DeYoung, 2013), and therefore may be relevant to depression through enhancing motivation and reward-seeking. Scholars have proposed that trait openness is related to elevated *cognitive exploration* following from the propensity to derive greater incentive value from information and uncertainty in artistic and/or intellectual domains (DeYoung, 2010, 2015). Openness may also be relevant to depression given its association with creative and flexible problem-solving (Chen, He, & Fan, 2022), which could aid in the application of new therapeutic strategies or create opportunities for reward. Openness has also been linked to mystical, transcendental, and transpersonal experiences (MacDonald, 2000) and to forgiveness (Thompson et al., 2005) and inspiration (Thrash & Elliot, 2004), all domains of experience that may be sensitive to psychedelics (Barrett & Griffiths, 2017).

Related to openness is the trait construct of *absorption*. Absorption has been theorized to describe a cognitive disposition toward immersion in one’s internal mental landscape, which can include being engrossed in interior objects of imagination or exterior objects of awareness (e.g. finding meaning in a sunset, experiencing a movie as being real) (Wild, Kuiken, & Schopflocher, 1995). Individuals with high absorption tend to hold paranormal beliefs (Glicksohn & Barrett, 2003), report more vivid spiritual and religious experiences (Lifshitz, van Elk, & Luhrmann, 2019), and are more likely to report extraordinary experiences under a sham ‘god helmet’ condition (Maij & van Elk, 2018). As Lifshitz et al. (2019) observe, ‘absorption…seems to allow the individual to become caught up in their imagination like a daydream and to experience something immaterial as present and real.’ In the context of psychedelics, trait absorption is among the most robust predictors of mystical experience (Aday, Davis, Mitzkovitz, Bloesch, & Davoli, 2021; Haijen et al., 2018; Studerus, Gamma, Kometer, & Vollenweider, 2012), and relates to genetic variation in serotonin 2A receptor functioning (Ott, Reuter, Hennig, & Vaitl, 2005). One important and relatively unexplored question is whether psychedelics produce changes in absorption, and what the health implications of such changes may be.

### Present study

The present study examined changes in personality in relation to PT and ET using a randomized, double-blind, two-arm repeated-measures design. The first objective involved examining change in personality between baseline and *six weeks post-intervention* within PT and ET conditions, separately. Neuroticism, introversion, disagreeableness, and impulsivity were hypothesized to decrease in both treatment conditions, and openness was hypothesized to increase in the PT condition only.

To investigate the long-term effects of the two therapies on personality, the second objective involved examining change in personality between baseline and *six months post-intervention* within the PT and ET conditions, separately. These were explorative analyses and no prior hypotheses were specified.

The third objective was to investigate meaningful differences in personality change between treatment conditions. Neuroticism and introversion were hypothesized to decrease, and openness was hypothesized to increase more in the PT condition.

The fourth objective was to investigate factors that may affect the degree of personality change found in relation to psychedelic experience. Specifically, we examined the degree to which differences in outcomes over time varied as a function of baseline characteristics and acute psychological experiences.

In addition, in view of the relatively high probability that participants become unblinded in a clinical trial examining a drug with conspicuous psychoactive effects such as psilocybin (Aday et al., 2022; Muthukumaraswamy, Forsyth, & Sumner, 2022) and an SSRI with recognized side effects that often also undermine blinding (Lin et al., 2022), we prepared to test the degree to which pre-trial expectancies of favorable response to each condition would account for changes in personality. All hypotheses were preregistered using the Open Science Foundation web platform (https://osf.io/u8r9n).

## Method

### Study design and participants

**Figure 1.**
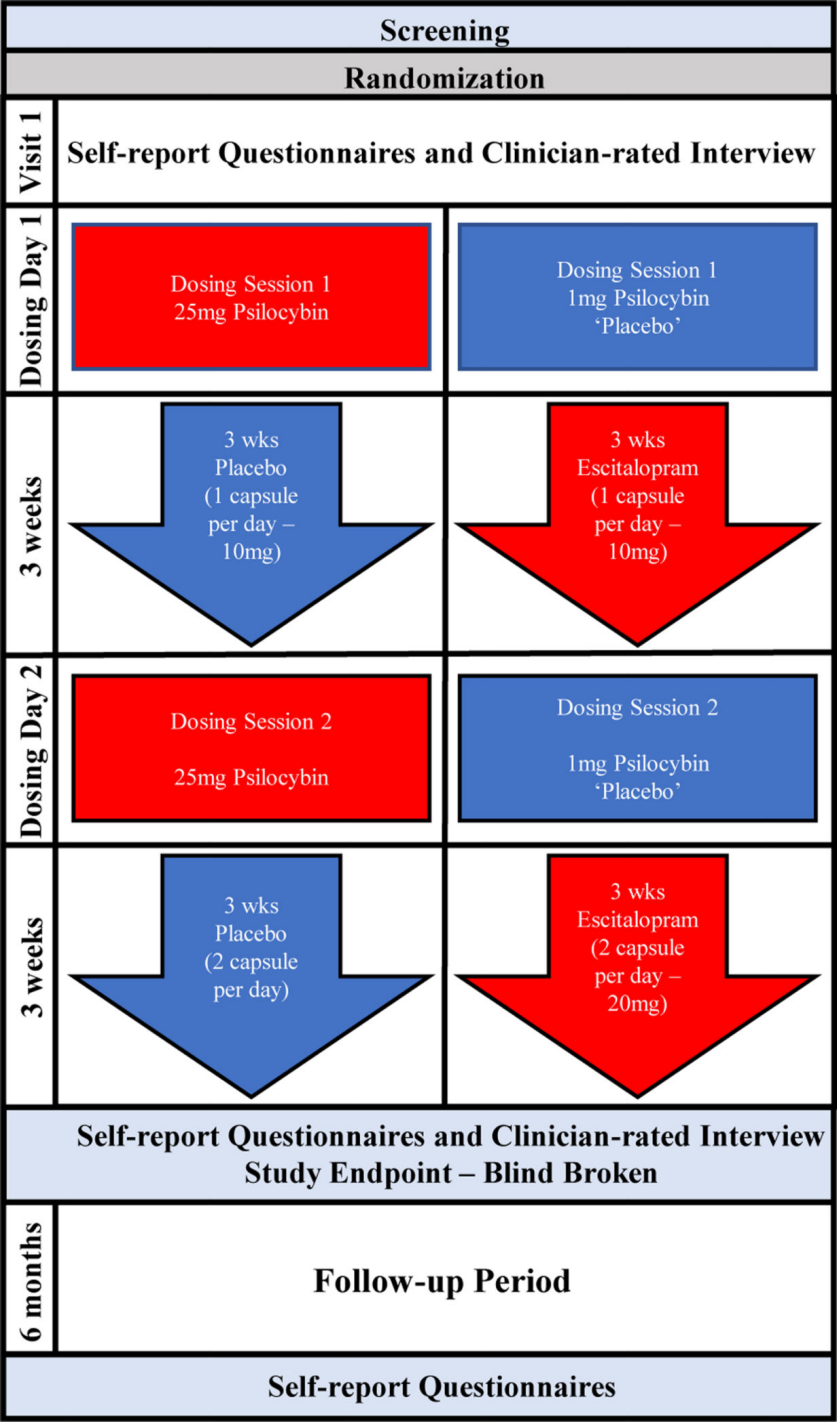
Outline of Study Procedure.

Information regarding trial ethics, patient characteristics, inclusion/exclusion criteria, and study design details can be found in the original Carhart-Harris et al. (2021) article. Briefly, 59 patients with diagnoses of MDD were randomized to either the PT arm (*N* = 30) or the ET arm (*N* = 29). At visit 1 (baseline), all patients completed self-report questionnaires and clinician-rated interviews. At visit 2 (one day after visit 1), the patients in the PT arm received 25 mg of Compass Pathways’ investigational, proprietary, synthetic, psilocybin formulation, i.e., COMP360, and those in the ET arm received 1 mg of psilocybin. All investigators and medication-administering staff were unaware of trial-group assignment. Measures of acute experience were completed after psychedelic effects had subsided. At the end of visit 2, patients received a bottle of capsules and were instructed to take one capsule each morning until their next scheduled day of psilocybin dosing. The capsules contained either microcrystalline cellulose (placebo), which were given to the patients who received the 25 mg dose of psilocybin, or 10 mg of escitalopram, which were given to patients who received the 1 mg dose of psilocybin. Three weeks after the first dosing session (visit 2), patients received their second dose of 25 mg psilocybin or 1 mg psilocybin. Patients again completed measures of acute experience, and were instructed to take two capsules each morning (either placebo in PT arm or an increased dose of 20 mg of escitalopram in the ET arm) for the next three weeks. Following three weeks, the patients returned to complete self-report questionnaires and clinician-rated interviews. We refer to this assessment as *Week 6*, corresponding to the end of patients’ use of escitalopram in the ET condition and three weeks following the last 25 mg psilocybin dosing session for patients in the psilocybin condition. This intervention procedure is presented in Fig. 1.

Psychological support including psychoeducation, therapeutic-alliance-building, and a form of Acceptance and Commitment Therapy (Hayes, Luoma, Bond, Masuda, & Lillis, 2006), namely the Accept/Connect/Embody (ACE) model (Watts & Luoma, 2020) was administered over the course of 11 sessions beginning at screening and ending three weeks after the second dosing day (DD2). A preset music playlist was played to patients during their 4–6 h psychedelic experience.

Six months following this assessment, patients were emailed survey links with additional self-report questionnaires. We refer to this assessment as *Month 6*. Only 21 patients from the ET condition and 25 patients from the PT condition responded at Month 6 follow-up. Groups tended not to differ in their usage of medication, psychedelics, and therapy during the follow-up period (online Supplementary Fig. S1).

### Measures

#### Personality outcomes

##### Big Five Personality Domains.

The Big Five Inventory (BFI) (John & Srivastava, 1999) is a self-report scale designed to measure personality domains including Neuroticism, Extraversion, Openness (to experience), Agreeableness and Conscientiousness. Forty-four items are scored using a 5-point Likert scale ranging from 1 (strongly disagree) to 5 (strongly agree). FFM domains have shown adequate test-retest reliability across an average interval of 4 weeks (Gnambs, 2014). Tests of internal consistency revealed a low correlation between BFI item 4 (depression) and the remaining items at baseline (*r*_drop_ = −0.06). BFI item 4 was accordingly excluded from the Neuroticism domain score. Internal consistency ranged from 0.72 (Agreeableness – Baseline) to 0.88 (Extraversion – Week 6).

The BFI was also assessed at Month 6. However, seven of 44 items failed to be assessed due to administrator error. Missing items included item 39 (Neuroticism), items 40, 41, and 44 (Openness), and item 42 (Agreeableness), and items 38 and 43 (Conscientiousness). For all follow-up analyses (aim 2), FFM domain scores were computed without these items (and BFI item 4 for the above reasons) for baseline, Week 6, and Month 6 timepoints. Internal consistency ranged from 0.72 (Agreeableness – Baseline) to 0.90 (Extraversion – Month 6).

Due to the present study’s clinical context, Extraversion and Agreeableness were reverse-scored and will heretofore be referred to as Introversion and Disagreeableness, respectively.

##### Big Five Aspects Aesthetic Openness and Intellect.

The Big Five Aspects Scale (BFAS) (DeYoung, Quilty, & Peterson, 2007) was used to measure two components of FFM Openness at an intermediate level of the personality hierarchy. Aspects Intellect and Aesthetic Openness were each measured by 10 items using a 5-point Likert scale (1 = Strongly disagree, 5 = Strongly agree). Internal consistency ranged from 0.82 (Aesthetic Openness Baseline) to 0.88 (Intellect Month 6).

The BFAS was also assessed at Month 6. However, two of 20 items failed to be assessed due to administrator error. Missing items included Intellect item 10 and Aesthetic Openness item 6. For all follow-up analyses (aim 2), Intellect and Aesthetic Openness scores were computed without these items for baseline, Week 6, and Month 6 timepoints. Internal consistency ranged from 0.77 (Aesthetic Openness – Month 6) to 0.86 (Intellect – Week 6).

##### Absorption.

The Modified-Tellegen Absorption Scale (MODTAS) (Jamieson, 2005) is a self-report trait measure. Only the 25 scored items were included in this survey. Participants rated each Absorption item on a 5-point Likert-scale (1 = Never, 5 = Very often). Internal consistency was 0.92 (Baseline) and 0.95 (Week 6).

##### Impulsivity.

Barrett Impulsivity Scale-Brief (BIS-B) (Steinberg, Sharp, Stanford, & Tharp, 2013) is an 8-item self-report measure used to index lack of premeditation, a facet of impulsivity that overlaps with (low) FFM Conscientiousness (Whiteside, Lynam, Miller, & Reynolds, 2005). The BIS-B was used in lieu of the full BIS-11 in view of psychometric problems with the original scale involving low internal consistency and multidimensionality (Reise, Moore, Sabb, Brown, & London, 2013). Participants rated items using a Likert-scale (1 = Rarely/Never, 4 = Always). Internal consistency ranged from 0.70 to 0.75 across timepoints.

#### Acute factors

Immediately following two dosing sessions, spaced three weeks apart, patients reported on properties of their acute experience. For each acute variable described below, the *maximum value* was selected from the two dosing sessions to serve as each patient’s score.

##### Mystical experience.

The Mystical Experience Questionnaire (MEQ) (Barrett, Johnson, & Griffiths, 2015; MacLean, Leoutsakos, Johnson, & Griffiths, 2012) is a 30-item scale that measures mystical aspects of participants’ experiences. The MEQ’s items were originally represented on the Pahnke-Richards MEQ (Pahnke, 1969; Richards, 1975). In line with psychometric work (Barrett et al., 2015), four subscales were assessed: Mystical (15-item; e.g. ‘Experience of the fusion of your personal self into a larger whole’), Positive mood (6-item; e.g. ‘Sense of awe or awesomeness’), Transcendence of time and space (6-item; e.g. ‘Loss of your usual sense of space’), and Ineffability (3-item; e.g. ‘Sense that the experience cannot be described adequately in words’). Participants rated each item on a 6-point Likert scale (1 = None, Not at all; 6 = Extreme, more than any other time in your life and stronger than 5). Internal consistency (*α*) ranged from 0.86 (Ineffable dosing day 1) to 0.98 (Mystical dosing day 1).

##### Emotional Breakthrough.

The Emotional Breakthrough Inventory (EBI) (Roseman et al., 2019) is a 6-item scale that measures productive engagement with emotional problems (e.g. ‘I felt able to explore challenging emotions and memories’). Participants rated each item on a visual analog scale ranging from 0 (No, not more than usually) to 100 (Yes, entirely or completely). Internal consistency (*α*) was 0.95 (dosing day 1) and 0.97 (dosing day 2).

##### Emotional Insight.

One item using a visual analog scale (0–100) was used to capture psilocybin patients’ ratings of emotional insightfulness during their dosing sessions (‘Please rate how emotionally insightful the experience was overall’).

##### Intensity.

One item using a visual analog scale (0–100) was used to capture psilocybin patients’ ratings of intensity during their dosing sessions (‘Please rate the overall intensity of the drug effects when the effects were at their most intense’). *Intensity* is regarded to approximate the broad psychoactive and somatic effects of the drug, beyond particular psychological properties such as mystical experience or emotional breakthrough.

#### Expectancy

Treatment response expectancies were measured the day before the first dosing day with two questions asking patients about the degree of improvement they predicted after receiving psilocybin and escitalopram, separately [‘At the end of the trial, after receiving (escitalopram or psilocybin) every day for 6 weeks, how much improvement in your mental health do you think will occur?’]. Each of these variables was measured on a 100-point scale, and will be referred to as *psilocybin therapy expectancy* and *escitalopram expectancy*. Expectancy data was available for 55 patients.

### Analytic plan

Please see full analytic plan in online Supplementary Materials I.

Given the relatively small sample, to balance concerns regarding Type I and Type II error, a statistical significance threshold of *p* < 0.01 was set for non-hypothesized outcomes and *p* < 0.05 for hypothesized outcomes. Some may view these thresholds as overly liberal in view of the large number of analyses. We acknowledge that replication is accordingly critical amidst elevated Type I error. We also applied Benjamini & Hochberg’s (1995) False Discovery Rate (FDR) adjustment to sets of analyses that showed significant results (Benjamini & Hochberg, 1995), and applied the significance thresholds to these FDR-adjusted *p*-values.

Two sets of data were used in the present study: data from patients completing Baseline and Week 6 assessment (*N* = 59), named *dataset A*, and data from those patients who completed Baseline, Week 6, Month 6 (*N* = 46), named *dataset B*.

## Results

### Descriptives of change in personality over time

Table 1 illustrates descriptive mean and standard deviation values of personality outcomes over time, and provides a comparison of these values to mean scores from more representative community- or online-based normative samples. As expected, the present depressed sample exhibited numerically higher standing on personality domains related to depression than normative samples, i.e., higher *Neuroticism*, *Introversion*, *Disagreeableness*, and *Impulsivity*, and lower *Conscientiousness*.

### Examining personality change at week six assessment

These analyses used *dataset A* to examine the degree to which personality changed within the PT and ET conditions, separately, between Baseline and Week 6 assessment. With respect to the PT condition, main effects of time on personality (i.e. personality change) were observed. Six weeks following baseline assessment, *Neuroticism* (*B* = −0.63), *Introversion* (*B* = −0.38), *Disagreeableness* (*B* = −0.47), and *Impulsivity* (*B* = −0.40) were significantly decreased, and *Openness* (*B* = 0.23) and *Absorption* (*B* = 0.32) were significantly increased.

With respect to the ET condition, main effects of time on personality were observed such that *Neuroticism* (*B* = −0.38), *Disagreeableness* (*B* = −0.26), and *Impulsivity* (*B* = −0.35) were significantly decreased, and *Openness* (*B* = .28) was significantly increased, whereas no statistically significant change was observed for either *Introversion* (*B* = −0.20) or *Absorption* (*B* = 0.09).

**Figure 2.**
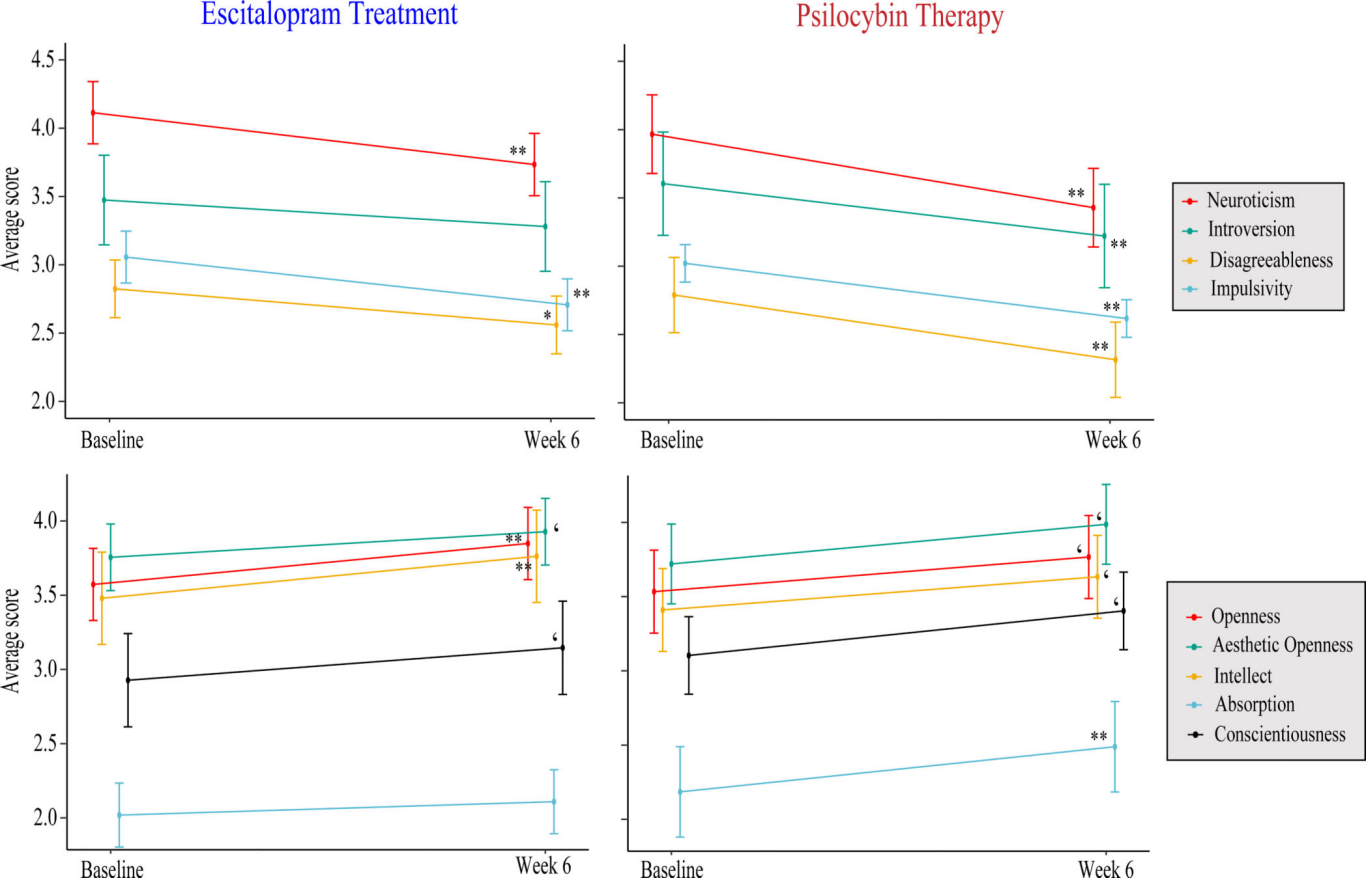
Line plots illustrate self-reported mean changes in personality outcomes between Baseline and Week 6. ET-induced changes are represented on the left, whereas PT-induced changes are on the right. Error bars reflect 95% confidence intervals around the means. ‘*p* < 0.05, *p < 0.01, **p < 0.005.

More detailed results for significant analyses can be found in Table 2. Figure 2 graphically displays the results. Full results can be found in online Supplementary Table S1.

### Examining personality change at month six assessment

These analyses used *dataset B* to examine the degree to which personality changed within the PT and ET conditions, separately, between three timepoints: Baseline, Week 6, Month 6. With respect to the PT condition, a main effect of timepoint was observed on *Neuroticism* [*F*_(2, 48)_ = 5.08, *p* = 0.002], *Introversion* [*F*_(2, 48)_ = 2.01, *p* < 0.003], and *Disagreeableness* [*F*_(2, 48)_ = 3.53, *p* = 0.001], Post-hoc tests demonstrated that six months following intervention, *Neuroticism* (*B* = −0.47) and *Agreeableness* (*B* = 0.41) remained decreased from post-intervention levels.

With respect to the ET condition, main effects of timepoint were observed on *Neuroticism* [*F*_(2, 40)_ = 2.42, *p* = 0.001). Post-hoc tests demonstrated that six months following intervention, *Neuroticism* (*B* = −0.46) remained decreased.

**Figure 3.**
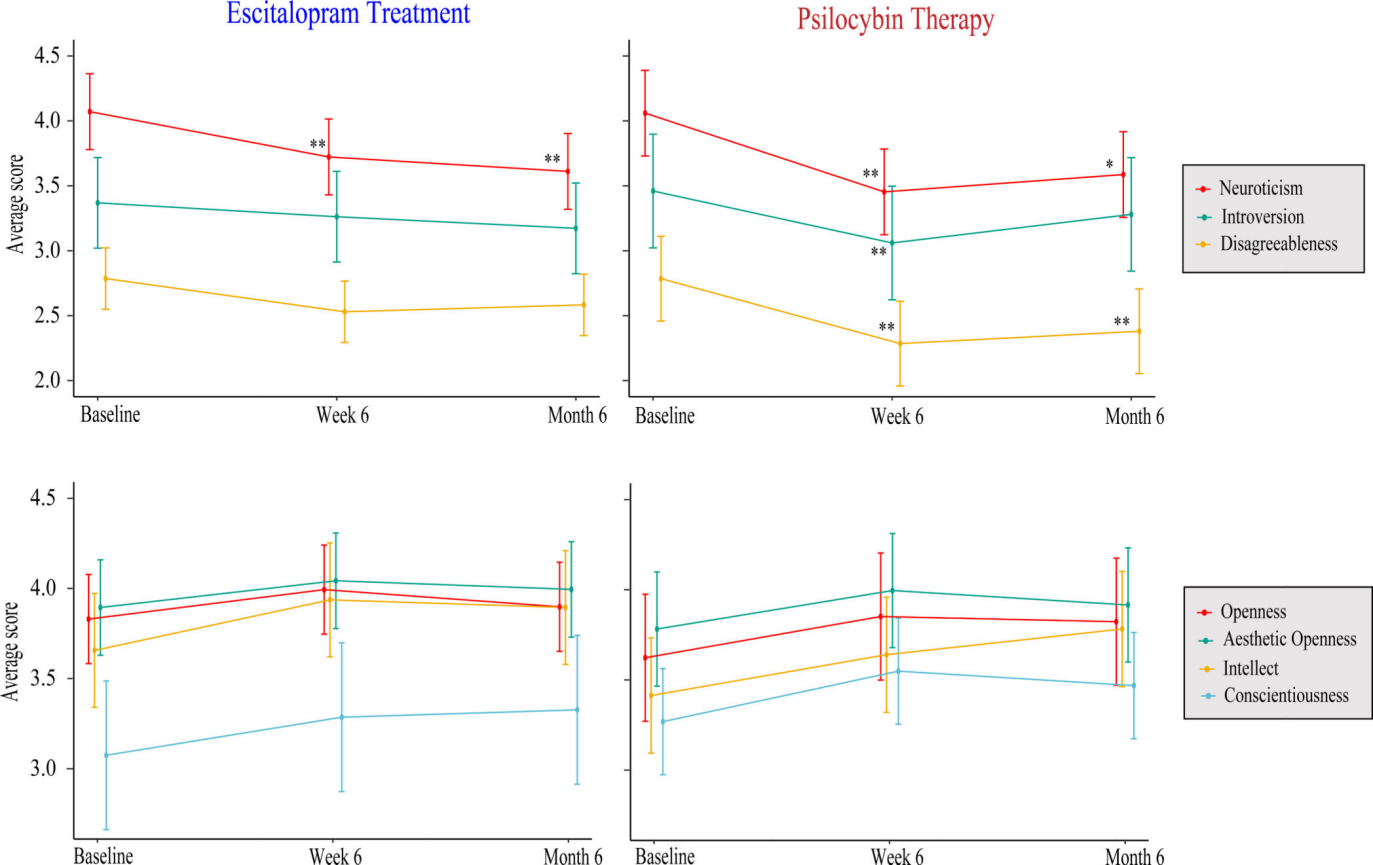
Line plots illustrate self-reported mean changes in personality outcomes between Baseline, Week 6, and Month 6. ET-induced changes are represented on the left, whereas PT-induced changes are on the right. Error bars reflect 95% confidence intervals around the means. *p < 0.01, **p < 0.005.

More detailed results for significant analyses can be found in Table 2. Figure 3 graphically displays the results. Full results can be found in online Supplementary Tables S2 and S3.

### Examining differences in personality change between treatment conditions

These analyses examined between-condition differences in personality change. No statistically significant differences between conditions were observed. However, a trend-level between-condition difference in change in *Absorption* [*B* = 0.23 95% CI (0.04–0.43), *p* = 0.037] emerged. *Absorption* was significant at *p* < 0.05, but not at the more conservative threshold set for non-hypothesized analyses (*p* < 0.01). Full results can be found in online Supplementary Tables S1 and S4.

### Examining moderation of personality change

These analyses used *dataset A* to examine moderation of personality change (within each condition separately) by three sets of variables: *response expectancy* to control for possible positive pre-trial expectancy effects under conditions of imperfect blinding (psilocybin expectancy was used for the PT condition; escitalopram expectancy was used for the ET condition), *baseline characteristics* (including personality, gender, age, unemployment status, education level, previous psychedelic use), and *acute factors* (including MEQ subscales, Emotional Breakthrough, Emotional Insight, Intensity). For expectancy-related analyses, moderation was tested only for personality outcomes that showed significant within-condition change.

First, expectancies of treatment response in depression were examined as potential moderators of within-condition personality change. Full results are provided in online Supplementary Table S5. Contrary to recent critiques hypothesizing an important role for expectancy in biasing response to PT (Muthukumaraswamy et al., 2022), expectancy for PT was not found to moderate pre- *v.* post-PT personality change in this study. Rather, positive expectancy for escitalopram did show evidence of significantly moderating *Neuroticism* and *Conscientiousness* in the ET condition, such that being higher in positive expectancy by one unit (on a 100 unit scale) was associated with an incremental decrease in *Neuroticism* of 0.01 units (*p* = 0.002) and increase in *Conscientiousness* of 0.01 units (*p* = 0.004).

Furthermore, we tested a counterfactual model to address the question of whether change in *Neuroticism* and *Conscientiousness* following at Week 6 would remain significant *if* escitalopram expectancy was set to zero. Results indicated that change in *Neuroticism* and *Conscientiousness* would be non-significant if there had been no positive expectancy for ET.

Second, baseline characteristics and acute factors were examined as moderators of personality change within each condition separately. Three instances of moderation were observed, but a regression to the mean effect could not be ruled out, and thus these results are not interpreted. For detailed results, see online Supplementary Materials III.

## Discussion

PT was associated with decreases in neuroticism, introversion, disagreeableness, and impulsivity, and increases in openness and absorption at study endpoint, whereas ET was associated with decreasesin neuroticism, impulsivity, and disagreeableness, and increases in openness.

The pattern of personality changes within the PT arm are consistent with a robust antidepressant response based on empirical associations between personality and depression. Neuroticism converges quite closely with the cognitive-emotional core of depression, and can be understood as reflecting the shared variance of internalizing disorders (Griffith et al., 2010).

High introversion, or detachment, converges strongly with psychological symptoms of depression including anhedonia, amotivation, and low attention-seeking, and behavioral symptoms of depression including withdrawal and interpersonal passivity (Kotov et al., 2017; Zimmermann, Widiger, Oeltjen, Conway, & Morey, 2022). Non-planning impulsivity, indexed by the measure of impulsivity used in this study (Whiteside & Lynam, 2001), has been psychologically (Swann, Steinberg, Lijffijt, & Moeller, 2008) and neurobiologically (Cowen & Browning, 2015) linked to depression, and could in part contribute to work-related and cognitive dysfunction observed in depression (Birnbaum et al., 2010). Notably, impulsivity showed the strongest standardized effect size change in an adaptive direction – i.e., decreasing by 0.77 standard deviations (in terms of Cohen’s *d* ) post-PT. Disagreeableness has shown a more peripheral relationship to depression, but there is evidence that disagreeable traits are sometimes expressed in the form of distrust and frustration (Harkness et al., 2002), and experience-sampling data has revealed the centrality of anger – bearing a substantial relation to disagreeableness (Costa & McCrae, 1995; Lee & Ashton, 2004) – as a symptom of depression (Fisher, Reeves, Lawyer, Medaglia, & Rubel, 2017). As such, our results were suggestive that most of the personality domains principally associated with depression showed evidence of being impacted by PT therapy.

Furthermore, adaptive alterations in personality showed evidence of being impressively maintained in the follow-up period following PT. Neuroticism remained lower up to six months following PT intervention, and, unlike in the only previous examination of PT-induced personality change, an open-label trial (Erritzoe et al., 2018), we observed a moderately-sized decrease in disagreeableness three weeks post-intervention that appeared to persist six months later.

In addition to examining domains related to depression, the present study expanded upon the open-label trial in observing increases in *aesthetic openness*, *intellect*, and *absorption*. Whereas changes in *openness* in the open-label trial were restricted to facets of openness to actions (indexing preference for variety and creativity) and values (indexing liberalism *v.* conservatism), the present study observed increases in a broader array of aspects and facets. Our results were suggestive that PT enhances cognitive exploration in both esthetic and intellectual domains, and leads to increased cognitive engagement with sensory and imagined phenomena (absorption). Such changes may covary with increases in facets of depression involving anhedonia and amotivation as openness has been linked to dopaminergic systems of wanting and reward (DeYoung, 2013; DeYoung et al., 2011; DeYoung, Peterson, & Higgins, 2005). Greater cognitive exploration may also support psychotherapeutic processes of change involving deeper introspective engagement and motivation, phenomena that have been linked with psychedelic experience in previous work (Watts, Day, Krzanowski, Nutt, & Carhart-Harris, 2017).

Notwithstanding these favorable results, effect size change in personality domains was substantively smaller in the present study than the open-label trial, despite a shorter follow-up period (3 weeks *v.* 3 months post-psilocybin dosing). This pattern was particularly evident for *extraversion* (ds_Erritzoe et al., 2018_ = 0.72 *v.* ds_present_ = 0.27) and *openness* (ds_Erritzoe et al., 2018_ = 0.44 *v.* ds_present_ = 0.22). Differences in estimates may emanate from a variety of sources. The open-label trial included patients with treatment-resistant depression (TRD) whose greater symptom severity on the Quick Inventory of Depressive Symptomatology-Self-Report instrument (Rush et al., 2003) at baseline [mean (S.D.) = 19.2 (2.0)] may have accompanied greater scope for change *v.* the present trial including patients with MDD [mean (S.D.)_baseline_ = 14.5 (3.9)]. The previous trial may also have produced greater effect size change via higher positive expectancy and placebo response by virtue of its open-label design.

In addition to assessing the effect of PT on personality domains, the study also aimed to identify potential differences between PT and ET in personality change. Interaction tests however found no compelling evidence for such differences. A few considerations are nonetheless worth noting. First, although there were no pre-registered hypotheses on absorption change, moderation-based tests were tentatively suggestive of a greater change in this domain with PT than ET. Second, pre-trial positive expectancy was observed to amplify neuroticism reductions in the ET condition, but not the PT condition, suggesting that placebo response localized to the ET condition may have limited our ability to detect a between-condition difference in neuroticism change. Finally, relatively poor statistical power (see Supplementary Materials IV) limited the study’s ability to detect between-condition differences. However, differences between conditions did not exceed *d* = 0.20 across outcomes.

Finally, our results contribute evidence for the effect of SSRI pharmacotherapy + psychological support on personality. Parallel to PT and consistent with previous evidence (Roberts et al., 2017), ET was associated with decreases in neuroticism and impulsivity, and an increase in agreeableness. However, ET was not related to decreased introversion or increased conscientiousness immediately following therapy. Notably, patients reported increased openness, which is not empirically associated with SSRI pharmacotherapy. This result may be suggestive that the psychotherapeutic component of the trial exerted effects on openness, irrespective of drug effect, and raises questions regarding the true source of increased openness in the PT condition.

### Clinical implications

#### A potential treatment alternative to SSRI pharmacotherapy

The present results hold important clinical implications for the utility of PT. Despite failing to show detectable superiority over ET on measured outcomes, PT nevertheless exhibited robust anti-depressant efficacy, extending to a range of personality domains relevant to depression and life functioning. This is notable when indexed against the well-validated benchmark of combined SSRI-based antidepressant therapy and psychological support, which has demonstrated efficacy superior to antidepressant psycho- and pharmacotherapies administered alone (Cuijpers et al., 2009). The proximal implications of these results, should they hold in future research, may be that depressed patients possess an alternative treatment option that removes the need for chronic drug administration, which accompanies problems with medication adherence (Grenard et al., 2011), and avoids common side-effects related to SSRIs such as insomnia, sweating, fatigue (Kirino, 2012), decreased libido (Cascade, Kalali, & Kennedy, 2009; Clayton, Kornstein, Prakash, Mallinckrodt, & Wohlreich, 2007) (see Carhart-Harris et al., 2021 and Weiss, Erritzoe, Giribaldi, Nutt, & Carhart-Harris, 2023 for supportive evidence), and emotional blunting (McCabe, Mishor, Cowen, & Harmer, 2010). Rather, a time-limited regimen consisting of psychological support for six weeks and two dosing days with psilocybin may be sufficient and preferable for some patients. It should be emphasized, however, that our results support the administration of psilocybin in a clinical setting under the care of trained clinicians and guides rather than prescription by a physician and self-administration at home, as involved in SSRI pharmacotherapy. The latter affordance may be more desirable for some patients. Intensive future research would be required to adequately judge whether psilocybin could be safely prescribed and taken home by patients.

#### Additional treatment applications of PT

Our results may also guide inferences about latent targets for PT that extend beyond the construct of depression. Implications include the possible utility of PT for treating disorders characterized by antagonistic externalizing (linked to disagreeableness, e.g. antisocial personality disorder, criminal antisocial acts, violations of rules of conduct) and detachment (linked to introversion, e.g. avoidant personality disorder) – as well as the more conventional targets of internalizing (linked to neuroticism).^2^ Furthermore, disorders characterized by a premeditation-based impulsivity may also be good candidates for PT, including substance use disorder, behavioral addiction (e.g. gambling), and self-harm (Miller, Zeichner, & Wilson, 2012). PT has already shown promising signs of efficacy in treating treatment-resistant cigarette use disorder (Johnson, Garcia-Romeu, Cosimano, & Griffiths, 2014; Johnson, Garcia-Romeu, Johnson, & Griffiths, 2017) and alcohol use disorder (Bogenschutz et al., 2015, 2022).

Other therapeutic targets such as borderline personality disorder and mania may also be relevant to explore (Barker et al., 2015; Swann et al., 2008; Zeifman & Wagner, 2020), though more research is needed to determine the safety of psychedelic therapy for these populations in view of higher degrees of mood lability and self-harm (Gonzalez-Pinto et al., 2006; Oldham, 2006). Given neuroticism’s overlap with the shared variance of internalizing disorders (Griffith et al., 2010), PT’s efficacy may extend to multiple internalizing disorders via a common mechanism (Watson et al., 2022), as well as phenotypes of disinhibiting and antagonistic externalizing disorders wherein externalizing behaviors proximally originate in states of emotional distress (e.g. as in negative urgency within some presentations of substance use disorder, Smith & Cyders, 2016). We suggest that the present results guide enhanced investment in research exploring PT’s utility across these relevant mental health disorders.

#### Unique benefits of PT

Our results may also hold important implications for how PT remediates depression. Given introversion/extraversion’s overlap with positive emotion, sociability, energy, and agency, therapies that differentially decrease introversion (or increase extraversion) may be particularly relevant in remediating symptoms of anhedonia and amotivation, and may also furnish the energy and inclination for exploring new environmental rewards. Given evidence that patients with persistent depression are disproportionately higher in disagreeable traits (Harkness et al., 2002), therapies that resolve disagreeableness may be clinically useful for longer-term and treatment-resistant profiles of depression. In addition, when considering these effects in therapeutic context, it may be useful to understand that whereas adaptive changes in neuroticism and disagreeableness have shown longer-term maintenance, adaptive changes in introversion may be more time-limited (c.f. Erritzoe et al., 2018).

#### Possible benefits and risks related to absorption

PT’s influence on absorption may also bear important health-related implications. On one hand, enhanced absorption may be beneficial as absorption is associated with novel engagement with imagined and exterior sensory and affective experiences, vivid spiritual experience (Lifshitz et al., 2019), and creative style and capacity (Manmiller, Kumar, & Pekala, 2005).

On the other hand, there is a small body of literature that links absorption, albeit weakly, to thought disorder and psychoticism (Gore & Widiger, 2013; Perona-Garcelán et al., 2016; Rosen et al., 2017), schizotypal personality (*r* = 0.23) (Coolidge, Segal, Cahill, & Archuleta, 2008), and anxiety sensitivity (Lilienfeld, 1997). Convergent with these findings is recent evidence that the shared variance between esthetic openness and psychoticism (which is likely to overlap with absorption) (DeYoung, 2015) are empirically linked to apophenia, indexed by false-positive errors on multiple behavioral tasks (Blain, Longenecker, Grazioplene, Klimes-Dougan, & DeYoung, 2020). Absorption’s association with apophenia, a tendency toward implausible pattern detection, may also be consistent with a recent study that observed higher absorption participants to report a higher frequency of extraordinary experiences while wearing a sham helmet (Maij & van Elk, 2018). A disposition toward false-positive pattern detection could be advantageous in certain domains (e.g. art, spirituality, invention), but some scholars have raised concerns about maladaptive effects, especially when expressed in the absence of intelligence, subserving reality-testing (Blain et al., 2020). For example, such a disposition has been proposed as a potential risk factor for the development of psychosis (Blain et al., 2020). We support future research examining psychedelics’ contribution to psychosis risk, especially in view of empirical suggestions of links thereof (Kuzenko et al., 2011; Weiss et al., 2023). However, we do not wish to conflate absorption with psychopathology. As Lifshitz et al. (2019) also caution, cognition is only psychopathological to the degree that it accompanies distress or impairment. Importantly, we refer the reader to studies that have not shown evidence of emergent psychosis following psychedelic experience (Carhart-Harris et al., 2016a; Carhart-Harris et al., 2016b; Krebs & Johansen, 2013).

### Response expectancy observed for ET condition

Analyses adjusting for pre-trial expectancy found evidence of substantial moderation of changes in neuroticism via positive expectancy for escitalopram – and this moderation was exclusive to the ET condition i.e., not observed in the PT condition. Specifically, higher initial expectancy about the efficacy of escitalopram for remediating depression was associated with greater reported decreases in neuroticism, and estimates from a counterfactual model were suggestive that significant change in neuroticism would not be present among patients had there been no positive expectancy for escitalopram. This asynchrony in response expectancy was surprising given that expectancy effects have been observed across a number of pharmacological and psychotherapeutic treatments (Bingel et al., 2011; Hjorth et al., 2021; Tambling, 2012) including psychedelic modalities (Weiss et al., 2021), and both SSRIs and psilocybin are associated with substantial levels of unblinding even within double-blind randomized controlled trials (Muthukumaraswamy, Forsyth, & Lumley, 2021; Scott, Sharpe, & Colagiuri, 2022). Why expectancy response was not observed in the PT arm is not clear. Variance in pre-trial expectancy was not lower in the PT *v.* ET arm, and there was no evidence of a ceiling effect in PT arm scores, e.g., mean expectancy was 54% out of 100%. As a speculative explanation that requires further research, the acute experience related to PT may have been sufficiently psychologically dramatic as to disrupt the implicit effects of expectancy that would have obtained had the treatment been more subtle. Another relates to the fact that all patients endorsed non-zero positive pre-trial expectancy; it is conceivable that a hidden non-linear effect of expectancy obtained such that PT patients in the trial tended not to meaningfully differ in expectancy response regardless of their positive expectancy rating, but had PT patients been included endorsing zero expectancy, these patients could have exhibited lower expectancy response. Nevertheless, a potential implication of these findings is that ET’s causal influence on neuroticism is to some degree called into question, especially in a study wherein patients were likely to know they were in the SSRI condition.

### A critical look at between-condition differences

According to interaction tests, between-condition differences in personality change were not statistically significant, though sub-optimal statistical power in a small sample undoubtedly contributed to lower sensitivity (see online Supplementary Materials IV for sensitivity power analyses). One interpretation of the present findings therefore is that PT and ET have comparable effects on personality.

We are cautious to draw such conclusions prematurely, however, in view of three considerations. First, limits on statistical power make replications in larger samples necessary. Second, as just mentioned, positive expectancy (otherwise known as the ‘placebo effect’) may have disproportionately affected the SSRI condition, raising the possibility of PT’s superiority in reducing neuroticism.

Third, we failed to hypothesize a between-condition difference in change in absorption within our preregistration [erroneously given its empirical overlap with openness (Glisky, Tataryn, Tobias, Kihlstrom, & McConkey, 1991)]. We accordingly refrain from concluding a between-condition difference here, but encourage future researchers to examine absorption in future clinical trials involving PT, and submit the hypothesis that increases in trait absorption will be greater for PT than for SSRI + psychotherapy treatment. If PT were found to differentially increase trait absorption, this would have important implications because increased absorption may either present a benefit to receptive patients, e.g., being predictive of greater therapeutic improvement, or confer iatrogenic risk, e.g., by promoting traits related to psychoticism.

### Limitations

A number of limitations should be noted. First, our conclusions with respect to between-condition differences in personality change are severely limited by low statistical power associated with small sample-size. Our sensitivity power analyses demonstrate that we were not powered to detect a difference between conditions that would be considered meaningful. Second, expectancy was measured only with respect to favorable improvement in depression, and thus represents an imperfect measure of expectancy with respect to trait changes outsideneuroticism. Expectancy results should accordingly be cautiously interpreted. Third, assessments of long-term change are restricted to FFM outcomes, but not absorption or impulsivity. Finally, as all trial interventions ended at week 6, several confounding factors extraneous to the trial interventions could have influenced outcomes at month 6. We therefore advise caution when drawing inferences about this timepoint.

### Future directions

Our findings logically lead to a number of future lines of research. Methodologically, we believe there is value in (1) replicating this study with a larger sample to enable detection of smaller (but still clinically meaningful) between-condition differences; (2) implementing periodic tests of expectancy and blind-breaking throughout the study period (Muthukumaraswamy et al., 2021); (3) employing expectancy measures tailored to the personality domains under study; and (4) adapting global personality measures (i.e. referring to questions about general psychological and behavioral patterns) to be retrospective over short time-periods (e.g. 3 h and/or 1 day) to afford greater sensitivity to change, in line with calls for more ecologically valid intensive longitudinal measurement (Wright & Zimmermann, 2019) and the density distribution conceptualization of personality (Fleeson, 2001).

Clinically, there is value in using these findings as a basis for (1) designing clinical trials examining psychedelic therapeutic applications for putatively responsive clinical phenotypes identified in the present work, e.g., behavioral addiction, self-harm, antisocial personality disorder, involving impulsivity and disagreeableness; and (2) probing the effect of PT on trait absorption as well as evaluating the risks and benefits associated with such increases.

Finally, this study was focused exclusively on psychological outcomes and did not examine potential neurobiological mechanisms relating to the observed personality changes. Recent neuroimaging findings from the present trial’s cohort and a previous TRD trial of ours revealed decreased brain network modularity post-PT in both trials that correlated with decreases in symptom severity in both independent samples (Daws et al., 2022). Merging the Research Domain Criteria (Cuthbert & Insel, 2013) and HiTOP approaches to psychopathology (Kotov et al., 2017) may invite hypothesis testing regarding relationships between post-PT changes in network modularity or alternative imaging metrics and related changes in high-level domains of psychopathology (Romer et al., 2021).

## Conclusion

Despite the demonstrated efficacy of SSRI pharmacotherapies, alternative treatments that avoid known side-effects (Cascade et al., 2009) and show superior benefit (e.g. exercise Belvederi Murri et al., 2019) warrant greater study. A personality framework was used to examine the responsiveness of components of depression and different areas of psychopathology to PT *v.* ET therapies. PT was observed to produce a more robust antidepressant response, involving decreases in neuroticism, introversion, disagreeableness, and impulsivity, compared to decreases in neuroticism, disagreeableness, and impulsivity (related to ET). However, no formal differences in the magnitude of response was detected between the therapies.

## Supplementary Material

Supplementary Materials

Supplementary Figure 1

## Figures and Tables

**Table 1.** Descriptive changes in personality over time and comparison to normative data

Outcome	Normative sample Mean (S.D.)	Baseline Mean (S.D.)	Week 6 Mean (S.D.)	Month 6 Mean (S.D.)
Escitalopram Treatment				
Neuroticism	3.17 (0.84)^a^	4.11 (0.58)	3.73 (0.63)	3.61 (0.65)
Introversion	2.77 (0.91)^a^	3.47 (0.86)	3.28 (0.88)	3.17 (0.71)
Openness	3.88 (0.69)^a^	3.57 (0.66)	3.85 (0.63)	3.90 (0.57)
Disagreeableness	2.10 (0.66)^a^	2.82 (0.58)	2.56 (0.55)	2.58 (0.52)
Conscientiousness	3.75 (0.71)^a^	2.93 (0.84)	3.15 (0.82)	3.33 (0.89)
Aesthetic Openness	4.04 (0.64)^b^	3.76 (0.63)	3.93 (0.56)	3.99 (0.53)
Intellect	4.01 (0.61)^b^	3.48 (0.84)	3.76 (0.80)	3.89 (0.68)
Impulsivity	1.69^c^	3.06 (0.48)	2.71 (0.53)	
Psilocybin Therapy				
Neuroticism	3.14 (0.88)^d^	3.97 (0.74)	3.43 (0.83)	3.59 (0.81)
Introversion	2.78 (0.93)^d^	3.60 (1.00)	3.22 (1.04)	3.28 (1.19)
Openness	3.88 (0.72)^d^	3.53 (0.74)	3.77 (0.77)	3.82 (0.84)
Disagreeableness	2.17 (0.67)^d^	2.79 (0.75)	2.31 (0.75)	2.38 (0.82)
Conscientiousness	3.75 (0.70)^d^	3.10 (0.73)	3.40 (0.69)	3.47 (0.80)
Aesthetic Openness	4.04 (0.64)^b^	3.72 (0.69)	3.99 (0.77)	3.92 (0.70)
Intellect	4.01 (0.61)^b^	3.41 (0.76)	3.63 (0.75)	3.78 (0.78)
Impulsivity	1.69^c^	3.02 (0.40)	2.62 (0.34)	

*Note.* Unstandardized mean mean-scores and standard deviations (S.D.) of mean-scores for each treatment arm are provided for each timepoint. Most outcomes used a 5-point Likert-scale (1–5), whereas Impulsivity used a 4-point Likert-scale (1–4). The values for Neuroticism reflect all items rather than the shortened version used in other analyses in order to compare with the normative values. Month 6 values for Impulsivity are not provided as Impulsivity was not administered at this timepoint.

For normative data:

a values are drawn from an online sample whose age (39 years) matches the mean age of the ET arm (Srivastava, John, Gosling, & Potter, 2003, *N* = 1269);

b values are drawn from a community sample (DeYoung, Carey, Krueger, & Ross, 2016, *N* = 321);

c values are drawn from a community sample (Steinberg et al., 2013, *N* = 128), S.D. are not presented as S.D. values were not included in the article;

d values are drawn from an online sample whose age (43 years) matches the mean age of the PT arm (Srivastava et al., 2003, *N* = 1064).

**Table 2.** Significant within-condition changes in personality outcomes

Outcome	Time	*B*	Confidence Interval	*dz*	*ds*
Escitalopram Treatment					
Neuroticism	Baseline & Week 6	−0.38**	95%(−0.53 to −0.23)	−0.92	−0.44
Neuroticism	Baseline & Month 6	−0.46**	99% (−0.76 to −0.16)	−0.89	−0.51
Openness	Baseline & Week 6	0.28**	99% (0.04–0.51)	0.59	0.30
Intellect	Baseline & Week 6	0.28**	99% (0.06–0.50)	0.64	0.24
Disagreeableness	Baseline & Week 6	−0.26*	95% (−0.45 to −0.08)	−0.53	−0.33
Impulsivity	Baseline & Week 6	−0.35**	95% (−0.22 to −0.48)	−0.99	−0.49
Psilocybin Therapy					
Neuroticism	Baseline & Week 6	−0.54**	95% (−0.82 to −0.26)	−0.70	−0.49
Neuroticism	Baseline & Month 6	−0.47*	99% (−0.92 to −0.03)	−57	−0.42
Introversion	Baseline & Week 6	−0.38**	95% (−0.57 to −0.19)	−0.73	−0.27
Openness	Baseline & Week 6	0.23^′^	95% (0.05–0.42)	0.46	0.22
Aesthetic Openness	Baseline & Week 6	0.27^′^	95% (0.05–0.48)	0.45	0.26
Intellect	Baseline & Week 6	0.22^′^	95% (0.03–0.41)	0.43	0.21
Absorption	Baseline & Week 6	0.30**	99% (0.11–0.50)	0.78	0.26
Disagreeableness	Baseline & Week 6	−0.47**	95% (−0.69 to −0.26)	−0.81	−0.45
Disagreeableness	Baseline & Month 6	−0.41**	99% (−0.74 to −0.07)	−0.67	−0.35
Impulsivity	Baseline & Week 6	−0.40**	95% (−0.30 to −0.51)	−1.39	−0.77

*Note.* Unstandardized (B) coefficients indicate mean differences between timepoints. *dz* indicates effect size change in outcome scores in terms of the standard deviation of within-subject change scores (e.g. T2-T1; Lakens, 2013). Cohen’s *ds* (standard Cohen’s d; Cohen, 1988) effect size estimates were calculated using the following equation: (Mean-score_T2_ – Mean-score_T1_)/[(SD_T1_)2 + SD_T2_)2] 0.5.

′ *p* < 0.05

* *p* < 0.01

** *p* < 0.005.

## Footnotes

† The notes appear after the main text.

1 According to this growing consensus, personality and psychopathology are regarded to share content and structure, with the important caveat that psychopathology is reserved for persons for whom higher standing in a trait’s maladaptive range attends person-environment dysfunction, i.e., mismatch of goals/behavior to situational demands, e.g., occupational problems related to depression, deceit/conflict impacting one’s capacity for stable relationships (Hopwood et al., 2021).

2 See (Nutt, Erritzoe, & Carhart-Harris, 2020) for relevant inferences from a cognitive neuroscientific perspective.
