# Supplementary material for: Personality change in a trial of psilocybin therapy v. escitalopram treatment for depression
Source: Psychol Med. Author manuscript; Available in PMC 2024 Feb 16. (PMC10692311; doi:10.1017/S0033291723001514)
Supplement: Supplementary Materials [file NIHMS1932967-supplement-Supplementary_Materials.docx]

**Supplementary Materials**

**Supplementary Materials I. Analytic Plan**

***Analyses***

Five sets of analyses were planned. Given the relatively small sample, to balance concerns regarding Type I and Type II error, a statistical significance threshold of *p* < .01 was set for non-hypothesized outcomes and *p* < .05 for hypothesized outcomes. Some may view these thresholds as overly liberal in view of the large number of analyses. We acknowledge that replication is accordingly critical amidst elevated Type I error. We also applied Benjamini & Hochberg’s (1995) False Discovery Rate (FDR) adjustment to sets of analyses exhibiting significant results Benjamini and Hochberg (1995).

All data met assumptions of normality according to guidelines suggested by Hair et al. (2019) and Byrne (2013).

Two sets of data were used in the present study: data from patients completing Baseline and Week 6 assessment (N = 59), named *dataset A*, and data from those patients who completed Baseline, Week 6, Month 6 (N = 46), named *dataset B*.

The first set of analyses (dataset A) examined the degree to which personality changed within the Psilocybin Therapy (PT) and Escitalopram Treatment (ET) conditions, separately, between Baseline and Week 6 assessment. Linear mixed effects (LME) models were conducted (equivalent to one-way repeated measures analysis of variance [ANOVA]) using R software (‘lme4’ package) to determine the effect of intervention on outcomes. Specifically, each personality outcome was regressed onto Time, with a random effect of intercept specified. Separate models were run for each outcome.

Accordingly, the mathematical model and R code are presented below:

| Mathematical model | Level 1: Yij = β0j + β1j(Time)j + rij  Level 2: β0j = γ00 + u0j (random intercept)  Level 2: β1j = γ10 |
| --- | --- |
| R code formula | lmer(Y ~ Time + (1 \| participant) , data = data frame) |

where Yij = level of *outcome* for person j at timepoint (categorical variable with levels of Baseline, Week 6). Unstandardized (B) coefficients indicate mean differences between timepoints. Cohen’s *d* effect sizes were calculated using the formula: (e.g., Mean-score_T2_ -Mean-score_T1_)/((SD_T2_)+(SD_T1_)2)0.5 where standard deviation is pooled. Cohen’s *dz* effect size estimates were derived by dividing the mean difference of personality scores between two time points by the standard deviation of the difference scores.

The second set of analyses (dataset B) mirrored the first set but examined the degree to which personality changed within the PT and ET conditions, separately, between three timepoints: Baseline, Week 6, Month 6. ANOVA within an LME framework (i.e., ‘lme4’ package) was conducted to examine the presence of significant differences between any two of three timepoints. Where a significant main effect was observed, post-hoc comparisons were conducted between Baseline and Week 6, and between Baseline and Month 6.

The third set of analyses used LME models to examine between-condition differences in personality change. LME models included data from both conditions, with each outcome separately regressed onto the interaction between Condition and Time.

Accordingly, the mathematical model and R code are presented below:

| Mathematical model | Level 1: Yij = β0j + β1j(Time)j + rij  Level 2: β0j = γ00 + γ01(Condition)j + u0j (random intercept)  Level 2: β1j = γ10 + γ11(Condition)j |
| --- | --- |
| R code formula | lmer(Y ~ Timepoint*Condition + (1 \| participant) , data = data frame) |

where Yij = level of *outcome* for person j at timepoint (categorical variable with levels varying by dataset used). For dataset A, unstandardized coefficients were used to index incremental change in the PT condition versus the ET condition. For dataset B, ANOVA within an LME framework was used to evaluate whether the conditions significantly differed in the amount of change between any two of the three timepoints.

The fourth set of analyses replicated the third set of analyses using a smaller subsample of dataset A containing patients matched between conditions on the basis of QIDS depression improvement between Baseline and Week 6 (Tang et al., 2009). The matching operation and results are provided in Supplementary Materials II.

The fifth set of analyses used LME models to examine moderation of personality change (within each condition separately) by three sets of variables: *expectancy* to control for possible positive expectancy effects under conditions of imperfect blinding (psilocybin therapy expectancy was used for the psilocybin therapy condition; escitalopram expectancy was used for the ET condition), *baseline characteristics* (including personality, gender, age, unemployment status, education level, previous psychedelic use), and *acute factors* (including MEQ subscales, Emotional Breakthrough, Emotional Insight, Intensity). For expectancy-related analyses, moderation was tested only for personality outcomes that showed significant within-condition change. Due to concerns regarding sample size and statistical power, only dataset A was used for these analyses. Separate models were conducted for each outcome.

Accordingly, the mathematical model and R code are presented below:

| Mathematical model | Level 1: Yij = β0j + β1j(Time)j + rij  Level 2: β0j = γ00 + γ01(Moderator)j + u0j (random intercept)  Level 2: β1j = γ10 + γ11(Moderator)j |
| --- | --- |
| R code formula | lmer(Y ~ Timepoint*Moderator + (1 \| participant) , data = data frame) |

where Yij = level of *outcome* for person j at timepoint (categorical variable with levels of Baseline, Week 6). Moderators were left unstandardized, and unstandardized coefficients were used to estimate the added effect of moderators to the effect of time with an increase of one unit in the moderator.

***False Discovery Rate adjustment***

An FDR p-value adjustment was applied to two sets of analyses exhibiting significant results: within-condition differences in personality following six weeks (dataset A), and within-condition differences in personality between baseline, six-weeks, and six months (dataset B; ANOVA analyses only). This adjustment did not change the pattern of significance among the results. Adjusted p-values can be found in Supplementary Tables 1 and 2.

**Supplementary Materials II. Between-condition change in personality controlling for depression improvement**

***Measures for Matching Analysis***

*Depression.* Quick Inventory of Depressive Symptomatology–Self-Report (QIDS-SR 16) (Rush et al., 2003) was the primary depression measure used in the original clinical trial (Carhart-Harris et al., 2021). QIDS change scores (indexing the difference in QIDS sum score between baseline and week 6 [study endpoint]) were used to match participants in terms of improvement in depression between the PT and ET conditions.

***Matching technique***

Matching occurred in three steps. First, patients were matched directly on depression improvement. Second, patients were matched who differed to an equivalent degree in depression improvement by one unit (e.g., PT patients A and B with depression improvement score of -2 and -8 were matched with ET patients C and D with scores of -3 and -7). Third, patients were matched who differed to an equivalent degree by two units. If a match could not be found, participants were dropped. Twenty-one PT patients and 21 ET patients were successfully matched using this technique. The same linear mixed effects models were applied for this final set of analyses to examine how personality changed between the conditions whilst controlling for depression improvement.

***Results***

No statistically significant differences in personality change between conditions were observed. Results are provided in Supplementary Table 1 by the “Interaction by Condition (matched sample)” heading.

**Supplementary Materials III. Moderation by baseline characteristics and acute factors**

To examine the degree to which expectancy moderated changes in personality, LME models were conducted that separately regressed personality outcome scores onto the interaction of Time and each baseline characteristic and acute factor variable, separately, within each condition separately. Baseline characteristics included gender, age, unemployment status, education level, previous psychedelic use, and baseline personality scores. Acute factors included Mystical Experience Questionnaire subscales, emotional breakthrough, emotional insight, and intensity.

Three instances of moderation by baseline characteristics were observed at *p* < .01. First, in the ET condition, change in *Aesthetic Openness* (B*_Time x Baseline Aesthetic Openness_* = -.26*, p* = .009) and *Agreeableness (Disagreeableness)* (B*_Time x Baseline Agreeableness_* = -.43*, p* = .004) showed evidence of being moderated by their respective scores at baseline. For example, being one unit lower in *Aesthetic Openness* at baseline was associated with an incremental .26 unit increase in *Aesthetic Openness* following six weeks.

Second, in the PT condition, change in *Impulsivity* showed evidence of being moderated by its respective score at baseline (B*_Time x Baseline Impulsivity_* = -.32*, p* = .005). Specifically, being one unit higher in *Impulsivity* at baseline was associated with an incremental .32 unit decrease in *Impulsivity* following six weeks.

However, because regression to the mean could be responsible for these trends, subsequent tests were conducted. Effect size estimates were examined while excluding participants with extreme baseline scores most conducive to regression in an adaptive trait direction, namely above the 80th quantile of baseline *Impulsivity* and below the 20th quantile of *Aesthetic Openness* and *Agreeableness (Disagreeableness)*. Due to the small sample size, however, reducing the sample size by 20% resulted in negative eigenvalues for two of the three models. Although the moderation model containing baseline *Impulsivity* converged, results showed non-significant moderation at the *p* < .01 threshold (B*_Time x Baseline Impulsivity_* = -.38*, p* = .016), and the pattern of results was unusual, e.g., the adulterated model showed an overall increase in *Impulsivity* rather than an increase. Given that regression to the mean could not be rigorously ruled out as an explanation for the present findings, these results will not be interpreted, but the present authors encourage replication in future work. Full results are provided in Supplementary Table 6.

In addition, among the moderation-based analyses, the following models contained negative eigenvalues or triggered singularity issues:

- Escitalopram Treatment condition
  - Moderation of change in BFI Neuroticism by BFI Neuroticism
  - Moderation of change in BFAS Openness by BFAS Intellect
  - Moderation of change in BFAS Intellect by Previous Psychedelic Use
  - Moderation of change in BFI Openness by baseline BFI Extraversion/Introversion
- Psilocybin Therapy condition
  - Moderation of change in BFI Neuroticism by baseline BFI Neuroticism
  - Moderation of change in BFAS Openness by baseline BFAS Intellect
  - Moderation of change in BFAS Openness by Previous Psychedelic Use
  - Moderation of change in BFAS Openness by baseline BFAS Openness
  - Moderation of change in BFAS Intellect by baseline BFAS Intellect
  - Moderation of change in BFI Agreeableness/Disagreeableness by baseline BFI Agreeableness/Disagreeableness
  - Moderation of change in BFI Conscientiousness by baseline BFI Conscientiousness
  - Moderation of change in BFI Conscientiousness by baseline BFI Agreeableness/Disagreeableness
  - Moderation of change in BFI Conscientiousness by baseline BFI Openness
  - Moderation of change in BFI Conscientiousness by baseline BFI Extraversion/Introversion

**Supplementary IV. Sensitivity Power Analyses**

Post hoc sensitivity power analyses (using “simr” package in R) were conducted to assess power for the four sets of analyses. For each set of analyses, effect sizes sufficient to obtain 80% statistical power (alpha value *p* = 0.01 [*p* = 0.05 for hypotheses], using 100 Monte Carlo simulations) were estimated. For the first set of analyses (dataset A), results indicated that the PT sample was powered (80%) to accurately detect true differences between timepoints of medium to large size. Effect sizes ranged from .28 (*Agreeableness/Disagreeableness*) to .59 (*Conscientiousness*) standard deviations. The ET sample was powered (80%) to accurately detect true differences between timepoints of medium size. Effect sizes ranged from .32 (*Extraversion/Introversion*) to .45 (*Agreeableness/Disagreeableness*) standard deviations.

For the second set of analyses, results indicated that the PT sample was powered (80%) to accurately detect true differences between timepoints of medium to large size. Effect sizes ranged from .35 (*Extraversion*) to .75 (*Intellect*) standard deviations. The ET sample was powered (80%) to accurately detect true differences between timepoints of medium to large size. Effect sizes ranged from .45 (*Conscientiousness*) to .80 (*Intellect*) standard deviations.

For the third and fourth sets of analyses and dataset A, results indicated that the sample was powered (80%) to accurately detect between-condition differences in personality change of large size. Interaction effect sizes (*Time* x *Condition*) ranged from .40 (*Extraversion/Introversion*) to .70 (*Aesthetic Openness*) standard deviations. For dataset B, results indicated that the sample was powered (80%) to accurately detect between-condition differences in personality change of large to very large size. Interaction effect sizes (*Time* x *Condition*) ranged from .57 (*Extraversion/Introversion*) to 1.15 (*Intellect*) standard deviations.

For the fifth set of analyses, expectancy, baseline characteristics, and acute factors were examined separately. For expectancy variables, analyses were powered (80%) to detect true interaction effects of large size. Interaction effect sizes (*Time* x *Expectancy*) ranged from .26 (*Absorption* ~ *Time* x *Psilocybin expectancy*, PT condition) to .50 (*Agreeableness/Disagreeableness* ~ *Time* x *Escitalopram expectancy*, ET condition). The alpha value was set a *p* < .05 for these expectancy power analyses.

For baseline characteristics, continuous and categorical variables were examined separately. For continuous variables, analyses were powered (80%) to detect true interaction effects of large size. Interaction effect sizes (*Time* x *Baseline characteristic*) ranged from .30 (*Absorption* ~ *Time* x *Baseline Neuroticism*, PT condition) to .62 (*Neuroticism* ~ *Time* x *Baseline Conscientiousness*, PT condition). For categorial variables, namely *Gender*, analyses were powered (80%) to detect true interaction effects of large size. Interaction effect sizes (*Time* x *Gender*) ranged from .62 (*Absorption* ~ *Time* x *Gender*, PT condition) to 1.3 (*Agreeableness/Disagreeableness* ~ *Time* x *Gender*, ET condition).

For acute factors, analyses were powered (80%) to detect true interaction effects of large size. Interaction effect sizes (*Time* x *Acute factor*) ranged from .30 (*Absorption* ~ *Time* x *MEQ Ineffable*, PT condition) to .50 (*Openness* ~ *Time* x *Intensity*, ET condition).

Overall, sensitivity power analyses were suggestive of relatively low statistical power. Analyses were generally powered to detect true differences between timepoints of medium to large size (first set of analyses), and incremental moderation-driven differences between timepoints of large size (second, third, and fourth sets of analyses).

Supplementary Table 1

Examining within-condition changes in personality and between-condition changes in personality between Baseline and Week 6 (dataset A)

| Analysis | | Parameter | B | Std Error | df | t-value | Confidence  Interval | p-value | FDR adjusted  p-value | *dz* | *ds* |
| --- | --- | --- | --- | --- | --- | --- | --- | --- | --- | --- | --- |
|  | Neuroticism | | | | | | | | | | |
| Psilocybin | | Intercept | 3.97** | .14 | 46 | 27.76 |  | .000 |  |  |  |
|  | | Time | -.54** | .14 | 29 | -3.81 | 95% [-.82, -.26] | .000 | .000 | -.70 | -.49 |
| Escitalopram | | Intercept | 4.11** | .11 | 35 | 36.62 |  | .000 |  |  |  |
|  | | Time | -.38** | .08 | 28 | -4.96 | 95% [-.53, -.23] | .000 | .000 | -.92 | -.44 |
| Interaction by Condition | | Intercept | 4.11** | .13 | 83 | 31.60 |  | .000 |  |  |  |
| Condition | | Condition | -.15 | .18 | 83 | -.80 |  | .424 |  |  |  |
|  | | Time | -.38** | .12 | 57 | -3.28 |  | .002 |  |  |  |
|  | | Condition x Time | -.16 | .16 | 57 | -.98 | 95% [-.48, .16] | .331 |  |  |  |
| Interaction by | | Intercept | 4.12** | .15 | 52 | 26.68 |  | .000 |  |  |  |
| Condition | | Condition | -.07 | .22 | 52 | -.31 |  | .756 |  |  |  |
| (matched sample) | | Time | -.43** | .11 | 40 | -3.84 |  | .000 |  |  |  |
|  | | Condition x Time | -.22 | .16 | 40 | -1.38 | 99% [-.63, .20] | .176 |  |  |  |
|  | Neuroticism (complete) | | | | | | | | | | |
| Psilocybin | | Intercept | 4.04** | .13 | 49 | 29.98 |  | .000 |  |  |  |
|  | | Time | -.63** | .14 | 29 | -4.38 | 95% [-.91, -.34] | .000 |  | -.80 | -.60 |
| Escitalopram | | Intercept | 4.18** | .10 | 37 | 39.81 |  | .000 |  |  |  |
|  | | Time | -.375** | .08 | 28 | -4.74 | 95% [-.53, -.22] | .000 |  | -.88 | -.47 |
| Interaction by Condition | | Intercept | 4.18** | .12 | 89 | 34.15 |  | .000 |  |  |  |
| Condition | | Condition | -.14 | .17 | 89 | -.81 |  | .419 |  |  |  |
|  | | Time | -.38** | .12 | 57 | -3.18 |  | .002 |  |  |  |
|  | | Condition x Time | -.25 | .17 | 57 | -1.54 | 95% [-.58, .07] | .130 |  |  |  |
| Interaction by | | Intercept | 4.17** | .15 | 54 | 28.69 |  | .000 |  |  |  |
| Condition | | Condition | -.08 | .21 | 54 | -.38 |  | .708 |  |  |  |
| (matched sample) | | Time | -.43** | .11 | 40 | -3.76 |  | .001 |  |  |  |
|  | | Condition x Time | -.27 | .16 | 40 | -1.70 | 99% [-.70, .15] | .097 |  |  |  |
|  | Extraversion (Introversion) | | | | | | | | | | |
| Psilocybin | | Intercept | 2.40** | .19 | 33 | 12.88 |  | .000 |  |  |  |
|  | | Time | .38** | .10 | 29 | 4.02 | 95% [.19, .57] | .000 | .000 | .73 | .27 |
| Escitalopram | | Intercept | 2.53** | .16 | 33 | 15.66 |  | .000 |  |  |  |
|  | | Time | .20 | .10 | 28 | 2.02 | 95% [.00, .39] | .054 | .057 | .37 | .16 |
| Interaction by Condition | | Intercept | 2.53** | .18 | 66 | 14.35 |  | .000 |  |  |  |
| Condition | | Condition | -.13 | .25 | 66 | -.53 |  | .600 |  |  |  |
|  | | Time | .20' | .10 | 57 | 2.01 |  | .049 |  |  |  |
|  | | Condition x Time | .19 | .14 | 57 | 1.40 | 95% [-.08, .45] | .168 |  |  |  |
| Interaction by | | Intercept | 2.57** | .22 | 46 | 11.90 |  | .000 |  |  |  |
| Condition | | Condition | -.12 | .31 | 46 | -.39 |  | .699 |  |  |  |
| (matched sample) | | Time | .17 | .12 | 40 | 1.45 |  | .155 |  |  |  |
|  | | Condition x Time | .20 | .16 | 40 | 1.21 | 95% [-.12, .51] | .234 |  |  |  |
|  | Openness | | | | | | | | | | |
| Psilocybin | | Intercept | 3.53** | .14 | 36 | 25.62 |  | .000 |  |  |  |
|  | | Time | .23' | .09 | 29 | 2.54 | 95% [.05, .42] | .017 | .023 | .46 | .22 |
| Escitalopram | | Intercept | 3.57** | .12 | 36 | 29.79 |  | .000 |  |  |  |
|  | | Time | .28** | .09 | 28 | 3.16 | 99% [.04, .51] | .004 | .008 | .59 | .30 |
| Interaction by Condition | | Intercept | 3.57** | .13 | 72 | 27.34 |  | .000 |  |  |  |
| Condition | | Condition | -.04 | .18 | 72 | -.21 |  | .832 |  |  |  |
|  | | Time | .28** | .09 | 57 | 3.05 |  | .003 |  |  |  |
|  | | Condition x Time | -.04 | .13 | 57 | -.34 | 95% [-.29, .21] | .738 |  |  |  |
| Interaction by | | Intercept | 3.58** | .14 | 56 | 24.81 |  | .000 |  |  |  |
| Condition | | Condition | .03 | .20 | 56 | .14 |  | .889 |  |  |  |
| (matched sample) | | Time | .26’ | .12 | 40 | 2.18 |  | .035 |  |  |  |
|  | | Condition x Time | -.03 | .17 | 40 | -.20 | 95% [-.37, .30] | .845 |  |  |  |
|  | Aesthetic Openness | | | | | | | | | | |
| Psilocybin | | Intercept | 3.72** | .13 | 40 | 27.98 |  | .000 |  |  |  |
|  | | Time | .27’ | .11 | 29 | 2.45 | 95% [.05, .48] | .021 | .027 | .45 | .26 |
| Escitalopram | | Intercept | 3.76** | .11 | 33 | 34.02 |  | .000 |  |  |  |
|  | | Time | .17’ | .07 | 28 | 2.63 | 99% [-.00, .35] | .014 | .021 | .49 | .21 |
| Interaction by Condition | | Intercept | 3.76** | .12 | 75 | 30.37 |  | .000 |  |  |  |
| Condition | | Condition | -.04 | .17 | 75 | -.20 |  | .840 |  |  |  |
|  | | Time | .17 | .09 | 57 | 1.89 |  | .064 |  |  |  |
|  | | Condition x Time | .09 | .13 | 57 | .74 | 95% [-.16, .35] | .465 |  |  |  |
| Interaction by | | Intercept | 3.82** | .14 | 56 | 27.88 |  | .000 |  |  |  |
| Condition | | Condition | -.08 | .19 | 56 | -.42 |  | .678 |  |  |  |
| (matched sample) | | Time | .13 | .12 | 40 | 1.16 |  | .254 |  |  |  |
|  | | Condition x Time | .14 | .16 | 40 | .88 | 95% [-.18, .46] | .386 |  |  |  |
|  | Intellect | | | | | | | | | | |
| Psilocybin | | Intercept | 3.41** | .14 | 37 | 24.77 |  | .000 |  |  |  |
|  | | Time | .22’ | .10 | 29 | 2.33 | 95% [.03, .41] | .027 | .032 | .43 | .21 |
| Escitalopram | | Intercept | 3.48** | .15 | 32 | 22.80 |  | .000 |  |  |  |
|  | | Time | .28** | .08 | 28 | 3.47 | 99% [.06, .50] | .002 | .005 | .64 | .24 |
| Interaction by Condition | | Intercept | 3.48** | .15 | 69 | 23.77 |  | .000 |  |  |  |
| Condition | | Condition | -.07 | .21 | 69 | -.34 |  | .737 |  |  |  |
|  | | Time | .28** | .09 | 57 | 3.15 |  | .003 |  |  |  |
|  | | Condition x Time | -.06 | .13 | 57 | -.47 | 95% [-.31, .19] | .639 |  |  |  |
| Interaction by | | Intercept | 3.50** | .18 | 49 | 19.89 |  | .000 |  |  |  |
| Condition | | Condition | -.12 | .25 | 49 | -.48 |  | .634 |  |  |  |
| (matched sample) | | Time | .22 | .11 | 40 | 2.02 |  | .050 |  |  |  |
|  | | Condition x Time | .05 | .16 | 40 | .30 | 95% [-.26, .35] | .762 |  |  |  |
|  | Absorption (without Mystical item) | | | | | | | | | | |
| Psilocybin | | Intercept | 2.18** | .15 | 32 | 14.58 |  | .000 |  |  |  |
|  | | Time | .30** | .07 | 29 | 4.26 | 99% [.11, .50] | .000 | .000 | .78 | .26 |
| Escitalopram | | Intercept | 2.02** | .11 | 35 | 19.04 |  | .000 |  |  |  |
|  | | Time | .09 | .07 | 28 | 1.30 | 99% [-.10, .28] | .205 | .205 | .24 | .11 |
| Interaction by Condition | | Intercept | 2.02** | .13 | 66 | 15.33 |  | .000 |  |  |  |
| Condition | | Condition | .17 | .18 | 66 | .90 |  | .372 |  |  |  |
|  | | Time | .09 | .07 | 57 | 1.27 |  | .209 |  |  |  |
|  | | Condition x Time | .21' | .10 | 57 | 2.14 | 99% [-.05, .47] | .037 |  |  |  |
| Interaction by | | Intercept | 2.07** | .14 | 48 | 14.60 |  | .000 |  |  |  |
| Condition | | Condition | .05 | .20 | 48 | .24 |  | .813 |  |  |  |
| (matched sample) | | Time | .05 | .08 | 40 | .59 |  | .561 |  |  |  |
|  | | Condition x Time | .32* | .12 | 40 | 2.70 | 99% [.01, .64] | .010 |  |  |  |
|  | Absorption (complete) | | | | | | | | | | |
| Psilocybin | | Intercept | 2.18** | .15 | 33 | 14.43 |  | .000 |  |  |  |
|  | | Time | .32** | .07 | 29 | 4.42 | 99% [.13, .52] | .000 |  | .81 | .28 |
| Escitalopram | | Intercept | 2.02** | .11 | 35 | 18.99 |  | .000 |  |  |  |
|  | | Time | .09 | .07 | 28 | 1.29 | 99% [-.10, .28] | .208 |  | .24 | .11 |
| Interaction by Condition | | Intercept | 2.02** | .13 | 66 | 15.20 |  | .000 |  |  |  |
| Condition | | Condition | .17 | .19 | 66 | .90 |  | .219 |  |  |  |
|  | | Time | .09 | .07 | 57 | 1.24 |  | .371 |  |  |  |
|  | | Condition x Time | .23' | .10 | 57 | 2.32 | 99% [-.03, .50] | .024 |  |  |  |
| Interaction by | | Intercept | 2.07** | .14 | 48 | 14.43 |  | .000 |  |  |  |
| Condition | | Condition | .05 | .20 | 48 | .25 |  | .801 |  |  |  |
| (matched sample) | | Time | .05 | .09 | 40 | .62 |  | .536 |  |  |  |
|  | | Condition x Time | .34* | .12 | 40 | 2.77 | 99% [.02, .65] | .008 |  |  |  |
|  | Agreeableness (Disagreeableness) | | | | | | | | | | |
| Psilocybin | | Intercept | 3.21** | .14 | 39 | 23.58 |  | .000 |  |  |  |
|  | | Time | .47** | .11 | 29 | 4.41 | 95% [.26, .69] | .000 | .000 | .81 | .45 |
| Escitalopram | | Intercept | 3.18** | .10 | 41 | 30.40 |  | .000 |  |  |  |
|  | | Time | .26* | .09 | 28 | 2.86 | 95% [.08, .44] | .008 | .014 | .53 | .33 |
| Interaction by Condition | | Intercept | 3.18** | .12 | 79 | 25.83 |  | .000 |  |  |  |
| Condition | | Condition | .04 | .17 | 79 | .20 |  | .840 |  |  |  |
|  | | Time | .26' | .10 | 57 | 2.61 |  | .012 |  |  |  |
|  | | Condition x Time | .21 | .14 | 57 | 1.48 | 99% [-.16, 58] | .146 |  |  |  |
| Interaction by | | Intercept | 3.29** | .15 | 59 | 22.50 |  | .000 |  |  |  |
| Condition | | Condition | -.16 | .21 | 59 | -.79 |  | .430 |  |  |  |
| (matched sample) | | Time | .22 | .13 | 40 | 1.71 |  | .095 |  |  |  |
|  | | Condition x Time | .28 | .18 | 40 | 1.53 | 99% [-.20, .76] | .135 |  |  |  |
|  | Conscientiousness | | | | | | | | | | |
| Psilocybin | | Intercept | 3.10** | .13 | 42 | 23.99 |  | .000 |  |  |  |
|  | | Time | .30' | .11 | 29 | 2.68 | 99% [-.00, .60] | .012 | .020 | .49 | .30 |
| Escitalopram | | Intercept | 2.93** | .15 | 34 | 18.93 |  | .000 |  |  |  |
|  | | Time | .22' | .09 | 28 | 2.32 | 99% [-.03, .47] | .028 | .032 | .43 | .19 |
| Interaction by Condition | | Intercept | 2.93** | .14 | 74 | 20.42 |  | .000 |  |  |  |
| Condition | | Condition | .18 | .20 | 74 | .88 |  | .383 |  |  |  |
|  | | Time | .22' | .10 | 57 | 2.09 |  | .042 |  |  |  |
|  | | Condition x Time | .08 | .15 | 57 | .56 | 99% [-.30, .46] | .581 |  |  |  |
| Interaction by | | Intercept | 3.03** | .18 | 53 | 16.52 |  | .000 |  |  |  |
| Condition | | Condition | .08 | .26 | 53 | .31 |  | .761 |  |  |  |
| (matched sample) | | Time | .23 | .14 | 40 | 1.71 |  | .096 |  |  |  |
|  | | Condition x Time | .12 | .19 | 40 | .60 | 99% [-.39, .62] | .550 |  |  |  |
|  | Impulsivity | | | | | | | | | | |
| Psilocybin | | Intercept | 3.02** | .07 | 39 | 44.46 |  | .000 |  |  |  |
|  | | Time | -.40** | .05 | 29 | -7.60 | 95% [-.30, -.51] | .000 | .000 | -1.39 | -.77 |
| Escitalopram | | Intercept | 3.06** | .09 | 36 | 32.67 |  | .000 |  |  |  |
|  | | Time | -.35** | .07 | 28 | -5.31 | 95% [-.22, -.48] | .000 | .000 | -.99 | -.49 |
| Interaction by Condition | | Intercept | 3.06** | .08 | 74 | 37.26 |  | .000 |  |  |  |
| Condition | | Condition | -.04 | .12 | 74 | -.31 |  | .760 |  |  |  |
|  | | Time | -.35** | .06 | 57 | -5.81 |  | .000 |  |  |  |
|  | | Condition x Time | -.06 | .08 | 57 | -.65 | 99% [.16, -.27] | .516 |  |  |  |
| Interaction by | | Intercept | 3.02** | .09 | 54 | 31.97 |  | .000 |  |  |  |
| Condition | | Condition | .08 | .13 | 54 | .62 |  | .535 |  |  |  |
| (matched sample) | | Time | -.37** | .07 | 40 | -5.05 |  | .000 |  |  |  |
|  | | Condition x Time | -.04 | .10 | 40 | -.40 | 99% [-.31, .23] | .689 |  |  |  |
| *Note.* Unstandardized (B) coefficients indicate mean differences between timepoints; df = degrees of freedom; *dz* indicates effect size change in outcome scores in terms of the standard deviation of within-subject change scores (e.g., T2-T1; Lakens, 2013). Cohen’s *ds* (standard Cohen’s d; Cohen, 1988) effect size estimates were calculated using the following equation: (Mean-score T2-Mean-score T1)/((SDT1)2+SDT2)2)0.5. Neuroticism = Big Five Inventory (BFI) Neuroticism without Item 4; Neuroticism (complete) = BFI Neuroticism; Extraversion = BFI Extraversion; Openness = BFI Openness; Intellect = Big Five Aspects Scale (BFAS) Intellect; Aesthetic Openness = BFAS Openness; Absorption (without mystical) = Modified-Tellegen Absorption Scale (MODTAS) Absorption without MODTAS Item 5; Absorption (complete) = MODTAS Absorption; Agreeableness = BFI Agreeableness; Conscientiousness = BFI Conscientiousness; Impulsivity = Barrett Impulsivity Inventory-Brief mean-score. Analyses for which a 95% confidence interval is presented were hypothesized. FDR = Benjamini & Hochberg’s False Discovery Rate adjustment. ‘p<.05, **p*<.01, ***p*<.005. | | | | | | | | | | | |
|  | |  |  |  |  |  |  |  |  |  |  |

Supplementary Table 2

Omnibus ANOVA tests - Examining within-condition changes in personality between Baseline, Week 6, and Month 6 (dataset B)

| Condition/Outcome | Sum of Squares | Mean Squares | Num df | Den df | F-value | p-value | FDR adjusted  p-value |
| --- | --- | --- | --- | --- | --- | --- | --- |
| Neuroticism |  |  |  |  |  |  |  |
| Psilocybin | 5.08 | 2.54 | 2 | 48 | 6.99 | .002 | .009 |
| Escitalopram | 2.42 | 1.21 | 2 | 40 | 8.93 | .001 | .007 |
| Extraversion (Introversion) | |  |  |  |  |  |  |
| Psilocybin | 2.01 | 1.00 | 2 | 48 | 6.80 | .003 | .011 |
| Escitalopram | .406 | .203 | 2 | 40 | 1.81 | .177 | .223 |
| Openness |  |  |  |  |  |  |  |
| Psilocybin | .78 | .39 | 2 | 48 | 2.73 | .076 | .158 |
| Escitalopram | .28 | .14 | 2 | 40 | 1.73 | .191 | .223 |
| Aesthetic Openness | |  |  |  |  |  |  |
| Psilocybin | .58 | .29 | 2 | 48 | 1.71 | .191 | .223 |
| Escitalopram | .24 | .12 | 2 | 40 | 1.61 | .212 | .228 |
| Intellect |  |  |  |  |  |  |  |
| Psilocybin | 1.73 | .87 | 2 | 48 | 2.33 | .108 | .189 |
| Escitalopram | .96 | .48 | 2 | 40 | 1.44 | .250 | .250 |
| Agreeableness (Disagreeableness) | |  |  |  |  |  |  |
| Psilocybin | 3.53 | 1.76 | 2 | 48 | 8.46 | .001 | .007 |
| Escitalopram | .77 | .38 | 2 | 40 | 3.60 | .037 | .104 |
| Conscientiousness | |  |  |  |  |  |  |
| Psilocybin | 1.04 | .52 | 2 | 48 | 2.18 | .124 | .193 |
| Escitalopram | .77 | .38 | 2 | 40 | 2.70 | .079 | .158 |
| *Note.* Neuroticism = Big Five Inventory (BFI) Neuroticism without Item 4; Extraversion = BFI Extraversion; Openness = BFI Openness; Intellect = Big Five Aspects Scale (BFAS) Intellect; Aesthetic Openness = BFAS Openness; Agreeableness = BFI Agreeableness; Conscientiousness = BFI Conscientiousness; FDR = Benjamini & Hochberg’s False Discovery Rate adjustment. | | | | | | | |

Supplementary Table 3

Examining within-condition changes in personality between Baseline, Week 6, and Month 6 (dataset B)

| Condition | Parameter | B | Std Error | df | t-value | Confidence Interval | p-value | *dz* | *ds* |
| --- | --- | --- | --- | --- | --- | --- | --- | --- | --- |
| Neuroticism | | | | | | | | | |
| Psilocybin | Intercept | 4.06** | .16 | 51 | 24.71 |  | .000 |  |  |
|  | Time Week 6 | -.61** | .17 | 48 | -3.56 | -1.05, -.16 | .001 | -.67 | -.52 |
|  | Time Month 6 | -.47* | .17 | 48 | -2.78 | -.92, -.03 | .008 | -.57 | -.42 |
| Escitalopram | Intercept | 4.07** | .14 | 31 | 28.43 |  | .000 |  |  |
|  | Time Week 6 | -.35** | .11 | 40 | -3.07 | -.65, -.05 | .004 | -.90 | -.37 |
|  | Time Month 6 | -.46** | .11 | 40 | -4.05 | -.76, -.16 | .000 | -.89 | -.51 |
| Extraversion (Introversion) | | | | | | | | | |
| Psilocybin | Intercept | 2.54** | .21 | 29 | 11.88 |  | .000 |  |  |
|  | Time Week 6 | .40** | .11 | 48 | 3.68 | .12, .68 | .001 | .76 | .28 |
|  | Time Month 6 | .18 | .11 | 48 | 1.66 | -.10, .46 | .104 | .34 | .11 |
| Agreeableness (Disagreeableness) | | | | | | | | | |
| Psilocybin | Intercept | 3.22** | .16 | 37 | 19.97 |  | .000 |  |  |
|  | Time Week 6 | .50** | .13 | 48 | 3.87 | .16, .84 | .000 | .73 | .44 |
|  | Time Month 6 | .41** | .13 | 48 | 3.14 | .07, .74 | .003 | .67 | .35 |
| *Note.* Unstandardized (B) coefficients indicate mean differences between baseline and other timepoint; df = degrees of freedom; *dz* indicates effect size change in outcome scores in terms of the standard deviation of within-subject change scores (e.g., T2-T1; Lakens, 2013). Cohen’s *ds* (standard Cohen’s d; Cohen, 1988) effect size estimates were calculated using the following equation: (Mean-score T2-Mean-score T1)/((SDT1)2+SDT2)2)0.5. Neuroticism = Big Five Inventory (BFI) Neuroticism without Item 4; Extraversion = BFI Extraversion; Openness = BFI Openness; Intellect = Big Five Aspects Scale (BFAS) Intellect; Aesthetic Openness = BFAS Openness; Agreeableness = BFI Agreeableness; Conscientiousness = BFI Conscientiousness. ‘p<.05, **p*<.01, ***p*<.005. | | | | | | | | | |

Supplementary Table 4

Omnibus ANOVA tests - Examining between-condition changes in personality between Baseline,

Week 6, and Month 6 (dataset B)

| Outcome/Parameter | Sum of Squares | Mean of Squares | Num df | Den df | F-value | p-value |
| --- | --- | --- | --- | --- | --- | --- |
| Neuroticism |  |  |  |  |  |  |
| Time | 2.42 | 1.21 | 2 | 88 | 4.66 | .012 |
| Condition | .08 | .08 | 1 | 44 | .30 | .586 |
| Time x Condition | .48 | .24 | 2 | 88 | .92 | .401 |
| Extraversion (Introversion) | |  |  |  |  |  |
| Time | .41 | .20 | 2 | 88 | 1.54 | .219 |
| Condition | .00 | .00 | 1 | 44 | .00 | .996 |
| Time x Condition | .69 | .35 | 2 | 88 | 2.63 | .078 |
| Openness |  |  |  |  |  |  |
| Time | .28 | .14 | 2 | 88 | 1.23 | .297 |
| Condition | .06 | .06 | 1 | 44 | .49 | .490 |
| Time x Condition | .10 | .05 | 2 | 88 | .43 | .650 |
| Aesthetic Openness | |  |  |  |  |  |
| Time | .24 | .12 | 2 | 88 | .95 | .390 |
| Condition | .02 | .02 | 1 | 44 | .18 | .676 |
| Time x Condition | .02 | .01 | 2 | 88 | .10 | .909 |
| Intellect |  |  |  |  |  |  |
| Time | .96 | .48 | 2 | 88 | 1.35 | .264 |
| Condition | .55 | .55 | 1 | 44 | 1.56 | .219 |
| Time x Condition | .21 | .10 | 2 | 88 | .29 | .749 |
| Agreeableness (Disagreeableness) | |  |  |  |  |  |
| Time | .77 | .38 | 2 | 88 | 2.36 | .100 |
| Condition | .11 | .11 | 1 | 44 | .68 | .414 |
| Time x Condition | .39 | .19 | 2 | 88 | 1.20 | .306 |
| Conscientiousness | |  |  |  |  |  |
| Time | .77 | .38 | 2 | 88 | 1.97 | .146 |
| Condition | .162 | .162 | 1 | 44 | .83 | .366 |
| Time x Condition | .08 | .04 | 2 | 88 | .22 | .807 |
| *Note.* Neuroticism = Big Five Inventory (BFI) Neuroticism without Item 4; Extraversion = BFI Extraversion; Openness = BFI Openness; Intellect = Big Five Aspects Scale (BFAS) Intellect; Aesthetic Openness = BFAS Openness; Agreeableness = BFI Agreeableness; Conscientiousness = BFI Conscientiousness. | | | | | | |

Supplementary Table 5

Examining moderation of within-condition change by expectancy (dataset A)

| Condition/Outcome | Parameter | B | SE (B) | df | t-value | p-value |
| --- | --- | --- | --- | --- | --- | --- |
| **Escitalopram Treatment** | |  |  |  |  |  |
| Neuroticism | Intercept | 4.18** | .19 | 31 | 21.64 | .000 |
|  | Time | -.05 | .11 | 26 | -.40 | .690 |
|  | Escitalopram expectancy | .00 | .01 | 31 | -.47 | .641 |
|  | Time x Escitalopram expectancy | -.01** | .00 | 26 | -3.44 | .002 |
| Extraversion | Intercept | 2.22** | .38 | 51 | 5.81 | .000 |
| (Introversion) | Time | .18 | .18 | 26 | 1.02 | .320 |
|  | Escitalopram expectancy | .00 | .01 | 51 | .30 | .77 |
|  | Time x Escitalopram expectancy | .00 | .00 | 26 | .09 | .930 |
| Openness | Intercept | 3.59** | .22 | 33 | 16.60 | .000 |
|  | Time | .06 | .15 | 26 | .40 | .694 |
|  | Escitalopram expectancy | .00 | .01 | 33 | -.12 | .909 |
|  | Time x Escitalopram expectancy | .01 | .00 | 26 | 1.75 | .092 |
| Aesthetic Openness | Intercept | 3.87** | .26 | 51 | 15.25 | .000 |
|  | Time | .02 | .12 | 26 | .15 | .882 |
|  | Escitalopram expectancy | -.01 | .02 | 51 | -1.25 | .216 |
|  | Time x Escitalopram expectancy | .00 | .00 | 26 | 1.60 | .123 |
| Intellect | Intercept | 3.06** | .26 | 30 | 11.93 | .000 |
|  | Time | .29 | .14 | 26 | 2.01 | .055 |
|  | Escitalopram expectancy | .01 | .01 | 30 | 1.80 | .082 |
|  | Time x Escitalopram expectancy | .00 | .00 | 26 | .13 | .900 |
| Absorption | Intercept | 1.81** | .27 | 51 | 6.87 | .000 |
|  | Time | .15 | .12 | 26 | 1.15 | .260 |
|  | Escitalopram expectancy | .00 | .01 | 51 | .54 | .592 |
|  | Time x Escitalopram expectancy | .00 | .00 | 26 | -.52 | .608 |
| Agreeableness | Intercept | 3.02** | .18 | 38 | 16.67 | .000 |
| (Disagreeableness) | Time | .12 | .16 | 26 | .73 | .470 |
|  | Escitalopram expectancy | .01 | .00 | 38 | 1.04 | .305 |
|  | Time x Escitalopram expectancy | .01 | .00 | 26 | 1.19 | .244 |
| Conscientiousness | Intercept | 3.22** | .34 | 50 | 9.52 | .000 |
|  | Time | -.15 | .15 | 26 | -1.04 | .307 |
|  | Escitalopram expectancy | -.02’ | .01 | 50 | -2.02 | .048 |
|  | Time x Escitalopram expectancy | .01** | .00 | 26 | 3.14 | .004 |
| Impulsivity | Intercept | 3.13** | .16 | 34 | 19.75 | .000 |
|  | Time | -.33’ | .12 | 26 | -2.75 | .011 |
|  | Escitalopram expectancy | .00 | .00 | 34 | -.37 | .715 |
|  | Time x Escitalopram expectancy | .00 | .00 | 26 | -.13 | .896 |
| **Psilocybin Therapy** | |  |  |  |  |  |
| Neuroticism | Intercept | 3.29** | .43 | 40 | 7.62 | .000 |
|  | Time | -.46 | .43 | 25 | -1.07 | .294 |
|  | Psilocybin expectancy | .01 | .01 | 40 | 1.60 | .117 |
|  | Time x Psilocybin expectancy | .00 | .01 | 25 | -.21 | .832 |
| Extraversion | Intercept | 1.80** | .54 | 29 | 3.34 | .002 |
| (Introversion) | Time | .35 | .29 | 25 | 1.21 | .238 |
|  | Psilocybin expectancy | .01 | .01 | 29 | 1.15 | .260 |
|  | Time x Psilocybin expectancy | .00 | .00 | 25 | .12 | .905 |
| Openness | Intercept | 3.02** | .42 | 31 | 7.13 | .000 |
|  | Time | .23 | .29 | 25 | .80 | .433 |
|  | Psilocybin expectancy | .01 | .01 | 31 | 1.30 | .203 |
|  | Time x Psilocybin expectancy | .00 | .00 | 25 | -.03 | .979 |
| Intellect | Intercept | 3.03** | .43 | 32 | 7.08 | .000 |
|  | Time | .23 | .31 | 25 | .75 | .461 |
|  | Psilocybin expectancy | .01 | .01 | 32 | .84 | .409 |
|  | Time x Psilocybin expectancy | .00 | .01 | 25 | -.07 | .948 |
| Aesthetic Openness | Intercept | 3.13** | .40 | 36 | 7.81 | .000 |
|  | Time | .15 | .35 | 25 | .43 | .671 |
|  | Psilocybin expectancy | .01 | .01 | 36 | 1.51 | .139 |
|  | Time x Psilocybin expectancy | .00 | .01 | 25 | .37 | .715 |
| Absorption | Intercept | 2.22** | .47 | 28 | 4.76 | .000 |
|  | Time | .17 | .22 | 25 | .77 | .450 |
|  | Psilocybin expectancy | .00 | .01 | 28 | -.02 | .981 |
|  | Time x Psilocybin expectancy | .00 | .00 | 25 | .53 | .600 |
| Agreeableness | Intercept | 3.24** | .41 | 34 | 7.85 | .000 |
| (Disagreeableness) | Time | .55 | .33 | 25 | 1.66 | .110 |
|  | Psilocybin expectancy | .00 | .01 | 34 | .11 | .917 |
|  | Time x Psilocybin expectancy | .00 | .01 | 25 | -.25 | .805 |
| Impulsivity | Intercept | 2.83** | .20 | 33 | 13.82 | .000 |
|  | Time | -.37’ | .16 | 25 | -2.35 | .027 |
|  | Psilocybin expectancy | .00 | .00 | 33 | .94 | .353 |
|  | Time x Psilocybin expectancy | .00 | .00 | 25 | -.28 | .780 |
| *Note.* B = unstandardized coefficient; All variables are unstandardized; df = degrees of freedom. Neuroticism = Big Five Inventory (BFI) Neuroticism without Item 4; Extraversion = BFI Extraversion; Openness = BFI Openness; Intellect = Big Five Aspects Scale (BFAS) Intellect; Aesthetic Openness = BFAS Openness; Absorption (without mystical) = Modified-Tellegen Absorption Scale (MODTAS) Absorption without MODTAS Item 5; Agreeableness = BFI Agreeableness; Impulsivity = Barrett Impulsivity Inventory-Brief mean-score. ‘p<.05, **p*<.01, ***p*<.005. | | | | | | |

Supplementary Table 6

Examining moderation by baseline characteristics, personality, and acute factors

| Outcome/Moderator | Parameters | B | SE (B) | Df | t-value | p-value |
| --- | --- | --- | --- | --- | --- | --- |
| **Escitalopram Treatment** | | | | | | |
| Neuroticism | | | | | | |
| Age | Intercept | 4.14 | .48 | 33.90 | 8.60 | .000 |
|  | Age | .00 | .01 | 33.90 | -.05 | .962 |
|  | Time | -.53 | .33 | 27.00 | -1.64 | .113 |
|  | Time x Age | .00 | .01 | 27.00 | .49 | .630 |
| BFI Agreeableness | Intercept | 4.82 | .63 | 34.38 | 7.69 | .000 |
|  | BFI A | -.22 | .19 | 34.38 | -1.15 | .257 |
|  | Time | -.12 | .44 | 27.00 | -.28 | .782 |
|  | Time x BFI A | -.08 | .14 | 27.00 | -.60 | .557 |
| BFI Conscientiousness | Intercept | 4.33 | .42 | 34.05 | 10.42 | .000 |
|  | BFI C | -.07 | .14 | 34.05 | -.55 | .589 |
|  | Time | -.28 | .28 | 27.00 | -.98 | .337 |
|  | Time x BFI C | -.03 | .09 | 27.00 | -.37 | .715 |
| Emotional Breakthrough | Intercept | 4.18 | .17 | 33.99 | 24.76 | .000 |
|  | EBI | .00 | .00 | 33.99 | -.53 | .601 |
|  | Time | -.32 | .12 | 27.00 | -2.76 | .010 |
|  | Time x EBI | .00 | .00 | 27.00 | -.71 | .482 |
| Education level | Intercept | 4.44 | .50 | 33.95 | 8.84 | .000 |
|  | EduLevel | -.08 | .12 | 33.95 | -.66 | .514 |
|  | Time | -.02 | .34 | 27.00 | -.04 | .965 |
|  | Time x EduLevel | -.09 | .08 | 27.00 | -1.09 | .284 |
| Emotional insight | Intercept | 4.15 | .17 | 33.98 | 24.94 | .000 |
|  | Emo Insight | .00 | .00 | 33.98 | -.33 | .747 |
|  | Time | -.35 | .11 | 27.00 | -3.09 | .005 |
|  | Time x Emo Insight | .00 | .00 | 27.00 | -.35 | .732 |
| BFI Extraversion | Intercept | 4.85 | .31 | 35.94 | 15.53 | .000 |
|  | BFI E | -.29 | .12 | 35.94 | -2.48 | .018 |
|  | Time | -.07 | .24 | 27.00 | -.30 | .766 |
|  | Time x BFI E | -.12 | .09 | 27.00 | -1.36 | .185 |
| Sex | Intercept | 4.13 | .14 | 33.74 | 30.24 | .000 |
|  | Sex | -.05 | .25 | 33.74 | -.20 | .842 |
|  | Time | -.32 | .09 | 27.00 | -3.51 | .002 |
|  | Time x Sex | -.19 | .16 | 27.00 | -1.13 | .267 |
| MEQ Ineffable | Intercept | 4.23 | .17 | 34.15 | 24.88 | .000 |
|  | MEQ Ineffable | -.08 | .09 | 34.15 | -.88 | .383 |
|  | Time | -.30 | .12 | 27.00 | -2.58 | .015 |
|  | Time x MEQ Ineffable | -.06 | .07 | 27.00 | -.87 | .394 |
| Intensity | Intercept | 4.19 | .16 | 34.09 | 25.65 | .000 |
|  | Intensity | .00 | .00 | 34.09 | -.65 | .521 |
|  | Time | -.35 | .11 | 27.00 | -3.12 | .004 |
|  | Time x Intensity | .00 | .00 | 27.00 | -.37 | .716 |
| MEQ Mystical | Intercept | 4.21 | .19 | 34.09 | 22.55 | .000 |
|  | MEQ Mystical | -.06 | .09 | 34.09 | -.65 | .520 |
|  | Time | -.34 | .13 | 27.00 | -2.64 | .013 |
|  | Time x MEQ Mystical | -.03 | .06 | 27.00 | -.39 | .696 |
| BFI Neuroticism | Intercept | .00 | .39 | 54.00 | .00 | 1.000 |
|  | BFI N | 1.00 | .10 | 54.00 | 10.52 | .000 |
|  | Time | .24 | .56 | 54.00 | .44 | .664 |
|  | Time x BFI N | -.15 | .13 | 54.00 | -1.13 | .265 |
| BFI Openness | Intercept | 4.27 | .64 | 33.97 | 6.67 | .000 |
|  | BFI O | -.04 | .18 | 33.97 | -.24 | .809 |
|  | Time | -.37 | .44 | 27.00 | -.84 | .408 |
|  | Time x BFI O | .00 | .12 | 27.00 | -.03 | .975 |
| MEQ Positive mood | Intercept | 4.16 | .18 | 33.97 | 22.88 | .000 |
|  | MEQ Positive mood | -.03 | .09 | 33.97 | -.35 | .731 |
|  | Time | -.34 | .12 | 27.00 | -2.72 | .011 |
|  | Time x MEQ Positive mood | -.03 | .06 | 27.00 | -.43 | .671 |
| Prev Psychedelic Use | Intercept | 4.18 | .13 | 33.77 | 31.32 | .000 |
|  | PrevPsychUse | -.23 | .25 | 33.77 | -.91 | .371 |
|  | Time | -.43 | .09 | 27.00 | -4.78 | .000 |
|  | Time x PrevPsychUse | .18 | .17 | 27.00 | 1.05 | .305 |
| BFAS Intellect | Intercept | 4.92 | .44 | 34.86 | 11.19 | .000 |
|  | BFAS I | -.23 | .12 | 34.86 | -1.89 | .067 |
|  | Time | .18 | .32 | 27.00 | .58 | .568 |
|  | Time x BFAS I | -.16 | .09 | 27.00 | -1.83 | .079 |
| BFAS Openness | Intercept | 3.55 | .69 | 34.17 | 5.11 | .000 |
|  | BFAS O | .15 | .18 | 34.17 | .82 | .419 |
|  | Time | -.52 | .48 | 27.00 | -1.08 | .290 |
|  | Time x BFAS O | .04 | .13 | 27.00 | .29 | .772 |
| BIS-B Impulsivity | Intercept | 4.75 | .60 | 33.32 | 7.87 | .000 |
|  | BIS-B | -.23 | .21 | 33.32 | -1.07 | .291 |
|  | Time | -1.06 | .39 | 27.00 | -2.70 | .012 |
|  | Time x BIS-B | .25 | .14 | 27.00 | 1.77 | .089 |
| MEQ Time-space | Intercept | 4.15 | .18 | 33.96 | 23.59 | .000 |
|  | MEQ Time-space | -.02 | .09 | 33.96 | -.24 | .809 |
|  | Time | -.35 | .12 | 27.00 | -2.91 | .007 |
|  | Time x MEQ Time-space | -.02 | .06 | 27.00 | -.35 | .733 |
| Unemployed | Intercept | 4.03 | .12 | 34.70 | 33.59 | .000 |
|  | Unemployed | .48 | .29 | 34.70 | 1.68 | .102 |
|  | Time | -.37 | .09 | 27.00 | -4.38 | .000 |
|  | Time x Unemployed | -.03 | .21 | 27.00 | -.12 | .904 |
| Extraversion | | | | | | |
| Age | Intercept | 2.67 | .69 | 32.23 | 3.87 | .001 |
|  | Age | .00 | .02 | 32.23 | -.21 | .833 |
|  | Time | .18 | .41 | 27.00 | .45 | .657 |
|  | Time x Age | .00 | .01 | 27.00 | .02 | .982 |
| BFI Agreeableness | Intercept | 1.81 | .92 | 32.22 | 1.96 | .059 |
|  | BFI A | .23 | .29 | 32.22 | .79 | .437 |
|  | Time | .54 | .55 | 27.00 | .98 | .337 |
|  | Time x BFI A | -.11 | .17 | 27.00 | -.63 | .531 |
| BFI Conscientiousness | Intercept | 2.92 | .60 | 32.04 | 4.88 | .000 |
|  | BFI C | -.14 | .20 | 32.04 | -.69 | .496 |
|  | Time | -.17 | .35 | 27.00 | -.49 | .630 |
|  | Time x BFI C | .12 | .12 | 27.00 | 1.08 | .290 |
| Emotional Breakthrough | Intercept | 2.49 | .24 | 32.22 | 10.18 | .000 |
|  | EBI | .00 | .01 | 32.22 | .17 | .862 |
|  | Time | .16 | .15 | 27.00 | 1.12 | .271 |
|  | Time x EBI | .00 | .00 | 27.00 | .27 | .786 |
| Education level | Intercept | 1.53 | .71 | 32.61 | 2.14 | .040 |
|  | EduLevel | .24 | .16 | 32.61 | 1.44 | .159 |
|  | Time | .29 | .44 | 27.00 | .66 | .517 |
|  | Time x EduLevel | -.02 | .10 | 27.00 | -.22 | .827 |
| Emotional insight | Intercept | 2.45 | .24 | 32.12 | 10.22 | .000 |
|  | Emo Insight | .00 | .01 | 32.12 | .44 | .666 |
|  | Time | .28 | .14 | 27.00 | 1.95 | .062 |
|  | Time x Emo Insight | .00 | .00 | 27.00 | -.79 | .435 |
| BFI Extraversion | Intercept | .00 | .21 | 54.00 | .00 | 1.000 |
|  | BFI E | 1.00 | .08 | 54.00 | 12.56 | .000 |
|  | Time | .58 | .30 | 54.00 | 1.95 | .056 |
|  | Time x BFI E | -.15 | .11 | 54.00 | -1.37 | .175 |
| Sex | Intercept | 2.42 | .19 | 32.06 | 12.86 | .000 |
|  | Sex | .35 | .34 | 32.06 | 1.02 | .314 |
|  | Time | .08 | .11 | 27.00 | .68 | .503 |
|  | Time x Sex | .38 | .20 | 27.00 | 1.93 | .064 |
| MEQ Ineffable | Intercept | 2.51 | .25 | 32.15 | 10.04 | .000 |
|  | MEQ Ineffable | .01 | .14 | 32.15 | .07 | .944 |
|  | Time | .27 | .15 | 27.00 | 1.80 | .083 |
|  | Time x MEQ Ineffable | -.05 | .08 | 27.00 | -.65 | .519 |
| Intensity | Intercept | 2.60 | .24 | 32.24 | 11.06 | .000 |
|  | Intensity | .00 | .01 | 32.24 | -.46 | .650 |
|  | Time | .25 | .14 | 27.00 | 1.81 | .081 |
|  | Time x Intensity | .00 | .00 | 27.00 | -.60 | .555 |
| MEQ Mystical | Intercept | 2.49 | .27 | 32.02 | 9.20 | .000 |
|  | MEQ Mystical | .02 | .14 | 32.02 | .17 | .865 |
|  | Time | .33 | .16 | 27.00 | 2.07 | .048 |
|  | Time x MEQ Mystical | -.09 | .08 | 27.00 | -1.07 | .295 |
| BFI Neuroticism | Intercept | 5.18 | 1.11 | 33.04 | 4.65 | .000 |
|  | BFI N | -.64 | .27 | 33.04 | -2.40 | .022 |
|  | Time | -.39 | .71 | 27.00 | -.54 | .591 |
|  | Time x BFI N | .14 | .17 | 27.00 | .82 | .417 |
| BFI Openness | Intercept | .32 | .85 | 32.09 | .38 | .707 |
|  | BFI O | .62 | .24 | 32.09 | 2.62 | .013 |
|  | Time | 1.31 | .50 | 27.00 | 2.60 | .015 |
|  | Time x BFI O | -.31* | .14 | 27.00 | -2.25 | .033 |
| MEQ Positive mood | Intercept | 2.59 | .26 | 32.21 | 9.92 | .000 |
|  | MEQ Positive mood | -.04 | .13 | 32.21 | -.32 | .751 |
|  | Time | .27 | .16 | 27.00 | 1.72 | .097 |
|  | Time x MEQ Positive mood | -.05 | .08 | 27.00 | -.61 | .548 |
| Prev Psychedelic Use | Intercept | 2.36 | .19 | 32.50 | 12.70 | .000 |
|  | PrevPsychUse | .59 | .35 | 32.50 | 1.67 | .105 |
|  | Time | .24 | .11 | 27.00 | 2.15 | .041 |
|  | Time x PrevPsychUse | -.18 | .22 | 27.00 | -.84 | .409 |
| BFAS Intellect | Intercept | 1.36 | .68 | 32.19 | 1.99 | .055 |
|  | BFAS I | .34 | .19 | 32.19 | 1.75 | .089 |
|  | Time | .77 | .41 | 27.00 | 1.88 | .071 |
|  | Time x BFAS I | -.16 | .11 | 27.00 | -1.45 | .160 |
| BFAS Openness | Intercept | 1.77 | 1.01 | 32.18 | 1.76 | .088 |
|  | BFAS O | .20 | .26 | 32.18 | .76 | .452 |
|  | Time | .63 | .60 | 27.00 | 1.06 | .301 |
|  | Time x BFAS O | -.12 | .16 | 27.00 | -.74 | .465 |
| BIS-B Impulsivity | Intercept | 1.59 | .86 | 32.31 | 1.84 | .075 |
|  | BIS-B | .34 | .31 | 32.31 | 1.11 | .277 |
|  | Time | .54 | .52 | 27.00 | 1.04 | .309 |
|  | Time x BIS-B | -.12 | .18 | 27.00 | -.67 | .506 |
| MEQ Time-space | Intercept | 2.57 | .25 | 32.19 | 10.19 | .000 |
|  | MEQ Time-space | -.03 | .13 | 32.19 | -.22 | .828 |
|  | Time | .26 | .15 | 27.00 | 1.75 | .092 |
|  | Time x MEQ Time-space | -.05 | .08 | 27.00 | -.60 | .555 |
| Unemployed | Intercept | 2.63 | .17 | 32.63 | 15.08 | .000 |
|  | Unemployed | -.61 | .42 | 32.63 | -1.44 | .159 |
|  | Time | .19 | .11 | 27.00 | 1.74 | .093 |
|  | Time x Unemployed | .04 | .26 | 27.00 | .14 | .886 |
| Openness | | | | | | |
| Age | Intercept | 3.72 | .51 | 35.02 | 7.26 | .000 |
|  | Age | .00 | .01 | 35.02 | -.31 | .762 |
|  | Time | .15 | .37 | 27.00 | .41 | .685 |
|  | Time x Age | .00 | .01 | 27.00 | .34 | .737 |
| BFI Agreeableness | Intercept | 3.47 | .69 | 34.96 | 5.04 | .000 |
|  | BFI A | .03 | .21 | 34.96 | .15 | .883 |
|  | Time | -.06 | .50 | 27.00 | -.13 | .901 |
|  | Time x BFI A | .11 | .15 | 27.00 | .69 | .497 |
| BFI Conscientiousness | Intercept | 4.14 | .44 | 35.33 | 9.46 | .000 |
|  | BFI C | -.19 | .14 | 35.33 | -1.34 | .189 |
|  | Time | .08 | .32 | 27.00 | .25 | .803 |
|  | Time x BFI C | .07 | .11 | 27.00 | .63 | .537 |
| Emotional Breakthrough | Intercept | 3.60 | .18 | 35.03 | 19.78 | .000 |
|  | EBI | .00 | .00 | 35.03 | -.24 | .811 |
|  | Time | .25 | .13 | 27.00 | 1.90 | .068 |
|  | Time x EBI | .00 | .00 | 27.00 | .24 | .811 |
| Education level | Intercept | 3.64 | .54 | 34.31 | 6.72 | .000 |
|  | EduLevel | -.01 | .12 | 34.31 | -.12 | .905 |
|  | Time | -.37 | .38 | 27.00 | -.99 | .333 |
|  | Time x EduLevel | .15 | .09 | 27.00 | 1.76 | .089 |
| Emotional insight | Intercept | 3.57 | .18 | 34.99 | 20.02 | .000 |
|  | Emo Insight | .00 | .00 | 34.99 | .04 | .972 |
|  | Time | .23 | .13 | 27.00 | 1.79 | .085 |
|  | Time x Emo Insight | .00 | .00 | 27.00 | .47 | .641 |
| BFI Extraversion | Intercept | 2.65 | .35 | 36.77 | 7.59 | .000 |
|  | BFI E | .37 | .13 | 36.77 | 2.80 | .008 |
|  | Time | .51 | .28 | 27.00 | 1.83 | .079 |
|  | Time x BFI E | -.09 | .10 | 27.00 | -.87 | .389 |
| Sex | Intercept | 3.60 | .15 | 34.91 | 24.51 | .000 |
|  | Sex | -.09 | .26 | 34.91 | -.34 | .738 |
|  | Time | .24 | .11 | 27.00 | 2.22 | .035 |
|  | Time x Sex | .13 | .19 | 27.00 | .69 | .495 |
| MEQ Ineffable | Intercept | 3.58 | .19 | 34.99 | 19.25 | .000 |
|  | MEQ Ineffable | -.01 | .10 | 34.99 | -.08 | .940 |
|  | Time | .23 | .14 | 27.00 | 1.74 | .093 |
|  | Time x MEQ Ineffable | .03 | .08 | 27.00 | .40 | .691 |
| Intensity | Intercept | 3.67 | .18 | 34.68 | 20.94 | .000 |
|  | Intensity | .00 | .01 | 34.68 | -.79 | .436 |
|  | Time | .17 | .12 | 27.00 | 1.33 | .194 |
|  | Time x Intensity | .00 | .00 | 27.00 | 1.21 | .235 |
| MEQ Mystical | Intercept | 3.52 | .20 | 35.03 | 17.48 | .000 |
|  | MEQ Mystical | .03 | .10 | 35.03 | .32 | .751 |
|  | Time | .31 | .15 | 27.00 | 2.11 | .044 |
|  | Time x MEQ Mystical | -.02 | .07 | 27.00 | -.29 | .775 |
| BFI Neuroticism | Intercept | 3.80 | .89 | 34.88 | 4.25 | .000 |
|  | BFI N | -.06 | .22 | 34.88 | -.26 | .795 |
|  | Time | -.21 | .64 | 27.00 | -.32 | .749 |
|  | Time x BFI N | .12 | .16 | 27.00 | .76 | .455 |
| BFI Openness | Intercept | .00 | .32 | 54.00 | .00 | 1.000 |
|  | BFI O | 1.00 | .09 | 54.00 | 11.38 | .000 |
|  | Time | 1.34 | .45 | 54.00 | 2.96 | .005 |
|  | Time x BFI O | -.30* | .12 | 54.00 | -2.39 | .020 |
| MEQ Positive mood | Intercept | 3.58 | .19 | 34.91 | 18.37 | .000 |
|  | MEQ Positive mood | .00 | .10 | 34.91 | -.03 | .977 |
|  | Time | .20 | .14 | 27.00 | 1.40 | .173 |
|  | Time x MEQ Positive mood | .05 | .07 | 27.00 | .72 | .477 |
| Prev Psychedelic Use | Intercept | 3.39 | .13 | 35.09 | 25.33 | .000 |
|  | PrevPsychUse | .66 | .25 | 35.09 | 2.59 | .014 |
|  | Time | .38 | .10 | 27.00 | 3.85 | .001 |
|  | Time x PrevPsychUse | -.36 | .19 | 27.00 | -1.96 | .061 |
| BFAS Intellect | Intercept | 2.25 | .46 | 37.77 | 4.89 | .000 |
|  | BFAS I | .38 | .13 | 37.77 | 2.95 | .005 |
|  | Time | .35 | .38 | 27.00 | .91 | .373 |
|  | Time x BFAS I | -.02 | .11 | 27.00 | -.19 | .851 |
| BFAS Openness | Intercept | .92 | .59 | 39.90 | 1.55 | .130 |
|  | BFAS O | .71 | .16 | 39.90 | 4.52 | .000 |
|  | Time | .87 | .54 | 27.00 | 1.63 | .115 |
|  | Time x BFAS O | -.16 | .14 | 27.00 | -1.13 | .269 |
| BIS-B Impulsivity | Intercept | 4.16 | .64 | 35.36 | 6.51 | .000 |
|  | BIS-B | -.21 | .23 | 35.36 | -.94 | .354 |
|  | Time | .36 | .47 | 27.00 | .76 | .452 |
|  | Time x BIS-B | -.03 | .17 | 27.00 | -.18 | .855 |
| MEQ Time-space | Intercept | 3.59 | .19 | 35.02 | 19.11 | .000 |
|  | MEQ Time-space | -.01 | .10 | 35.02 | -.14 | .886 |
|  | Time | .25 | .14 | 27.00 | 1.83 | .079 |
|  | Time x MEQ Time-space | .02 | .07 | 27.00 | .25 | .802 |
| Unemployed | Intercept | 3.58 | .13 | 35.05 | 26.72 | .000 |
|  | Unemployed | -.06 | .32 | 35.05 | -.20 | .846 |
|  | Time | .28 | .10 | 27.00 | 2.86 | .008 |
|  | Time x Unemployed | -.02 | .24 | 27.00 | -.08 | .936 |
| BFAS Openness | | | | | | |
| Age | Intercept | 3.57 | .47 | 31.89 | 7.57 | .000 |
|  | Age | .00 | .01 | 31.89 | .40 | .691 |
|  | Time | .50 | .27 | 27.00 | 1.82 | .080 |
|  | Time x Age | -.01 | .01 | 27.00 | -1.22 | .231 |
| BFI Agreeableness | Intercept | 3.60 | .64 | 32.14 | 5.66 | .000 |
|  | BFI A | .05 | .20 | 32.14 | .26 | .800 |
|  | Time | -.01 | .38 | 27.00 | -.04 | .970 |
|  | Time x BFI A | .06 | .12 | 27.00 | .50 | .618 |
| BFI Conscientiousness | Intercept | 4.07 | .40 | 32.16 | 10.17 | .000 |
|  | BFI C | -.11 | .13 | 32.16 | -.83 | .415 |
|  | Time | .46 | .24 | 27.00 | 1.93 | .065 |
|  | Time x BFI C | -.10 | .08 | 27.00 | -1.25 | .223 |
| Emotional Breakthrough | Intercept | 3.78 | .17 | 32.04 | 22.53 | .000 |
|  | EBI | .00 | .00 | 32.04 | -.19 | .852 |
|  | Time | .11 | .10 | 27.00 | 1.16 | .255 |
|  | Time x EBI | .00 | .00 | 27.00 | .79 | .434 |
| Education level | Intercept | 3.52 | .50 | 32.20 | 7.01 | .000 |
|  | EduLevel | .06 | .12 | 32.20 | .48 | .634 |
|  | Time | .17 | .30 | 27.00 | .58 | .566 |
|  | Time x EduLevel | .00 | .07 | 27.00 | .00 | .997 |
| Emotional insight | Intercept | 3.76 | .16 | 32.02 | 22.94 | .000 |
|  | Emo Insight | .00 | .00 | 32.02 | -.03 | .974 |
|  | Time | .11 | .10 | 27.00 | 1.14 | .265 |
|  | Time x Emo Insight | .00 | .00 | 27.00 | .90 | .374 |
| BFI Extraversion | Intercept | 3.48 | .35 | 32.31 | 9.94 | .000 |
|  | BFI E | .11 | .13 | 32.31 | .82 | .417 |
|  | Time | .12 | .21 | 27.00 | .55 | .586 |
|  | Time x BFI E | .02 | .08 | 27.00 | .28 | .780 |
| Sex | Intercept | 3.67 | .13 | 32.18 | 27.54 | .000 |
|  | Sex | .27 | .24 | 32.18 | 1.15 | .260 |
|  | Time | .21 | .08 | 27.00 | 2.65 | .013 |
|  | Time x Sex | -.12 | .14 | 27.00 | -.85 | .402 |
| MEQ Ineffable | Intercept | 3.88 | .17 | 32.13 | 22.91 | .000 |
|  | MEQ Ineffable | -.09 | .09 | 32.13 | -1.00 | .324 |
|  | Time | .11 | .10 | 27.00 | 1.06 | .297 |
|  | Time x MEQ Ineffable | .05 | .06 | 27.00 | .87 | .392 |
| Intensity | Intercept | 3.86 | .16 | 32.02 | 23.99 | .000 |
|  | Intensity | .00 | .00 | 32.02 | -.92 | .365 |
|  | Time | .10 | .09 | 27.00 | 1.06 | .297 |
|  | Time x Intensity | .00 | .00 | 27.00 | 1.06 | .298 |
| MEQ Mystical | Intercept | 3.84 | .18 | 32.14 | 20.75 | .000 |
|  | MEQ Mystical | -.05 | .09 | 32.14 | -.57 | .574 |
|  | Time | .13 | .11 | 27.00 | 1.16 | .256 |
|  | Time x MEQ Mystical | .03 | .06 | 27.00 | .52 | .607 |
| BFI Neuroticism | Intercept | 3.03 | .81 | 32.34 | 3.73 | .001 |
|  | BFI N | .18 | .20 | 32.34 | .91 | .370 |
|  | Time | .06 | .49 | 27.00 | .13 | .899 |
|  | Time x BFI N | .03 | .12 | 27.00 | .23 | .822 |
| BFI Openness | Intercept | 1.48 | .47 | 36.67 | 3.16 | .003 |
|  | BFI O | .64 | .13 | 36.67 | 4.96 | .000 |
|  | Time | .43 | .37 | 27.00 | 1.17 | .251 |
|  | Time x BFI O | -.07 | .10 | 27.00 | -.72 | .479 |
| MEQ Positive mood | Intercept | 3.83 | .18 | 32.10 | 21.34 | .000 |
|  | MEQ Positive mood | -.04 | .09 | 32.10 | -.50 | .621 |
|  | Time | .12 | .11 | 27.00 | 1.14 | .265 |
|  | Time x MEQ Positive mood | .03 | .05 | 27.00 | .63 | .534 |
| Prev Psychedelic Use | Intercept | 3.64 | .13 | 32.18 | 28.48 | .000 |
|  | PrevPsychUse | .41 | .24 | 32.18 | 1.67 | .104 |
|  | Time | .22 | .08 | 27.00 | 2.94 | .007 |
|  | Time x PrevPsychUse | -.19 | .14 | 27.00 | -1.29 | .209 |
| BFAS Intellect | Intercept | 3.19 | .47 | 32.45 | 6.76 | .000 |
|  | BFAS I | .16 | .13 | 32.45 | 1.23 | .229 |
|  | Time | .20 | .29 | 27.00 | .68 | .500 |
|  | Time x BFAS I | -.01 | .08 | 27.00 | -.09 | .933 |
| BFAS Openness | Intercept | .00 | .26 | 54.00 | .00 | 1.000 |
|  | BFAS O | 1.00 | .07 | 54.00 | 14.78 | .000 |
|  | Time | 1.15 | .36 | 54.00 | 3.16 | .003 |
|  | Time x BFAS O | -.26** | .10 | 54.00 | -2.72 | .009 |
| BIS-B Impulsivity | Intercept | 4.12 | .60 | 32.08 | 6.89 | .000 |
|  | BIS-B | -.13 | .21 | 32.08 | -.61 | .544 |
|  | Time | -.08 | .35 | 27.00 | -.24 | .812 |
|  | Time x BIS-B | .09 | .12 | 27.00 | .74 | .463 |
| MEQ Time-space | Intercept | 3.85 | .17 | 32.03 | 22.34 | .000 |
|  | MEQ Time-space | -.06 | .09 | 32.03 | -.71 | .482 |
|  | Time | .10 | .10 | 27.00 | 1.00 | .327 |
|  | Time x MEQ Time-space | .05 | .05 | 27.00 | .93 | .360 |
| Unemployed | Intercept | 3.75 | .12 | 32.15 | 30.38 | .000 |
|  | Unemployed | .01 | .30 | 32.15 | .02 | .984 |
|  | Time | .18 | .07 | 27.00 | 2.39 | .024 |
|  | Time x Unemployed | -.02 | .18 | 27.00 | -.09 | .933 |
| BFAS Intellect | | | | | | |
| Age | Intercept | 4.14 | .64 | 31.15 | 6.42 | .000 |
|  | Age | -.02 | .02 | 31.15 | -1.05 | .302 |
|  | Time | .04 | .35 | 27.00 | .12 | .907 |
|  | Time x Age | .01 | .01 | 27.00 | .72 | .477 |
| BFI Agreeableness | Intercept | 3.17 | .88 | 31.13 | 3.61 | .001 |
|  | BFI A | .10 | .27 | 31.13 | .35 | .726 |
|  | Time | .16 | .47 | 27.00 | .33 | .743 |
|  | Time x BFI A | .04 | .15 | 27.00 | .28 | .785 |
| BFI Conscientiousness | Intercept | 2.66 | .55 | 31.38 | 4.84 | .000 |
|  | BFI C | .28 | .18 | 31.38 | 1.54 | .132 |
|  | Time | .44 | .30 | 27.00 | 1.44 | .161 |
|  | Time x BFI C | -.05 | .10 | 27.00 | -.53 | .602 |
| Emotional Breakthrough | Intercept | 3.39 | .23 | 31.01 | 14.63 | .000 |
|  | EBI | .00 | .01 | 31.01 | .54 | .592 |
|  | Time | .36 | .12 | 27.00 | 2.96 | .006 |
|  | Time x EBI | .00 | .00 | 27.00 | -.87 | .391 |
| Education level | Intercept | 2.15 | .64 | 31.90 | 3.36 | .002 |
|  | EduLevel | .31 | .15 | 31.90 | 2.13 | .041 |
|  | Time | .16 | .37 | 27.00 | .42 | .679 |
|  | Time x EduLevel | .03 | .09 | 27.00 | .35 | .727 |
| Emotional insight | Intercept | 3.41 | .23 | 30.93 | 15.05 | .000 |
|  | Emo Insight | .00 | .01 | 30.93 | .40 | .691 |
|  | Time | .38 | .12 | 27.00 | 3.21 | .003 |
|  | Time x Emo Insight | .00 | .00 | 27.00 | -1.12 | .272 |
| BFI Extraversion | Intercept | 2.66 | .47 | 31.59 | 5.70 | .000 |
|  | BFI E | .33 | .17 | 31.59 | 1.86 | .072 |
|  | Time | .38 | .26 | 27.00 | 1.44 | .162 |
|  | Time x BFI E | -.04 | .10 | 27.00 | -.38 | .709 |
| Sex | Intercept | 3.43 | .19 | 31.12 | 18.47 | .000 |
|  | Sex | .16 | .33 | 31.12 | .48 | .637 |
|  | Time | .25 | .10 | 27.00 | 2.52 | .018 |
|  | Time x Sex | .11 | .18 | 27.00 | .59 | .558 |
| MEQ Ineffable | Intercept | 3.22 | .23 | 30.88 | 13.90 | .000 |
|  | MEQ Ineffable | .19 | .13 | 30.88 | 1.49 | .147 |
|  | Time | .44 | .12 | 27.00 | 3.63 | .001 |
|  | Time x MEQ Ineffable | -.11 | .07 | 27.00 | -1.69 | .102 |
| Intensity | Intercept | 3.42 | .22 | 31.06 | 15.25 | .000 |
|  | Intensity | .00 | .01 | 31.06 | .38 | .705 |
|  | Time | .34 | .12 | 27.00 | 2.82 | .009 |
|  | Time x Intensity | .00 | .00 | 27.00 | -.62 | .541 |
| MEQ Mystical | Intercept | 3.19 | .25 | 30.59 | 12.68 | .000 |
|  | MEQ Mystical | .18 | .13 | 30.59 | 1.44 | .161 |
|  | Time | .50 | .13 | 27.00 | 4.00 | .000 |
|  | Time x MEQ Mystical | -.14* | .06 | 27.00 | -2.21 | .036 |
| BFI Neuroticism | Intercept | 5.54 | 1.08 | 31.56 | 5.13 | .000 |
|  | BFI N | -.50 | .26 | 31.56 | -1.92 | .063 |
|  | Time | -.07 | .60 | 27.00 | -.12 | .907 |
|  | Time x BFI N | .09 | .15 | 27.00 | .59 | .559 |
| BFI Openness | Intercept | 1.26 | .79 | 31.24 | 1.59 | .122 |
|  | BFI O | .62 | .22 | 31.24 | 2.84 | .008 |
|  | Time | 1.18 | .43 | 27.00 | 2.75 | .010 |
|  | Time x BFI O | -.25* | .12 | 27.00 | -2.13 | .043 |
| MEQ Positive mood | Intercept | 3.21 | .24 | 30.97 | 13.20 | .000 |
|  | MEQ Positive mood | .17 | .12 | 30.97 | 1.45 | .158 |
|  | Time | .43 | .13 | 27.00 | 3.38 | .002 |
|  | Time x MEQ Positive mood | -.09 | .06 | 27.00 | -1.48 | .150 |
| Prev Psychedelic Use | Intercept | 3.39 | .18 | 30.84 | 18.71 | .000 |
|  | PrevPsychUse | .34 | .34 | 30.84 | .98 | .332 |
|  | Time | .36 | .09 | 27.00 | 3.82 | .001 |
|  | Time x PrevPsychUse | -.27 | .18 | 27.00 | -1.51 | .142 |
| BFAS Intellect | Intercept | .00 | .24 | 54.00 | .00 | 1.000 |
|  | BFAS I | 1.00 | .07 | 54.00 | 15.18 | .000 |
|  | Time | .93 | .33 | 54.00 | 2.80 | .007 |
|  | Time x BFAS I | -.19* | .09 | 54.00 | -2.01 | .049 |
| BFAS Openness | Intercept | 2.38 | .95 | 30.95 | 2.52 | .017 |
|  | BFAS O | .29 | .25 | 30.95 | 1.18 | .247 |
|  | Time | .94 | .49 | 27.00 | 1.91 | .067 |
|  | Time x BFAS O | -.18 | .13 | 27.00 | -1.35 | .187 |
| BIS-B Impulsivity | Intercept | 5.41 | .76 | 31.47 | 7.08 | .000 |
|  | BIS-B | -.70 | .27 | 31.47 | -2.58 | .015 |
|  | Time | -.36 | .42 | 27.00 | -.85 | .401 |
|  | Time x BIS-B | .23 | .15 | 27.00 | 1.55 | .133 |
| MEQ Time-space | Intercept | 3.30 | .24 | 30.64 | 13.92 | .000 |
|  | MEQ Time-space | .12 | .12 | 30.64 | .98 | .335 |
|  | Time | .46 | .12 | 27.00 | 3.86 | .001 |
|  | Time x MEQ Time-space | -.12 | .06 | 27.00 | -1.96 | .061 |
| Unemployed | Intercept | 3.63 | .16 | 31.89 | 23.07 | .000 |
|  | Unemployed | -.87 | .38 | 31.89 | -2.29 | .028 |
|  | Time | .27 | .09 | 27.00 | 2.98 | .006 |
|  | Time x Unemployed | .07 | .22 | 27.00 | .32 | .755 |
| Absorption | | | | | | |
| Age | Intercept | 1.84 | .45 | 33.51 | 4.07 | .000 |
|  | Age | .00 | .01 | 33.51 | .41 | .684 |
|  | Time | -.03 | .30 | 27.00 | -.09 | .928 |
|  | Time x Age | .00 | .01 | 27.00 | .41 | .688 |
| BFI Agreeableness | Intercept | 1.48 | .61 | 32.98 | 2.44 | .020 |
|  | BFI A | .17 | .19 | 32.98 | .90 | .377 |
|  | Time | .69 | .39 | 27.00 | 1.78 | .086 |
|  | Time x BFI A | -.19 | .12 | 27.00 | -1.57 | .127 |
| BFI Conscientiousness | Intercept | 2.39 | .39 | 32.82 | 6.10 | .000 |
|  | BFI C | -.13 | .13 | 32.82 | -.99 | .329 |
|  | Time | -.34 | .25 | 27.00 | -1.38 | .178 |
|  | Time x BFI C | .15 | .08 | 27.00 | 1.82 | .080 |
| Emotional Breakthrough | Intercept | 1.96 | .16 | 33.15 | 12.45 | .000 |
|  | EBI | .00 | .00 | 33.15 | .51 | .616 |
|  | Time | -.03 | .10 | 27.00 | -.33 | .746 |
|  | Time x EBI | .00 | .00 | 27.00 | 1.64 | .112 |
| Education level | Intercept | 2.15 | .48 | 33.35 | 4.44 | .000 |
|  | EduLevel | -.03 | .11 | 33.35 | -.28 | .784 |
|  | Time | -.13 | .32 | 27.00 | -.43 | .673 |
|  | Time x EduLevel | .05 | .07 | 27.00 | .73 | .471 |
| Emotional insight | Intercept | 1.91 | .15 | 33.68 | 12.51 | .000 |
|  | Emo Insight | .00 | .00 | 33.68 | .99 | .331 |
|  | Time | .02 | .10 | 27.00 | .15 | .882 |
|  | Time x Emo Insight | .00 | .00 | 27.00 | 1.01 | .321 |
| BFI Extraversion | Intercept | 1.58 | .34 | 33.17 | 4.71 | .000 |
|  | BFI E | .17 | .13 | 33.17 | 1.39 | .174 |
|  | Time | .40 | .22 | 27.00 | 1.85 | .075 |
|  | Time x BFI E | -.12 | .08 | 27.00 | -1.51 | .142 |
| Sex | Intercept | 2.00 | .13 | 33.48 | 15.42 | .000 |
|  | Sex | .07 | .23 | 33.48 | .29 | .775 |
|  | Time | .07 | .09 | 27.00 | .85 | .401 |
|  | Time x Sex | .06 | .15 | 27.00 | .37 | .714 |
| MEQ Ineffable | Intercept | 2.06 | .16 | 32.83 | 12.60 | .000 |
|  | MEQ Ineffable | -.03 | .09 | 32.83 | -.36 | .719 |
|  | Time | -.05 | .10 | 27.00 | -.44 | .664 |
|  | Time x MEQ Ineffable | .10 | .06 | 27.00 | 1.75 | .092 |
| Intensity | Intercept | 2.07 | .16 | 32.95 | 13.32 | .000 |
|  | Intensity | .00 | .00 | 32.95 | -.44 | .664 |
|  | Time | -.02 | .10 | 27.00 | -.21 | .838 |
|  | Time x Intensity | .00 | .00 | 27.00 | 1.56 | .130 |
| MEQ Mystical | Intercept | 2.04 | .18 | 32.96 | 11.53 | .000 |
|  | MEQ Mystical | -.01 | .09 | 32.96 | -.17 | .869 |
|  | Time | -.05 | .11 | 27.00 | -.45 | .654 |
|  | Time x MEQ Mystical | .09 | .06 | 27.00 | 1.58 | .125 |
| BFI Neuroticism | Intercept | 1.17 | .77 | 33.82 | 1.52 | .138 |
|  | BFI N | .21 | .19 | 33.82 | 1.11 | .277 |
|  | Time | -.04 | .52 | 27.00 | -.08 | .937 |
|  | Time x BFI N | .03 | .13 | 27.00 | .26 | .800 |
| BFI Openness | Intercept | .64 | .55 | 35.02 | 1.17 | .248 |
|  | BFI O | .39 | .15 | 35.02 | 2.56 | .015 |
|  | Time | .21 | .40 | 27.00 | .54 | .594 |
|  | Time x BFI O | -.03 | .11 | 27.00 | -.32 | .754 |
| MEQ Positive mood | Intercept | 2.05 | .17 | 32.64 | 12.01 | .000 |
|  | MEQ Positive mood | -.02 | .08 | 32.64 | -.25 | .807 |
|  | Time | -.08 | .11 | 27.00 | -.75 | .458 |
|  | Time x MEQ Positive mood | .11* | .05 | 27.00 | 2.06 | .049 |
| Prev Psychedelic Use | Intercept | 1.89 | .12 | 33.13 | 15.50 | .000 |
|  | PrevPsychUse | .45 | .23 | 33.13 | 1.93 | .062 |
|  | Time | .17 | .08 | 27.00 | 2.15 | .041 |
|  | Time x PrevPsychUse | -.28 | .15 | 27.00 | -1.90 | .069 |
| BFAS Intellect | Intercept | 2.05 | .47 | 33.42 | 4.42 | .000 |
|  | BFAS I | -.01 | .13 | 33.42 | -.08 | .937 |
|  | Time | -.05 | .31 | 27.00 | -.17 | .863 |
|  | Time x BFAS I | .04 | .09 | 27.00 | .48 | .632 |
| BFAS Openness | Intercept | -.21 | .53 | 37.74 | -.40 | .695 |
|  | BFAS O | .59 | .14 | 37.74 | 4.29 | .000 |
|  | Time | .35 | .44 | 27.00 | .81 | .427 |
|  | Time x BFAS O | -.07 | .11 | 27.00 | -.61 | .549 |
| BIS-B Impulsivity | Intercept | 2.02 | .57 | 32.55 | 3.57 | .001 |
|  | BIS-B | .00 | .20 | 32.55 | .00 | .999 |
|  | Time | .87 | .35 | 27.00 | 2.51 | .018 |
|  | Time x BIS-B | -.28* | .12 | 27.00 | -2.29 | .030 |
| MEQ Time-space | Intercept | 2.03 | .17 | 33.00 | 12.30 | .000 |
|  | MEQ Time-space | -.01 | .08 | 33.00 | -.10 | .920 |
|  | Time | -.03 | .11 | 27.00 | -.31 | .761 |
|  | Time x MEQ Time-space | .08 | .05 | 27.00 | 1.54 | .136 |
| Unemployed | Intercept | 2.01 | .12 | 33.29 | 17.06 | .000 |
|  | Unemployed | .07 | .28 | 33.29 | .24 | .812 |
|  | Time | .06 | .08 | 27.00 | .73 | .474 |
|  | Time x Unemployed | .20 | .18 | 27.00 | 1.10 | .281 |
| Agreeableness | | | | | | |
| Age | Intercept | 2.41 | .41 | 41.64 | 5.83 | .000 |
|  | Age | .02 | .01 | 41.64 | 1.91 | .063 |
|  | Time | .07 | .39 | 27.00 | .19 | .852 |
|  | Time x Age | .00 | .01 | 27.00 | .50 | .624 |
| BFI Agreeableness | Intercept | .00 | .33 | 54.00 | .00 | 1.000 |
|  | BFI A | 1.00 | .10 | 54.00 | 9.84 | .000 |
|  | Time | 1.62 | .46 | 53.94 | 3.49 | .001 |
|  | Time x BFI A | -.43*** | .14 | 53.94 | -2.97 | .004 |
| BFI Conscientiousness | Intercept | 2.86 | .39 | 39.46 | 7.40 | .000 |
|  | BFI C | .11 | .13 | 39.46 | .84 | .408 |
|  | Time | .45 | .34 | 27.00 | 1.32 | .199 |
|  | Time x BFI C | -.06 | .11 | 27.00 | -.57 | .574 |
| Emotional Breakthrough | Intercept | 3.16 | .16 | 39.43 | 19.91 | .000 |
|  | EBI | .00 | .00 | 39.43 | .15 | .882 |
|  | Time | .24 | .14 | 27.00 | 1.71 | .098 |
|  | Time x EBI | .00 | .00 | 27.00 | .23 | .821 |
| Education level | Intercept | 2.97 | .47 | 39.55 | 6.26 | .000 |
|  | EduLevel | .05 | .11 | 39.55 | .44 | .660 |
|  | Time | .19 | .42 | 27.00 | .45 | .657 |
|  | Time x EduLevel | .02 | .10 | 27.00 | .18 | .857 |
| Emotional insight | Intercept | 3.16 | .16 | 39.44 | 20.33 | .000 |
|  | Emo Insight | .00 | .00 | 39.44 | .17 | .866 |
|  | Time | .25 | .14 | 27.00 | 1.82 | .080 |
|  | Time x Emo Insight | .00 | .00 | 27.00 | .14 | .891 |
| BFI Extraversion | Intercept | 2.92 | .33 | 39.51 | 8.73 | .000 |
|  | BFI E | .10 | .13 | 39.51 | .82 | .416 |
|  | Time | .40 | .30 | 27.00 | 1.35 | .187 |
|  | Time x BFI E | -.05 | .11 | 27.00 | -.49 | .630 |
| Sex | Intercept | 3.18 | .13 | 39.42 | 24.87 | .000 |
|  | Sex | -.02 | .23 | 39.42 | -.10 | .921 |
|  | Time | .28 | .11 | 27.00 | 2.45 | .021 |
|  | Time x Sex | -.04 | .20 | 27.00 | -.21 | .833 |
| MEQ Ineffable | Intercept | 3.24 | .16 | 39.46 | 20.00 | .000 |
|  | MEQ Ineffable | -.04 | .09 | 39.46 | -.49 | .625 |
|  | Time | .23 | .14 | 27.00 | 1.62 | .116 |
|  | Time x MEQ Ineffable | .02 | .08 | 27.00 | .29 | .773 |
| Intensity | Intercept | 3.18 | .15 | 39.26 | 20.77 | .000 |
|  | Intensity | .00 | .00 | 39.26 | -.06 | .955 |
|  | Time | .20 | .13 | 27.00 | 1.46 | .157 |
|  | Time x Intensity | .00 | .00 | 27.00 | .70 | .491 |
| MEQ Mystical | Intercept | 3.23 | .17 | 39.52 | 18.49 | .000 |
|  | MEQ Mystical | -.03 | .09 | 39.52 | -.39 | .701 |
|  | Time | .31 | .16 | 27.00 | 1.98 | .058 |
|  | Time x MEQ Mystical | -.03 | .08 | 27.00 | -.35 | .732 |
| BFI Neuroticism | Intercept | 4.11 | .77 | 39.85 | 5.36 | .000 |
|  | BFI N | -.23 | .18 | 39.85 | -1.23 | .226 |
|  | Time | -.06 | .69 | 27.00 | -.09 | .930 |
|  | Time x BFI N | .08 | .17 | 27.00 | .48 | .637 |
| BFI Openness | Intercept | 3.09 | .59 | 39.17 | 5.21 | .000 |
|  | BFI O | .02 | .16 | 39.17 | .15 | .883 |
|  | Time | .69 | .52 | 27.00 | 1.32 | .198 |
|  | Time x BFI O | -.12 | .14 | 27.00 | -.82 | .417 |
| MEQ Positive mood | Intercept | 3.25 | .17 | 39.52 | 19.16 | .000 |
|  | MEQ Positive mood | -.04 | .08 | 39.52 | -.52 | .605 |
|  | Time | .25 | .15 | 27.00 | 1.63 | .115 |
|  | Time x MEQ Positive mood | .01 | .07 | 27.00 | .16 | .871 |
| Prev Psychedelic Use | Intercept | 3.23 | .12 | 39.85 | 26.47 | .000 |
|  | PrevPsychUse | -.19 | .23 | 39.85 | -.80 | .428 |
|  | Time | .31 | .11 | 27.00 | 2.80 | .009 |
|  | Time x PrevPsychUse | -.15 | .21 | 27.00 | -.74 | .467 |
| BFAS Intellect | Intercept | 3.02 | .46 | 39.31 | 6.59 | .000 |
|  | BFAS I | .05 | .13 | 39.31 | .35 | .725 |
|  | Time | .48 | .40 | 27.00 | 1.19 | .246 |
|  | Time x BFAS I | -.06 | .11 | 27.00 | -.55 | .590 |
| BFAS Openness | Intercept | 3.02 | .65 | 39.03 | 4.61 | .000 |
|  | BFAS O | .04 | .17 | 39.03 | .25 | .804 |
|  | Time | .84 | .57 | 27.00 | 1.47 | .154 |
|  | Time x BFAS O | -.15 | .15 | 27.00 | -1.02 | .319 |
| BIS-B Impulsivity | Intercept | 3.31 | .57 | 39.46 | 5.84 | .000 |
|  | BIS-B | -.05 | .20 | 39.46 | -.24 | .810 |
|  | Time | .30 | .50 | 27.00 | .60 | .555 |
|  | Time x BIS-B | -.01 | .18 | 27.00 | -.07 | .943 |
| MEQ Time-space | Intercept | 3.23 | .16 | 39.52 | 19.77 | .000 |
|  | MEQ Time-space | -.03 | .08 | 39.52 | -.39 | .696 |
|  | Time | .28 | .14 | 27.00 | 1.96 | .060 |
|  | Time x MEQ Time-space | -.01 | .07 | 27.00 | -.18 | .855 |
| Unemployed | Intercept | 3.19 | .12 | 38.89 | 27.44 | .000 |
|  | Unemployed | -.05 | .28 | 38.89 | -.19 | .854 |
|  | Time | .21 | .10 | 27.00 | 2.11 | .044 |
|  | Time x Unemployed | .30 | .24 | 27.00 | 1.23 | .230 |
| Conscientiousness | | | | | | |
| Age | Intercept | 1.99 | .63 | 33.04 | 3.15 | .003 |
|  | Age | .02 | .02 | 33.04 | 1.53 | .137 |
|  | Time | .10 | .40 | 27.00 | .24 | .811 |
|  | Time x Age | .00 | .01 | 27.00 | .31 | .759 |
| BFI Agreeableness | Intercept | 2.21 | .88 | 32.64 | 2.52 | .017 |
|  | BFI A | .23 | .27 | 32.64 | .83 | .411 |
|  | Time | -.07 | .54 | 27.00 | -.13 | .895 |
|  | Time x BFI A | .09 | .17 | 27.00 | .54 | .590 |
| BFI Conscientiousness | Intercept | .00 | .23 | 54.00 | .00 | 1.000 |
|  | BFI C | 1.00 | .08 | 54.00 | 13.04 | .000 |
|  | Time | .83 | .33 | 53.99 | 2.52 | .015 |
|  | Time x BFI C | -.21 | .11 | 53.99 | -1.93 | .059 |
| Emotional Breakthrough | Intercept | 2.97 | .23 | 32.38 | 12.65 | .000 |
|  | EBI | .00 | .01 | 32.38 | -.25 | .802 |
|  | Time | .14 | .14 | 27.00 | 1.01 | .324 |
|  | Time x EBI | .00 | .00 | 27.00 | .72 | .480 |
| Education level | Intercept | 1.26 | .63 | 34.09 | 2.01 | .052 |
|  | EduLevel | .39 | .14 | 34.09 | 2.73 | .010 |
|  | Time | .27 | .43 | 27.00 | .62 | .542 |
|  | Time x EduLevel | -.01 | .10 | 27.00 | -.11 | .911 |
| Emotional insight | Intercept | 3.06 | .23 | 32.55 | 13.42 | .000 |
|  | Emo Insight | .00 | .01 | 32.55 | -.80 | .427 |
|  | Time | .18 | .14 | 27.00 | 1.29 | .210 |
|  | Time x Emo Insight | .00 | .00 | 27.00 | .38 | .709 |
| BFI Extraversion | Intercept | 3.26 | .50 | 32.09 | 6.58 | .000 |
|  | BFI E | -.13 | .19 | 32.09 | -.71 | .484 |
|  | Time | -.19 | .29 | 27.00 | -.65 | .521 |
|  | Time x BFI E | .16 | .11 | 27.00 | 1.47 | .152 |
| Sex | Intercept | 2.71 | .18 | 33.35 | 15.37 | .000 |
|  | Sex | .71 | .32 | 33.35 | 2.26 | .030 |
|  | Time | .26 | .11 | 27.00 | 2.22 | .035 |
|  | Time x Sex | -.12 | .21 | 27.00 | -.58 | .566 |
| MEQ Ineffable | Intercept | 2.97 | .24 | 32.39 | 12.36 | .000 |
|  | MEQ Ineffable | -.03 | .13 | 32.39 | -.21 | .834 |
|  | Time | .14 | .15 | 27.00 | .99 | .332 |
|  | Time x MEQ Ineffable | .06 | .08 | 27.00 | .68 | .500 |
| Intensity | Intercept | 3.07 | .23 | 32.39 | 13.60 | .000 |
|  | Intensity | -.01 | .01 | 32.39 | -.86 | .395 |
|  | Time | .13 | .14 | 27.00 | .94 | .355 |
|  | Time x Intensity | .00 | .00 | 27.00 | .91 | .370 |
| MEQ Mystical | Intercept | 3.03 | .26 | 32.47 | 11.68 | .000 |
|  | MEQ Mystical | -.06 | .13 | 32.47 | -.49 | .629 |
|  | Time | .17 | .16 | 27.00 | 1.05 | .304 |
|  | Time x MEQ Mystical | .03 | .08 | 27.00 | .42 | .679 |
| BFI Neuroticism | Intercept | 3.59 | 1.13 | 32.29 | 3.18 | .003 |
|  | BFI N | -.16 | .27 | 32.29 | -.59 | .559 |
|  | Time | 1.21 | .68 | 27.00 | 1.79 | .085 |
|  | Time x BFI N | -.24 | .16 | 27.00 | -1.48 | .151 |
| BFI Openness | Intercept | 4.05 | .86 | 32.75 | 4.72 | .000 |
|  | BFI O | -.31 | .24 | 32.75 | -1.33 | .192 |
|  | Time | -.02 | .54 | 27.00 | -.05 | .964 |
|  | Time x BFI O | .07 | .15 | 27.00 | .46 | .648 |
| MEQ Positive mood | Intercept | 2.95 | .25 | 32.44 | 11.72 | .000 |
|  | MEQ Positive mood | -.01 | .13 | 32.44 | -.11 | .913 |
|  | Time | .17 | .15 | 27.00 | 1.10 | .281 |
|  | Time x MEQ Positive mood | .03 | .08 | 27.00 | .42 | .677 |
| Prev Psychedelic Use | Intercept | 3.01 | .18 | 32.19 | 16.94 | .000 |
|  | PrevPsychUse | -.28 | .34 | 32.19 | -.84 | .408 |
|  | Time | .33 | .11 | 27.00 | 3.11 | .004 |
|  | Time x PrevPsychUse | -.40 | .20 | 27.00 | -1.98 | .058 |
| BFAS Intellect | Intercept | 1.96 | .64 | 33.06 | 3.06 | .004 |
|  | BFAS I | .28 | .18 | 33.06 | 1.57 | .127 |
|  | Time | -.15 | .41 | 27.00 | -.36 | .721 |
|  | Time x BFAS I | .11 | .11 | 27.00 | .92 | .365 |
| BFAS Openness | Intercept | 3.67 | .96 | 32.63 | 3.83 | .001 |
|  | BFAS O | -.20 | .25 | 32.63 | -.78 | .439 |
|  | Time | .43 | .59 | 27.00 | .73 | .471 |
|  | Time x BFAS O | -.06 | .16 | 27.00 | -.37 | .717 |
| BIS-B Impulsivity | Intercept | 5.66 | .67 | 35.68 | 8.40 | .000 |
|  | BIS-B | -.99 | .24 | 35.68 | -4.13 | .000 |
|  | Time | -.14 | .51 | 27.00 | -.28 | .779 |
|  | Time x BIS-B | .13 | .18 | 27.00 | .73 | .474 |
| MEQ Time-space | Intercept | 3.01 | .24 | 32.52 | 12.46 | .000 |
|  | MEQ Time-space | -.06 | .12 | 32.52 | -.46 | .648 |
|  | Time | .20 | .15 | 27.00 | 1.39 | .177 |
|  | Time x MEQ Time-space | .01 | .08 | 27.00 | .12 | .906 |
| Unemployed | Intercept | 2.98 | .17 | 32.59 | 17.37 | .000 |
|  | Unemployed | -.29 | .41 | 32.59 | -.70 | .490 |
|  | Time | .22 | .11 | 27.00 | 2.11 | .045 |
|  | Time x Unemployed | -.02 | .25 | 27.00 | -.09 | .931 |
| BIS-B Impulsivity | | | | | | |
| Age | Intercept | 3.42 | .39 | 34.76 | 8.70 | .000 |
|  | Age | -.01 | .01 | 34.76 | -.96 | .345 |
|  | Time | -.32 | .28 | 27.00 | -1.14 | .265 |
|  | Time x Age | .00 | .01 | 27.00 | -.10 | .918 |
| BFI Agreeableness | Intercept | 3.14 | .54 | 34.29 | 5.86 | .000 |
|  | BFI A | -.03 | .17 | 34.29 | -.17 | .869 |
|  | Time | .01 | .37 | 27.00 | .04 | .969 |
|  | Time x BFI A | -.11 | .12 | 27.00 | -.99 | .330 |
| BFI Conscientiousness | Intercept | 4.15 | .26 | 41.21 | 16.04 | .000 |
|  | BFI C | -.37 | .08 | 41.21 | -4.38 | .000 |
|  | Time | -.19 | .24 | 27.00 | -.77 | .451 |
|  | Time x BFI C | -.06 | .08 | 27.00 | -.70 | .492 |
| Emotional Breakthrough | Intercept | 3.14 | .14 | 33.87 | 22.24 | .000 |
|  | EBI | .00 | .00 | 33.87 | -.82 | .416 |
|  | Time | -.46 | .10 | 27.00 | -4.83 | .000 |
|  | Time x EBI | .00 | .00 | 27.00 | 1.59 | .124 |
| Education level | Intercept | 3.37 | .42 | 34.54 | 8.11 | .000 |
|  | EduLevel | -.07 | .10 | 34.54 | -.77 | .445 |
|  | Time | -.03 | .29 | 27.00 | -.10 | .920 |
|  | Time x EduLevel | -.08 | .07 | 27.00 | -1.12 | .274 |
| Emotional insight | Intercept | 3.05 | .14 | 34.39 | 21.94 | .000 |
|  | Emo Insight | .00 | .00 | 34.39 | .10 | .921 |
|  | Time | -.39 | .10 | 27.00 | -4.05 | .000 |
|  | Time x Emo Insight | .00 | .00 | 27.00 | .63 | .535 |
| BFI Extraversion | Intercept | 2.96 | .30 | 34.49 | 9.85 | .000 |
|  | BFI E | .04 | .11 | 34.49 | .33 | .747 |
|  | Time | -.33 | .21 | 27.00 | -1.56 | .131 |
|  | Time x BFI E | -.01 | .08 | 27.00 | -.10 | .923 |
| Sex | Intercept | 3.08 | .11 | 34.47 | 26.83 | .000 |
|  | Sex | -.06 | .21 | 34.47 | -.30 | .768 |
|  | Time | -.36 | .08 | 27.00 | -4.42 | .000 |
|  | Time x Sex | .02 | .14 | 27.00 | .16 | .875 |
| MEQ Ineffable | Intercept | 3.14 | .14 | 34.50 | 21.72 | .000 |
|  | MEQ Ineffable | -.06 | .08 | 34.50 | -.73 | .468 |
|  | Time | -.39 | .10 | 27.00 | -3.80 | .001 |
|  | Time x MEQ Ineffable | .03 | .06 | 27.00 | .49 | .631 |
| Intensity | Intercept | 3.06 | .14 | 34.45 | 22.27 | .000 |
|  | Intensity | .00 | .00 | 34.45 | -.08 | .933 |
|  | Time | -.36 | .10 | 27.00 | -3.74 | .001 |
|  | Time x Intensity | .00 | .00 | 27.00 | .17 | .864 |
| MEQ Mystical | Intercept | 3.15 | .16 | 34.39 | 20.15 | .000 |
|  | MEQ Mystical | -.06 | .08 | 34.39 | -.77 | .447 |
|  | Time | -.42 | .11 | 27.00 | -3.80 | .001 |
|  | Time x MEQ Mystical | .04 | .06 | 27.00 | .76 | .451 |
| BFI Neuroticism | Intercept | 3.09 | .70 | 34.46 | 4.42 | .000 |
|  | BFI N | -.01 | .17 | 34.46 | -.05 | .961 |
|  | Time | -.26 | .49 | 27.00 | -.54 | .595 |
|  | Time x BFI N | -.02 | .12 | 27.00 | -.17 | .862 |
| BFI Openness | Intercept | 3.49 | .53 | 34.29 | 6.58 | .000 |
|  | BFI O | -.12 | .15 | 34.29 | -.82 | .416 |
|  | Time | -.70 | .37 | 27.00 | -1.90 | .069 |
|  | Time x BFI O | .10 | .10 | 27.00 | .96 | .343 |
| MEQ Positive mood | Intercept | 3.15 | .15 | 34.58 | 20.89 | .000 |
|  | MEQ Positive mood | -.06 | .08 | 34.58 | -.81 | .422 |
|  | Time | -.38 | .11 | 27.00 | -3.52 | .002 |
|  | Time x MEQ Positive mood | .02 | .05 | 27.00 | .33 | .745 |
| Prev Psychedelic Use | Intercept | 3.05 | .11 | 34.46 | 27.28 | .000 |
|  | PrevPsychUse | .01 | .21 | 34.46 | .04 | .967 |
|  | Time | -.35 | .08 | 27.00 | -4.46 | .000 |
|  | Time x PrevPsychUse | .01 | .15 | 27.00 | .05 | .961 |
| BFAS Intellect | Intercept | 3.98 | .36 | 36.68 | 10.90 | .000 |
|  | BFAS I | -.26 | .10 | 36.68 | -2.59 | .014 |
|  | Time | -.28 | .29 | 27.00 | -.97 | .340 |
|  | Time x BFAS I | -.02 | .08 | 27.00 | -.25 | .807 |
| BFAS Openness | Intercept | 3.22 | .59 | 34.39 | 5.48 | .000 |
|  | BFAS O | -.04 | .15 | 34.39 | -.28 | .781 |
|  | Time | -.56 | .41 | 27.00 | -1.37 | .181 |
|  | Time x BFAS O | .06 | .11 | 27.00 | .53 | .599 |
| BIS-B Impulsivity | Intercept | .69 | .26 | 53.88 | 2.70 | .009 |
|  | BIS-B | .85 | .09 | 53.88 | 9.39 | .000 |
|  | Time | -.09 | .35 | 27.00 | -.26 | .794 |
|  | Time x BIS-B | -.09 | .13 | 27.00 | -.74 | .468 |
| MEQ Time-space | Intercept | 3.13 | .15 | 34.39 | 21.45 | .000 |
|  | MEQ Time-space | -.05 | .07 | 34.39 | -.69 | .493 |
|  | Time | -.40 | .10 | 27.00 | -3.95 | .001 |
|  | Time x MEQ Time-space | .04 | .05 | 27.00 | .71 | .486 |
| Unemployed | Intercept | 3.05 | .10 | 34.42 | 29.20 | .000 |
|  | Unemployed | .02 | .25 | 34.42 | .09 | .928 |
|  | Time | -.36 | .07 | 27.00 | -4.98 | .000 |
|  | Time x Unemployed | .09 | .18 | 27.00 | .51 | .616 |
| **Psilocybin Therapy** | | | | | | |
| Neuroticism | | | | | | |
| Age | Intercept | 4.37 | .56 | 44.49 | 7.78 | .000 |
|  | Age | -.01 | .01 | 44.49 | -.74 | .462 |
|  | Time | -.74 | .56 | 28.00 | -1.33 | .196 |
|  | Time x Age | .00 | .01 | 28.00 | .37 | .713 |
| BFI Agreeableness | Intercept | 5.22 | .61 | 46.60 | 8.51 | .000 |
|  | BFI A | -.39 | .19 | 46.60 | -2.10 | .041 |
|  | Time | -.75 | .64 | 28.00 | -1.16 | .257 |
|  | Time x BFI A | .06 | .20 | 28.00 | .33 | .743 |
| BFI Conscientiousness | Intercept | 4.14 | .64 | 43.84 | 6.47 | .000 |
|  | BFI C | -.06 | .20 | 43.84 | -.28 | .779 |
|  | Time | -1.21 | .62 | 28.00 | -1.94 | .063 |
|  | Time x BFI C | .22 | .20 | 28.00 | 1.10 | .280 |
| Emotional Breakthrough | Intercept | 3.89 | .38 | 43.46 | 10.22 | .000 |
|  | EBI | .00 | .00 | 43.46 | .20 | .840 |
|  | Time | -.01 | .37 | 28.00 | -.03 | .979 |
|  | Time x EBI | -.01 | .00 | 28.00 | -1.55 | .131 |
| Education level | Intercept | 3.64 | .52 | 44.03 | 6.99 | .000 |
|  | EduLevel | .09 | .14 | 44.03 | .64 | .524 |
|  | Time | -.11 | .51 | 28.00 | -.21 | .834 |
|  | Time x EduLevel | -.12 | .13 | 28.00 | -.88 | .388 |
| Emotional insight | Intercept | 4.02 | .55 | 43.63 | 7.33 | .000 |
|  | Emo Insight | .00 | .01 | 43.63 | -.10 | .920 |
|  | Time | .30 | .53 | 28.00 | .57 | .572 |
|  | Time x Emo Insight | -.01 | .01 | 28.00 | -1.64 | .112 |
| BFI Extraversion | Intercept | 4.02 | .38 | 44.32 | 10.58 | .000 |
|  | BFI E | -.02 | .15 | 44.32 | -.16 | .872 |
|  | Time | -.33 | .38 | 28.00 | -.88 | .388 |
|  | Time x BFI E | -.09 | .14 | 28.00 | -.60 | .552 |
| Sex | Intercept | 3.78 | .18 | 43.54 | 21.23 | .000 |
|  | Sex | .50 | .29 | 43.54 | 1.71 | .094 |
|  | Time | -.36 | .17 | 28.00 | -2.10 | .045 |
|  | Time x Sex | -.48 | .28 | 28.00 | -1.70 | .100 |
| MEQ Ineffable | Intercept | 3.85 | .41 | 43.40 | 9.35 | .000 |
|  | MEQ Ineffable | .03 | .11 | 43.40 | .31 | .756 |
|  | Time | .04 | .39 | 28.00 | .09 | .929 |
|  | Time x MEQ Ineffable | -.16 | .10 | 28.00 | -1.55 | .132 |
| Intensity | Intercept | 4.30 | .74 | 44.43 | 5.85 | .000 |
|  | Intensity | .00 | .01 | 44.43 | -.46 | .650 |
|  | Time | .23 | .73 | 28.00 | .31 | .758 |
|  | Time x Intensity | -.01 | .01 | 28.00 | -1.07 | .293 |
| MEQ Mystical | Intercept | 3.70 | .46 | 43.35 | 8.03 | .000 |
|  | MEQ Mystical | .07 | .11 | 43.35 | .61 | .546 |
|  | Time | .09 | .44 | 28.00 | .21 | .835 |
|  | Time x MEQ Mystical | -.17 | .11 | 28.00 | -1.50 | .144 |
| BFI Neuroticism | Intercept | .00 | .52 | 56.00 | .00 | 1.000 |
|  | BFI N | 1.00 | .13 | 56.00 | 7.79 | .000 |
|  | Time | 1.14 | .73 | 55.93 | 1.55 | .127 |
|  | Time x BFI N | -.42* | .18 | 55.93 | -2.32 | .024 |
| BFI Openness | Intercept | 3.20 | .71 | 43.82 | 4.50 | .000 |
|  | BFI O | .22 | .20 | 43.82 | 1.10 | .279 |
|  | Time | .30 | .69 | 28.00 | .43 | .673 |
|  | Time x BFI O | -.24 | .19 | 28.00 | -1.23 | .229 |
| MEQ Positive mood | Intercept | 3.71 | .46 | 43.47 | 8.10 | .000 |
|  | MEQ Positive mood | .07 | .11 | 43.47 | .60 | .551 |
|  | Time | .05 | .44 | 28.00 | .11 | .912 |
|  | Time x MEQ Positive mood | -.15 | .11 | 28.00 | -1.41 | .171 |
| Prev Psychedelic Use | Intercept | 4.05 | .17 | 42.77 | 23.62 | .000 |
|  | PrevPsychUse | -.27 | .31 | 42.77 | -.86 | .393 |
|  | Time | -.71 | .16 | 28.00 | -4.38 | .000 |
|  | Time x PrevPsychUse | .56 | .29 | 28.00 | 1.92 | .066 |
| BFAS Intellect | Intercept | 3.17 | .67 | 43.89 | 4.72 | .000 |
|  | BFAS I | .23 | .19 | 43.89 | 1.21 | .233 |
|  | Time | .25 | .65 | 28.00 | .38 | .706 |
|  | Time x BFAS I | -.23 | .19 | 28.00 | -1.23 | .229 |
| BFAS Openness | Intercept | 3.22 | .80 | 44.67 | 4.00 | .000 |
|  | BFAS O | .20 | .21 | 44.67 | .95 | .349 |
|  | Time | -.25 | .80 | 28.00 | -.32 | .754 |
|  | Time x BFAS O | -.08 | .21 | 28.00 | -.36 | .721 |
| BIS-B Impulsivity | Intercept | 3.03 | .89 | 42.18 | 3.39 | .002 |
|  | BIS-B | .34 | .32 | 42.18 | 1.06 | .294 |
|  | Time | 1.32 | .83 | 28.00 | 1.60 | .121 |
|  | Time x BIS-B | -.67* | .30 | 28.00 | -2.28 | .030 |
| MEQ Time-space | Intercept | 3.77 | .42 | 43.28 | 9.06 | .000 |
|  | MEQ Time-space | .05 | .10 | 43.28 | .49 | .625 |
|  | Time | .06 | .40 | 28.00 | .14 | .890 |
|  | Time x MEQ Time-space | -.16 | .10 | 28.00 | -1.59 | .124 |
| Unemployed | Intercept | 3.86 | .16 | 45.34 | 23.91 | .000 |
|  | Unemployed | .44 | .33 | 45.34 | 1.32 | .192 |
|  | Time | -.53 | .16 | 28.00 | -3.22 | .003 |
|  | Time x Unemployed | -.04 | .34 | 28.00 | -.13 | .899 |
| Extraversion | | | | | | |
| Age | Intercept | 2.27 | .73 | 31.68 | 3.09 | .004 |
|  | Age | .00 | .02 | 31.68 | .18 | .856 |
|  | Time | .86 | .37 | 28.00 | 2.36 | .025 |
|  | Time x Age | -.01 | .01 | 28.00 | -1.36 | .185 |
| BFI Agreeableness | Intercept | 3.25 | .82 | 31.87 | 3.98 | .000 |
|  | BFI A | -.27 | .25 | 31.87 | -1.07 | .292 |
|  | Time | 1.05 | .42 | 28.00 | 2.54 | .017 |
|  | Time x BFI A | -.21 | .13 | 28.00 | -1.66 | .109 |
| BFI Conscientiousness | Intercept | 2.41 | .84 | 31.91 | 2.87 | .007 |
|  | BFI C | -.01 | .26 | 31.91 | -.02 | .985 |
|  | Time | .39 | .43 | 28.00 | .91 | .370 |
|  | Time x BFI C | .00 | .13 | 28.00 | -.02 | .985 |
| Emotional Breakthrough | Intercept | 2.42 | .50 | 31.88 | 4.82 | .000 |
|  | EBI | .00 | .01 | 31.88 | -.06 | .952 |
|  | Time | .26 | .26 | 28.00 | 1.01 | .319 |
|  | Time x EBI | .00 | .00 | 28.00 | .52 | .610 |
| Education level | Intercept | 2.67 | .68 | 31.34 | 3.94 | .000 |
|  | EduLevel | -.07 | .18 | 31.34 | -.42 | .674 |
|  | Time | -.30 | .32 | 28.00 | -.93 | .362 |
|  | Time x EduLevel | .18* | .08 | 28.00 | 2.20 | .036 |
| Emotional insight | Intercept | 2.33 | .73 | 31.79 | 3.19 | .003 |
|  | Emo Insight | .00 | .01 | 31.79 | .09 | .926 |
|  | Time | .01 | .37 | 28.00 | .04 | .971 |
|  | Time x Emo Insight | .00 | .00 | 28.00 | 1.04 | .307 |
| BFI Extraversion | Intercept | .00 | .18 | 56.00 | .00 | 1.000 |
|  | BFI E | 1.00 | .07 | 56.00 | 14.65 | .000 |
|  | Time | .63 | .25 | 56.00 | 2.51 | .015 |
|  | Time x BFI E | -.10 | .10 | 56.00 | -1.06 | .295 |
| Sex | Intercept | 2.10 | .22 | 32.55 | 9.50 | .000 |
|  | Sex | .81 | .36 | 32.55 | 2.22 | .033 |
|  | Time | .41 | .12 | 28.00 | 3.41 | .002 |
|  | Time x Sex | -.08 | .20 | 28.00 | -.42 | .675 |
| MEQ Ineffable | Intercept | 1.86 | .53 | 32.09 | 3.53 | .001 |
|  | MEQ Ineffable | .15 | .14 | 32.09 | 1.08 | .290 |
|  | Time | .24 | .28 | 28.00 | .86 | .399 |
|  | Time x MEQ Ineffable | .04 | .07 | 28.00 | .57 | .576 |
| Intensity | Intercept | 1.28 | .95 | 32.15 | 1.34 | .188 |
|  | Intensity | .01 | .01 | 32.15 | 1.20 | .238 |
|  | Time | .20 | .50 | 28.00 | .41 | .687 |
|  | Time x Intensity | .00 | .01 | 28.00 | .37 | .717 |
| MEQ Mystical | Intercept | 1.56 | .57 | 32.26 | 2.72 | .010 |
|  | MEQ Mystical | .22 | .14 | 32.26 | 1.52 | .138 |
|  | Time | .14 | .31 | 28.00 | .45 | .658 |
|  | Time x MEQ Mystical | .06 | .08 | 28.00 | .85 | .405 |
| BFI Neuroticism | Intercept | 2.57 | 1.05 | 31.38 | 2.46 | .020 |
|  | BFI N | -.04 | .26 | 31.38 | -.17 | .866 |
|  | Time | -.67 | .50 | 28.00 | -1.34 | .190 |
|  | Time x BFI N | .27* | .12 | 28.00 | 2.14 | .041 |
| BFI Openness | Intercept | .63 | .87 | 32.59 | .73 | .471 |
|  | BFI O | .50 | .24 | 32.59 | 2.07 | .046 |
|  | Time | .27 | .48 | 28.00 | .55 | .583 |
|  | Time x BFI O | .03 | .13 | 28.00 | .25 | .805 |
| MEQ Positive mood | Intercept | 1.61 | .57 | 32.23 | 2.81 | .008 |
|  | MEQ Positive mood | .20 | .14 | 32.23 | 1.45 | .156 |
|  | Time | .16 | .30 | 28.00 | .53 | .603 |
|  | Time x MEQ Positive mood | .06 | .07 | 28.00 | .77 | .446 |
| Prev Psychedelic Use | Intercept | 2.23 | .22 | 31.98 | 10.09 | .000 |
|  | PrevPsychUse | .55 | .40 | 31.98 | 1.35 | .186 |
|  | Time | .44 | .11 | 28.00 | 3.86 | .001 |
|  | Time x PrevPsychUse | -.19 | .21 | 28.00 | -.91 | .369 |
| BFAS Intellect | Intercept | 1.39 | .85 | 32.05 | 1.64 | .111 |
|  | BFAS I | .29 | .24 | 32.05 | 1.21 | .236 |
|  | Time | -.14 | .44 | 28.00 | -.32 | .750 |
|  | Time x BFAS I | .15 | .13 | 28.00 | 1.22 | .234 |
| BFAS Openness | Intercept | -.31 | .92 | 33.28 | -.33 | .740 |
|  | BFAS O | .73 | .24 | 33.28 | 2.99 | .005 |
|  | Time | .33 | .54 | 28.00 | .61 | .547 |
|  | Time x BFAS O | .01 | .14 | 28.00 | .10 | .922 |
| BIS-B Impulsivity | Intercept | -.28 | 1.05 | 32.98 | -.26 | .793 |
|  | BIS-B | .97 | .38 | 32.98 | 2.57 | .015 |
|  | Time | .02 | .60 | 28.00 | .03 | .978 |
|  | Time x BIS-B | .13 | .22 | 28.00 | .62 | .543 |
| MEQ Time-space | Intercept | 1.89 | .53 | 32.00 | 3.55 | .001 |
|  | MEQ Time-space | .13 | .13 | 32.00 | 1.02 | .316 |
|  | Time | .11 | .28 | 28.00 | .39 | .696 |
|  | Time x MEQ Time-space | .07 | .07 | 28.00 | 1.06 | .297 |
| Unemployed | Intercept | 2.38 | .22 | 31.92 | 11.01 | .000 |
|  | Unemployed | .07 | .45 | 31.92 | .15 | .884 |
|  | Time | .38 | .11 | 28.00 | 3.43 | .002 |
|  | Time x Unemployed | .01 | .23 | 28.00 | .05 | .957 |
| Openness | | | | | | |
| Age | Intercept | 3.57 | .54 | 34.37 | 6.63 | .000 |
|  | Age | .00 | .01 | 34.37 | -.08 | .937 |
|  | Time | .78 | .35 | 28.00 | 2.25 | .032 |
|  | Time x Age | -.01 | .01 | 28.00 | -1.63 | .114 |
| BFI Agreeableness | Intercept | 4.50 | .60 | 35.71 | 7.56 | .000 |
|  | BFI A | -.30 | .18 | 35.71 | -1.67 | .104 |
|  | Time | .42 | .42 | 28.00 | 1.00 | .327 |
|  | Time x BFI A | -.06 | .13 | 28.00 | -.45 | .656 |
| BFI Conscientiousness | Intercept | 4.18 | .60 | 35.23 | 6.92 | .000 |
|  | BFI C | -.21 | .19 | 35.23 | -1.09 | .281 |
|  | Time | .48 | .41 | 28.00 | 1.18 | .250 |
|  | Time x BFI C | -.08 | .13 | 28.00 | -.62 | .538 |
| Emotional Breakthrough | Intercept | 3.11 | .36 | 35.30 | 8.58 | .000 |
|  | EBI | .01 | .00 | 35.30 | 1.25 | .218 |
|  | Time | .21 | .25 | 28.00 | .86 | .397 |
|  | Time x EBI | .00 | .00 | 28.00 | .09 | .932 |
| Education level | Intercept | 3.62 | .51 | 34.82 | 7.16 | .000 |
|  | EduLevel | -.02 | .13 | 34.82 | -.18 | .860 |
|  | Time | .09 | .34 | 28.00 | .28 | .783 |
|  | Time x EduLevel | .04 | .09 | 28.00 | .44 | .667 |
| Emotional insight | Intercept | 2.95 | .53 | 35.25 | 5.59 | .000 |
|  | Emo Insight | .01 | .01 | 35.25 | 1.13 | .264 |
|  | Time | .13 | .36 | 28.00 | .36 | .721 |
|  | Time x Emo Insight | .00 | .00 | 28.00 | .30 | .769 |
| BFI Extraversion | Intercept | 2.88 | .35 | 35.50 | 8.22 | .000 |
|  | BFI E | .27 | .14 | 35.50 | 2.02 | .051 |
|  | Time | .42 | .24 | 28.00 | 1.72 | .096 |
|  | Time x BFI E | -.08 | .09 | 28.00 | -.82 | .417 |
| Sex | Intercept | 3.43 | .17 | 35.19 | 19.92 | .000 |
|  | Sex | .29 | .28 | 35.19 | 1.03 | .311 |
|  | Time | .20 | .12 | 28.00 | 1.71 | .098 |
|  | Time x Sex | .09 | .19 | 28.00 | .47 | .641 |
| MEQ Ineffable | Intercept | 2.98 | .38 | 35.61 | 7.81 | .000 |
|  | MEQ Ineffable | .15 | .10 | 35.61 | 1.55 | .129 |
|  | Time | .09 | .27 | 28.00 | .32 | .750 |
|  | Time x MEQ Ineffable | .04 | .07 | 28.00 | .59 | .558 |
| Intensity | Intercept | 3.88 | .72 | 34.76 | 5.36 | .000 |
|  | Intensity | .00 | .01 | 34.76 | -.48 | .632 |
|  | Time | -.10 | .48 | 28.00 | -.22 | .829 |
|  | Time x Intensity | .00 | .01 | 28.00 | .72 | .478 |
| MEQ Mystical | Intercept | 2.85 | .42 | 35.74 | 6.72 | .000 |
|  | MEQ Mystical | .18 | .11 | 35.74 | 1.70 | .097 |
|  | Time | .12 | .30 | 28.00 | .40 | .692 |
|  | Time x MEQ Mystical | .03 | .07 | 28.00 | .40 | .690 |
| BFI Neuroticism | Intercept | 2.66 | .76 | 35.28 | 3.52 | .001 |
|  | BFI N | .22 | .19 | 35.28 | 1.17 | .252 |
|  | Time | .00 | .52 | 28.00 | .00 | .997 |
|  | Time x BFI N | .06 | .13 | 28.00 | .46 | .648 |
| BFI Openness | Intercept | .00 | .31 | 56.00 | .00 | 1.000 |
|  | BFI O | 1.00 | .09 | 56.00 | 11.51 | .000 |
|  | Time | .91 | .44 | 56.00 | 2.06 | .044 |
|  | Time x BFI O | -.19 | .12 | 56.00 | -1.56 | .124 |
| MEQ Positive mood | Intercept | 2.83 | .42 | 35.76 | 6.72 | .000 |
|  | MEQ Positive mood | .18 | .10 | 35.76 | 1.77 | .085 |
|  | Time | .19 | .30 | 28.00 | .63 | .535 |
|  | Time x MEQ Positive mood | .01 | .07 | 28.00 | .17 | .867 |
| Prev Psychedelic Use | Intercept | 3.42 | .16 | 35.17 | 20.86 | .000 |
|  | PrevPsychUse | .38 | .30 | 35.17 | 1.27 | .211 |
|  | Time | .26 | .11 | 28.00 | 2.31 | .028 |
|  | Time x PrevPsychUse | -.08 | .20 | 28.00 | -.39 | .699 |
| BFAS Intellect | Intercept | 2.19 | .61 | 36.12 | 3.61 | .001 |
|  | BFAS I | .39 | .17 | 36.12 | 2.25 | .030 |
|  | Time | .34 | .44 | 28.00 | .77 | .449 |
|  | Time x BFAS I | -.03 | .13 | 28.00 | -.24 | .812 |
| BFAS Openness | Intercept | .19 | .52 | 44.15 | .36 | .720 |
|  | BFAS O | .90 | .14 | 44.15 | 6.55 | .000 |
|  | Time | .82 | .51 | 28.00 | 1.62 | .117 |
|  | Time x BFAS O | -.16 | .13 | 28.00 | -1.18 | .248 |
| BIS-B Impulsivity | Intercept | 2.66 | .86 | 35.19 | 3.10 | .004 |
|  | BIS-B | .32 | .31 | 35.19 | 1.03 | .311 |
|  | Time | .01 | .58 | 28.00 | .01 | .989 |
|  | Time x BIS-B | .08 | .21 | 28.00 | .39 | .699 |
| MEQ Time-space | Intercept | 2.99 | .39 | 35.54 | 7.69 | .000 |
|  | MEQ Time-space | .14 | .10 | 35.54 | 1.50 | .142 |
|  | Time | .15 | .27 | 28.00 | .54 | .595 |
|  | Time x MEQ Time-space | .02 | .07 | 28.00 | .35 | .730 |
| Unemployed | Intercept | 3.47 | .16 | 34.63 | 21.77 | .000 |
|  | Unemployed | .29 | .33 | 34.63 | .89 | .382 |
|  | Time | .29 | .10 | 28.00 | 2.80 | .009 |
|  | Time x Unemployed | -.25 | .22 | 28.00 | -1.15 | .259 |
| BFAS Openness | | | | | | |
| Age | Intercept | 3.94 | .51 | 38.27 | 7.75 | .000 |
|  | Age | -.01 | .01 | 38.27 | -.44 | .661 |
|  | Time | 1.00 | .41 | 28.00 | 2.46 | .020 |
|  | Time x Age | -.02 | .01 | 28.00 | -1.87 | .072 |
| BFI Agreeableness | Intercept | 4.64 | .59 | 39.52 | 7.90 | .000 |
|  | BFI A | -.29 | .18 | 39.52 | -1.61 | .115 |
|  | Time | -.02 | .49 | 28.00 | -.04 | .970 |
|  | Time x BFI A | .09 | .15 | 28.00 | .59 | .559 |
| BFI Conscientiousness | Intercept | 3.88 | .60 | 38.87 | 6.47 | .000 |
|  | BFI C | -.05 | .19 | 38.87 | -.27 | .786 |
|  | Time | .20 | .49 | 28.00 | .41 | .682 |
|  | Time x BFI C | .02 | .15 | 28.00 | .13 | .896 |
| Emotional Breakthrough | Intercept | 3.28 | .35 | 39.49 | 9.35 | .000 |
|  | EBI | .01 | .00 | 39.49 | 1.37 | .180 |
|  | Time | .35 | .29 | 28.00 | 1.18 | .249 |
|  | Time x EBI | .00 | .00 | 28.00 | -.29 | .771 |
| Education level | Intercept | 3.88 | .49 | 38.62 | 7.98 | .000 |
|  | EduLevel | -.04 | .13 | 38.62 | -.35 | .728 |
|  | Time | -.05 | .39 | 28.00 | -.12 | .908 |
|  | Time x EduLevel | .08 | .10 | 28.00 | .83 | .416 |
| Emotional insight | Intercept | 3.09 | .51 | 39.17 | 6.02 | .000 |
|  | Emo Insight | .01 | .01 | 39.17 | 1.27 | .210 |
|  | Time | .51 | .43 | 28.00 | 1.21 | .238 |
|  | Time x Emo Insight | .00 | .00 | 28.00 | -.60 | .553 |
| BFI Extraversion | Intercept | 2.90 | .33 | 39.00 | 8.73 | .000 |
|  | BFI E | .34 | .13 | 39.00 | 2.67 | .011 |
|  | Time | .76 | .27 | 28.00 | 2.76 | .010 |
|  | Time x BFI E | -.20 | .11 | 28.00 | -1.93 | .063 |
| Sex | Intercept | 3.58 | .16 | 39.89 | 22.46 | .000 |
|  | Sex | .37 | .26 | 39.89 | 1.41 | .168 |
|  | Time | .17 | .14 | 28.00 | 1.27 | .213 |
|  | Time x Sex | .25 | .23 | 28.00 | 1.13 | .269 |
| MEQ Ineffable | Intercept | 3.03 | .37 | 39.60 | 8.19 | .000 |
|  | MEQ Ineffable | .19 | .10 | 39.60 | 1.98 | .054 |
|  | Time | .54 | .31 | 28.00 | 1.73 | .094 |
|  | Time x MEQ Ineffable | -.08 | .08 | 28.00 | -.94 | .355 |
| Intensity | Intercept | 3.58 | .70 | 38.58 | 5.14 | .000 |
|  | Intensity | .00 | .01 | 38.58 | .20 | .842 |
|  | Time | .76 | .56 | 28.00 | 1.35 | .186 |
|  | Time x Intensity | -.01 | .01 | 28.00 | -.90 | .376 |
| MEQ Mystical | Intercept | 2.86 | .41 | 39.55 | 6.97 | .000 |
|  | MEQ Mystical | .23 | .10 | 39.55 | 2.21 | .033 |
|  | Time | .66 | .35 | 28.00 | 1.91 | .066 |
|  | Time x MEQ Mystical | -.10 | .09 | 28.00 | -1.20 | .239 |
| BFI Neuroticism | Intercept | 3.02 | .74 | 39.35 | 4.11 | .000 |
|  | BFI N | .18 | .18 | 39.35 | .96 | .342 |
|  | Time | .00 | .62 | 28.00 | .00 | .998 |
|  | Time x BFI N | .07 | .15 | 28.00 | .44 | .665 |
| BFI Openness | Intercept | .99 | .48 | 48.21 | 2.04 | .047 |
|  | BFI O | .77 | .13 | 48.21 | 5.75 | .000 |
|  | Time | 1.00 | .53 | 28.00 | 1.89 | .070 |
|  | Time x BFI O | -.21 | .15 | 28.00 | -1.41 | .169 |
| MEQ Positive mood | Intercept | 2.79 | .40 | 39.72 | 6.93 | .000 |
|  | MEQ Positive mood | .24 | .10 | 39.72 | 2.45 | .019 |
|  | Time | .69 | .34 | 28.00 | 2.03 | .052 |
|  | Time x MEQ Positive mood | -.11 | .08 | 28.00 | -1.32 | .198 |
| Prev Psychedelic Use | Intercept | 3.58 | .16 | 39.73 | 22.99 | .000 |
|  | PrevPsychUse | .48 | .28 | 39.73 | 1.69 | .099 |
|  | Time | .30 | .13 | 28.00 | 2.27 | .031 |
|  | Time x PrevPsychUse | -.11 | .24 | 28.00 | -.46 | .648 |
| BFAS Intellect | Intercept | 2.78 | .61 | 39.94 | 4.57 | .000 |
|  | BFAS I | .28 | .17 | 39.94 | 1.59 | .119 |
|  | Time | .28 | .52 | 28.00 | .54 | .595 |
|  | Time x BFAS I | .00 | .15 | 28.00 | -.02 | .981 |
| BFAS Openness | Intercept | .00 | .42 | 56.00 | .00 | 1.000 |
|  | BFAS O | 1.00 | .11 | 56.00 | 9.02 | .000 |
|  | Time | 1.21 | .59 | 56.00 | 2.05 | .045 |
|  | Time x BFAS O | -.25 | .16 | 56.00 | -1.62 | .110 |
| BIS-B Impulsivity | Intercept | 3.00 | .84 | 38.92 | 3.57 | .001 |
|  | BIS-B | .26 | .30 | 38.92 | .86 | .392 |
|  | Time | .64 | .69 | 28.00 | .93 | .361 |
|  | Time x BIS-B | -.14 | .25 | 28.00 | -.55 | .587 |
| MEQ Time-space | Intercept | 3.06 | .38 | 39.31 | 8.12 | .000 |
|  | MEQ Time-space | .17 | .09 | 39.31 | 1.85 | .072 |
|  | Time | .58 | .31 | 28.00 | 1.83 | .078 |
|  | Time x MEQ Time-space | -.08 | .08 | 28.00 | -1.05 | .305 |
| Unemployed | Intercept | 3.70 | .15 | 38.65 | 24.02 | .000 |
|  | Unemployed | .07 | .32 | 38.65 | .21 | .835 |
|  | Time | .31 | .13 | 28.00 | 2.50 | .019 |
|  | Time x Unemployed | -.20 | .26 | 28.00 | -.77 | .450 |
| BFAS Intellect | | | | | | |
| Age | Intercept | 4.38 | .50 | 36.91 | 8.71 | .000 |
|  | Age | -.02 | .01 | 36.91 | -1.99 | .054 |
|  | Time | .42 | .38 | 28.00 | 1.12 | .272 |
|  | Time x Age | .00 | .01 | 28.00 | -.55 | .589 |
| BFI Agreeableness | Intercept | 4.75 | .57 | 37.45 | 8.37 | .000 |
|  | BFI A | -.42 | .17 | 37.45 | -2.42 | .020 |
|  | Time | .34 | .44 | 28.00 | .78 | .441 |
|  | Time x BFI A | -.04 | .13 | 28.00 | -.28 | .784 |
| BFI Conscientiousness | Intercept | 2.96 | .62 | 35.36 | 4.79 | .000 |
|  | BFI C | .14 | .19 | 35.36 | .75 | .460 |
|  | Time | .63 | .42 | 28.00 | 1.48 | .149 |
|  | Time x BFI C | -.13 | .13 | 28.00 | -.98 | .335 |
| Emotional Breakthrough | Intercept | 3.80 | .37 | 35.29 | 10.33 | .000 |
|  | EBI | -.01 | .00 | 35.29 | -1.15 | .256 |
|  | Time | -.08 | .25 | 28.00 | -.31 | .759 |
|  | Time x EBI | .00 | .00 | 28.00 | 1.29 | .207 |
| Education level | Intercept | 3.09 | .50 | 35.71 | 6.18 | .000 |
|  | EduLevel | .09 | .13 | 35.71 | .67 | .506 |
|  | Time | .14 | .35 | 28.00 | .39 | .701 |
|  | Time x EduLevel | .02 | .09 | 28.00 | .26 | .797 |
| Emotional insight | Intercept | 3.45 | .54 | 35.41 | 6.38 | .000 |
|  | Emo Insight | .00 | .01 | 35.41 | -.08 | .939 |
|  | Time | -.06 | .37 | 28.00 | -.16 | .871 |
|  | Time x Emo Insight | .00 | .00 | 28.00 | .79 | .436 |
| BFI Extraversion | Intercept | 3.01 | .36 | 35.27 | 8.27 | .000 |
|  | BFI E | .17 | .14 | 35.27 | 1.20 | .238 |
|  | Time | .53 | .25 | 28.00 | 2.15 | .041 |
|  | Time x BFI E | -.13 | .10 | 28.00 | -1.35 | .188 |
| Sex | Intercept | 3.26 | .17 | 35.88 | 19.02 | .000 |
|  | Sex | .40 | .28 | 35.88 | 1.41 | .166 |
|  | Time | .27 | .12 | 28.00 | 2.21 | .036 |
|  | Time x Sex | -.12 | .20 | 28.00 | -.61 | .545 |
| MEQ Ineffable | Intercept | 3.93 | .39 | 34.99 | 9.97 | .000 |
|  | MEQ Ineffable | -.14 | .10 | 34.99 | -1.41 | .167 |
|  | Time | -.22 | .26 | 28.00 | -.83 | .415 |
|  | Time x MEQ Ineffable | .12 | .07 | 28.00 | 1.78 | .085 |
| Intensity | Intercept | 3.71 | .72 | 35.44 | 5.14 | .000 |
|  | Intensity | .00 | .01 | 35.44 | -.42 | .674 |
|  | Time | -.12 | .50 | 28.00 | -.25 | .807 |
|  | Time x Intensity | .00 | .01 | 28.00 | .71 | .485 |
| MEQ Mystical | Intercept | 3.75 | .45 | 35.18 | 8.43 | .000 |
|  | MEQ Mystical | -.09 | .11 | 35.18 | -.81 | .421 |
|  | Time | -.15 | .30 | 28.00 | -.50 | .618 |
|  | Time x MEQ Mystical | .10 | .08 | 28.00 | 1.31 | .201 |
| BFI Neuroticism | Intercept | 2.43 | .75 | 36.16 | 3.25 | .003 |
|  | BFI N | .25 | .19 | 36.16 | 1.33 | .193 |
|  | Time | -.05 | .54 | 28.00 | -.09 | .928 |
|  | Time x BFI N | .07 | .13 | 28.00 | .51 | .612 |
| BFI Openness | Intercept | 1.96 | .64 | 36.75 | 3.04 | .004 |
|  | BFI O | .41 | .18 | 36.75 | 2.30 | .027 |
|  | Time | .50 | .48 | 28.00 | 1.04 | .307 |
|  | Time x BFI O | -.08 | .13 | 28.00 | -.59 | .562 |
| MEQ Positive mood | Intercept | 3.65 | .44 | 35.29 | 8.23 | .000 |
|  | MEQ Positive mood | -.06 | .11 | 35.29 | -.56 | .578 |
|  | Time | -.08 | .30 | 28.00 | -.26 | .794 |
|  | Time x MEQ Positive mood | .08 | .07 | 28.00 | 1.05 | .301 |
| Prev Psychedelic Use | Intercept | 3.42 | .17 | 35.56 | 20.42 | .000 |
|  | PrevPsychUse | -.03 | .31 | 35.56 | -.10 | .922 |
|  | Time | .22 | .12 | 28.00 | 1.88 | .070 |
|  | Time x PrevPsychUse | .01 | .21 | 28.00 | .07 | .947 |
| BFAS Intellect | Intercept | .00 | .30 | 56.00 | .00 | 1.000 |
|  | BFAS I | 1.00 | .09 | 56.00 | 11.62 | .000 |
|  | Time | 1.08 | .42 | 56.00 | 2.54 | .014 |
|  | Time x BFAS I | -.25* | .12 | 56.00 | -2.06 | .044 |
| BFAS Openness | Intercept | 2.16 | .75 | 36.12 | 2.86 | .007 |
|  | BFAS O | .34 | .20 | 36.12 | 1.69 | .099 |
|  | Time | .53 | .54 | 28.00 | .98 | .338 |
|  | Time x BFAS O | -.08 | .14 | 28.00 | -.57 | .572 |
| BIS-B Impulsivity | Intercept | 3.71 | .88 | 35.49 | 4.23 | .000 |
|  | BIS-B | -.11 | .31 | 35.49 | -.35 | .729 |
|  | Time | -.10 | .61 | 28.00 | -.17 | .868 |
|  | Time x BIS-B | .12 | .22 | 28.00 | .54 | .592 |
| MEQ Time-space | Intercept | 3.67 | .40 | 35.22 | 9.08 | .000 |
|  | MEQ Time-space | -.07 | .10 | 35.22 | -.68 | .500 |
|  | Time | -.09 | .28 | 28.00 | -.32 | .752 |
|  | Time x MEQ Time-space | .08 | .07 | 28.00 | 1.20 | .238 |
| Unemployed | Intercept | 3.41 | .16 | 35.40 | 21.41 | .000 |
|  | Unemployed | -.01 | .33 | 35.40 | -.04 | .969 |
|  | Time | .27 | .11 | 28.00 | 2.46 | .021 |
|  | Time x Unemployed | -.20 | .23 | 28.00 | -.87 | .391 |
| Absorption | | | | | | |
| Age | Intercept | 2.62 | .58 | 31.40 | 4.51 | .000 |
|  | Age | -.01 | .01 | 31.40 | -.78 | .440 |
|  | Time | .54 | .28 | 28.00 | 1.93 | .064 |
|  | Time x Age | -.01 | .01 | 28.00 | -.87 | .394 |
| BFI Agreeableness | Intercept | 2.84 | .67 | 31.50 | 4.23 | .000 |
|  | BFI A | -.20 | .20 | 31.50 | -1.00 | .324 |
|  | Time | .34 | .33 | 28.00 | 1.04 | .309 |
|  | Time x BFI A | -.01 | .10 | 28.00 | -.11 | .915 |
| BFI Conscientiousness | Intercept | 2.27 | .68 | 31.31 | 3.37 | .002 |
|  | BFI C | -.03 | .21 | 31.31 | -.14 | .893 |
|  | Time | .08 | .32 | 28.00 | .26 | .797 |
|  | Time x BFI C | .07 | .10 | 28.00 | .71 | .483 |
| Emotional Breakthrough | Intercept | 1.36 | .35 | 32.10 | 3.84 | .001 |
|  | EBI | .01 | .00 | 32.10 | 2.50 | .018 |
|  | Time | .04 | .19 | 28.00 | .21 | .836 |
|  | Time x EBI | .00 | .00 | 28.00 | 1.54 | .134 |
| Education level | Intercept | 1.87 | .54 | 31.26 | 3.46 | .002 |
|  | EduLevel | .08 | .14 | 31.26 | .61 | .549 |
|  | Time | -.03 | .25 | 28.00 | -.12 | .902 |
|  | Time x EduLevel | .09 | .07 | 28.00 | 1.38 | .179 |
| Emotional insight | Intercept | 1.10 | .53 | 31.79 | 2.08 | .045 |
|  | Emo Insight | .01 | .01 | 31.79 | 2.11 | .042 |
|  | Time | -.14 | .27 | 28.00 | -.54 | .594 |
|  | Time x Emo Insight | .01 | .00 | 28.00 | 1.74 | .093 |
| BFI Extraversion | Intercept | 1.75 | .39 | 31.56 | 4.51 | .000 |
|  | BFI E | .18 | .15 | 31.56 | 1.22 | .233 |
|  | Time | .20 | .19 | 28.00 | 1.04 | .309 |
|  | Time x BFI E | .04 | .07 | 28.00 | .61 | .546 |
| Sex | Intercept | 2.16 | .19 | 31.20 | 11.34 | .000 |
|  | Sex | .07 | .31 | 31.20 | .23 | .817 |
|  | Time | .23 | .09 | 28.00 | 2.62 | .014 |
|  | Time x Sex | .20 | .15 | 28.00 | 1.34 | .192 |
| MEQ Ineffable | Intercept | 1.13 | .36 | 32.54 | 3.15 | .004 |
|  | MEQ Ineffable | .29 | .09 | 32.54 | 3.13 | .004 |
|  | Time | -.03 | .20 | 28.00 | -.14 | .892 |
|  | Time x MEQ Ineffable | .09 | .05 | 28.00 | 1.79 | .084 |
| Intensity | Intercept | 1.06 | .76 | 31.65 | 1.39 | .174 |
|  | Intensity | .01 | .01 | 31.65 | 1.52 | .139 |
|  | Time | .35 | .38 | 28.00 | .93 | .360 |
|  | Time x Intensity | .00 | .00 | 28.00 | -.12 | .902 |
| MEQ Mystical | Intercept | .99 | .39 | 32.33 | 2.54 | .016 |
|  | MEQ Mystical | .31 | .10 | 32.33 | 3.23 | .003 |
|  | Time | -.21 | .21 | 28.00 | -.98 | .335 |
|  | Time x MEQ Mystical | .13* | .05 | 28.00 | 2.56 | .016 |
| BFI Neuroticism | Intercept | .87 | .77 | 31.12 | 1.14 | .263 |
|  | BFI N | .33 | .19 | 31.12 | 1.74 | .092 |
|  | Time | -.73 | .35 | 28.00 | -2.08 | .047 |
|  | Time x BFI N | .26** | .09 | 28.00 | 2.99 | .006 |
| BFI Openness | Intercept | .25 | .64 | 32.67 | .40 | .693 |
|  | BFI O | .55 | .18 | 32.67 | 3.08 | .004 |
|  | Time | .03 | .36 | 28.00 | .07 | .944 |
|  | Time x BFI O | .08 | .10 | 28.00 | .80 | .431 |
| MEQ Positive mood | Intercept | .92 | .37 | 32.61 | 2.47 | .019 |
|  | MEQ Positive mood | .33 | .09 | 32.61 | 3.55 | .001 |
|  | Time | -.21 | .21 | 28.00 | -.99 | .330 |
|  | Time x MEQ Positive mood | .13* | .05 | 28.00 | 2.59 | .015 |
| Prev Psychedelic Use | Intercept | 2.09 | .18 | 31.28 | 11.58 | .000 |
|  | PrevPsychUse | .31 | .33 | 31.28 | .95 | .352 |
|  | Time | .36 | .09 | 28.00 | 4.20 | .000 |
|  | Time x PrevPsychUse | -.18 | .16 | 28.00 | -1.14 | .265 |
| BFAS Intellect | Intercept | 1.44 | .70 | 31.52 | 2.06 | .047 |
|  | BFAS I | .22 | .20 | 31.52 | 1.09 | .286 |
|  | Time | .31 | .34 | 28.00 | .91 | .369 |
|  | Time x BFAS I | .00 | .10 | 28.00 | -.02 | .984 |
| BFAS Openness | Intercept | -.44 | .67 | 33.54 | -.65 | .518 |
|  | BFAS O | .71 | .18 | 33.54 | 3.97 | .000 |
|  | Time | .07 | .40 | 28.00 | .17 | .864 |
|  | Time x BFAS O | .06 | .11 | 28.00 | .59 | .561 |
| BIS-B Impulsivity | Intercept | 1.80 | .95 | 31.33 | 1.89 | .068 |
|  | BIS-B | .14 | .34 | 31.33 | .42 | .681 |
|  | Time | -.07 | .45 | 28.00 | -.15 | .883 |
|  | Time x BIS-B | .13 | .16 | 28.00 | .83 | .411 |
| MEQ Time-space | Intercept | 1.19 | .36 | 32.05 | 3.28 | .003 |
|  | MEQ Time-space | .26 | .09 | 32.05 | 2.94 | .006 |
|  | Time | -.16 | .19 | 28.00 | -.87 | .391 |
|  | Time x MEQ Time-space | .12* | .05 | 28.00 | 2.65 | .013 |
| Unemployed | Intercept | 2.13 | .17 | 31.39 | 12.39 | .000 |
|  | Unemployed | .22 | .36 | 31.39 | .63 | .532 |
|  | Time | .28 | .08 | 28.00 | 3.35 | .002 |
|  | Time x Unemployed | .12 | .17 | 28.00 | .72 | .475 |
| Agreeableness | | | | | | |
| Age | Intercept | 2.33 | .52 | 37.62 | 4.45 | .000 |
|  | Age | .02 | .01 | 37.62 | 1.74 | .089 |
|  | Time | 1.11 | .41 | 28.00 | 2.74 | .011 |
|  | Time x Age | -.01 | .01 | 28.00 | -1.63 | .114 |
| BFI Agreeableness | Intercept | .00 | .32 | 56.00 | .00 | 1.000 |
|  | BFI A | 1.00 | .10 | 56.00 | 10.32 | .000 |
|  | Time | 1.47 | .45 | 55.99 | 3.26 | .002 |
|  | Time x BFI A | -.31* | .14 | 55.99 | -2.27 | .027 |
| BFI Conscientiousness | Intercept | 2.94 | .61 | 37.68 | 4.79 | .000 |
|  | BFI C | .09 | .19 | 37.68 | .46 | .649 |
|  | Time | .93 | .48 | 28.00 | 1.95 | .062 |
|  | Time x BFI C | -.15 | .15 | 28.00 | -.98 | .337 |
| Emotional Breakthrough | Intercept | 2.63 | .35 | 39.13 | 7.50 | .000 |
|  | EBI | .01 | .00 | 39.13 | 1.78 | .083 |
|  | Time | .52 | .29 | 28.00 | 1.79 | .085 |
|  | Time x EBI | .00 | .00 | 28.00 | -.17 | .869 |
| Education level | Intercept | 3.64 | .50 | 37.43 | 7.35 | .000 |
|  | EduLevel | -.12 | .13 | 37.43 | -.90 | .372 |
|  | Time | -.03 | .38 | 28.00 | -.09 | .929 |
|  | Time x EduLevel | .14 | .10 | 28.00 | 1.39 | .176 |
| Emotional insight | Intercept | 2.67 | .52 | 38.52 | 5.10 | .000 |
|  | Emo Insight | .01 | .01 | 38.52 | 1.08 | .287 |
|  | Time | .36 | .42 | 28.00 | .85 | .402 |
|  | Time x Emo Insight | .00 | .00 | 28.00 | .28 | .781 |
| BFI Extraversion | Intercept | 3.56 | .36 | 37.62 | 9.88 | .000 |
|  | BFI E | -.15 | .14 | 37.62 | -1.06 | .298 |
|  | Time | .15 | .28 | 28.00 | .54 | .591 |
|  | Time x BFI E | .13 | .11 | 28.00 | 1.24 | .224 |
| Sex | Intercept | 3.36 | .17 | 38.05 | 19.67 | .000 |
|  | Sex | -.40 | .28 | 38.05 | -1.41 | .167 |
|  | Time | .39 | .14 | 28.00 | 2.90 | .007 |
|  | Time x Sex | .22 | .22 | 28.00 | 1.01 | .323 |
| MEQ Ineffable | Intercept | 3.00 | .39 | 38.09 | 7.67 | .000 |
|  | MEQ Ineffable | .06 | .10 | 38.09 | .57 | .575 |
|  | Time | .26 | .31 | 28.00 | .85 | .402 |
|  | Time x MEQ Ineffable | .06 | .08 | 28.00 | .72 | .476 |
| Intensity | Intercept | 2.89 | .70 | 37.87 | 4.11 | .000 |
|  | Intensity | .00 | .01 | 37.87 | .46 | .647 |
|  | Time | -.16 | .55 | 28.00 | -.28 | .781 |
|  | Time x Intensity | .01 | .01 | 28.00 | 1.16 | .255 |
| MEQ Mystical | Intercept | 3.17 | .44 | 37.85 | 7.18 | .000 |
|  | MEQ Mystical | .01 | .11 | 37.85 | .10 | .925 |
|  | Time | .22 | .35 | 28.00 | .63 | .531 |
|  | Time x MEQ Mystical | .07 | .09 | 28.00 | .77 | .445 |
| BFI Neuroticism | Intercept | 4.80 | .73 | 38.88 | 6.59 | .000 |
|  | BFI N | -.40 | .18 | 38.88 | -2.22 | .032 |
|  | Time | -.13 | .60 | 28.00 | -.22 | .830 |
|  | Time x BFI N | .15 | .15 | 28.00 | 1.03 | .314 |
| BFI Openness | Intercept | 4.29 | .66 | 38.47 | 6.46 | .000 |
|  | BFI O | -.31 | .18 | 38.47 | -1.66 | .105 |
|  | Time | .06 | .54 | 28.00 | .11 | .912 |
|  | Time x BFI O | .12 | .15 | 28.00 | .79 | .436 |
| MEQ Positive mood | Intercept | 3.12 | .44 | 37.97 | 7.13 | .000 |
|  | MEQ Positive mood | .02 | .11 | 37.97 | .21 | .832 |
|  | Time | .31 | .35 | 28.00 | .91 | .371 |
|  | Time x MEQ Positive mood | .04 | .08 | 28.00 | .49 | .628 |
| Prev Psychedelic Use | Intercept | 3.16 | .16 | 37.29 | 19.17 | .000 |
|  | PrevPsychUse | .17 | .30 | 37.29 | .58 | .565 |
|  | Time | .58 | .13 | 28.00 | 4.58 | .000 |
|  | Time x PrevPsychUse | -.34 | .23 | 28.00 | -1.49 | .148 |
| BFAS Intellect | Intercept | 4.59 | .61 | 39.08 | 7.53 | .000 |
|  | BFAS I | -.40 | .17 | 39.08 | -2.31 | .026 |
|  | Time | -.01 | .50 | 28.00 | -.01 | .991 |
|  | Time x BFAS I | .14 | .14 | 28.00 | .98 | .338 |
| BFAS Openness | Intercept | 4.47 | .74 | 38.98 | 6.03 | .000 |
|  | BFAS O | -.34 | .20 | 38.98 | -1.72 | .093 |
|  | Time | .30 | .61 | 28.00 | .50 | .623 |
|  | Time x BFAS O | .05 | .16 | 28.00 | .28 | .779 |
| BIS-B Impulsivity | Intercept | 4.60 | .85 | 36.95 | 5.43 | .000 |
|  | BIS-B | -.50 | .30 | 36.95 | -1.66 | .105 |
|  | Time | -.85 | .64 | 28.00 | -1.33 | .193 |
|  | Time x BIS-B | .48* | .23 | 28.00 | 2.10 | .044 |
| MEQ Time-space | Intercept | 3.01 | .40 | 38.10 | 7.58 | .000 |
|  | MEQ Time-space | .05 | .10 | 38.10 | .54 | .592 |
|  | Time | .30 | .31 | 28.00 | .96 | .343 |
|  | Time x MEQ Time-space | .04 | .08 | 28.00 | .58 | .568 |
| Unemployed | Intercept | 3.16 | .16 | 37.72 | 20.04 | .000 |
|  | Unemployed | .22 | .33 | 37.72 | .68 | .501 |
|  | Time | .53 | .12 | 28.00 | 4.32 | .000 |
|  | Time x Unemployed | -.25 | .25 | 28.00 | -.97 | .342 |
| Conscientiousness | | | | | | |
| Age | Intercept | 3.00 | .51 | 40.29 | 5.88 | .000 |
|  | Age | .00 | .01 | 40.29 | .21 | .835 |
|  | Time | .17 | .44 | 28.00 | .39 | .702 |
|  | Time x Age | .00 | .01 | 28.00 | .30 | .766 |
| BFI Agreeableness | Intercept | 2.83 | .59 | 40.37 | 4.81 | .000 |
|  | BFI A | .08 | .18 | 40.37 | .47 | .638 |
|  | Time | .30 | .51 | 28.00 | .59 | .557 |
|  | Time x BFI A | .00 | .16 | 28.00 | -.01 | .993 |
| BFI Conscientiousness | Intercept | .00 | .31 | 56.00 | .00 | 1.000 |
|  | BFI C | 1.00 | .10 | 56.00 | 10.24 | .000 |
|  | Time | 1.58 | .44 | 56.00 | 3.60 | .001 |
|  | Time x BFI C | -.41*** | .14 | 56.00 | -2.99 | .004 |
| Emotional Breakthrough | Intercept | 2.96 | .35 | 40.34 | 8.47 | .000 |
|  | EBI | .00 | .00 | 40.34 | .45 | .657 |
|  | Time | .32 | .30 | 28.00 | 1.07 | .295 |
|  | Time x EBI | .00 | .00 | 28.00 | -.08 | .933 |
| Education level | Intercept | 3.11 | .47 | 40.24 | 6.56 | .000 |
|  | EduLevel | .00 | .12 | 40.24 | -.02 | .987 |
|  | Time | .20 | .41 | 28.00 | .49 | .629 |
|  | Time x EduLevel | .03 | .11 | 28.00 | .25 | .803 |
| Emotional insight | Intercept | 2.71 | .50 | 40.66 | 5.42 | .000 |
|  | Emo Insight | .00 | .01 | 40.66 | .81 | .423 |
|  | Time | .16 | .44 | 28.00 | .35 | .727 |
|  | Time x Emo Insight | .00 | .01 | 28.00 | .34 | .736 |
| BFI Extraversion | Intercept | 3.11 | .35 | 40.12 | 9.01 | .000 |
|  | BFI E | .00 | .13 | 40.12 | -.02 | .984 |
|  | Time | .11 | .30 | 28.00 | .37 | .715 |
|  | Time x BFI E | .08 | .11 | 28.00 | .69 | .495 |
| Sex | Intercept | 3.06 | .16 | 40.27 | 18.76 | .000 |
|  | Sex | .11 | .27 | 40.27 | .40 | .693 |
|  | Time | .23 | .14 | 28.00 | 1.61 | .118 |
|  | Time x Sex | .20 | .23 | 28.00 | .84 | .408 |
| MEQ Ineffable | Intercept | 3.40 | .37 | 39.39 | 9.09 | .000 |
|  | MEQ Ineffable | -.08 | .10 | 39.39 | -.85 | .402 |
|  | Time | -.16 | .31 | 28.00 | -.50 | .618 |
|  | Time x MEQ Ineffable | .13 | .08 | 28.00 | 1.56 | .130 |
| Intensity | Intercept | 3.02 | .68 | 40.17 | 4.47 | .000 |
|  | Intensity | .00 | .01 | 40.17 | .12 | .905 |
|  | Time | -.08 | .58 | 28.00 | -.14 | .891 |
|  | Time x Intensity | .00 | .01 | 28.00 | .66 | .512 |
| MEQ Mystical | Intercept | 3.15 | .42 | 39.67 | 7.56 | .000 |
|  | MEQ Mystical | -.01 | .10 | 39.67 | -.13 | .901 |
|  | Time | -.16 | .35 | 28.00 | -.44 | .664 |
|  | Time x MEQ Mystical | .12 | .09 | 28.00 | 1.36 | .186 |
| BFI Neuroticism | Intercept | 3.33 | .73 | 40.25 | 4.54 | .000 |
|  | BFI N | -.06 | .18 | 40.25 | -.31 | .760 |
|  | Time | .11 | .63 | 28.00 | .18 | .860 |
|  | Time x BFI N | .05 | .16 | 28.00 | .30 | .766 |
| BFI Openness | Intercept | 3.82 | .64 | 40.45 | 5.94 | .000 |
|  | BFI O | -.20 | .18 | 40.45 | -1.13 | .264 |
|  | Time | -.06 | .56 | 28.00 | -.10 | .920 |
|  | Time x BFI O | .10 | .16 | 28.00 | .65 | .520 |
| MEQ Positive mood | Intercept | 3.04 | .41 | 39.97 | 7.37 | .000 |
|  | MEQ Positive mood | .02 | .10 | 39.97 | .16 | .871 |
|  | Time | -.08 | .35 | 28.00 | -.23 | .817 |
|  | Time x MEQ Positive mood | .10 | .09 | 28.00 | 1.14 | .264 |
| Prev Psychedelic Use | Intercept | 3.10 | .16 | 39.91 | 19.78 | .000 |
|  | PrevPsychUse | .03 | .29 | 39.91 | .10 | .922 |
|  | Time | .38 | .13 | 28.00 | 2.81 | .009 |
|  | Time x PrevPsychUse | -.25 | .24 | 28.00 | -1.03 | .310 |
| BFAS Intellect | Intercept | 2.64 | .61 | 40.31 | 4.32 | .000 |
|  | BFAS I | .13 | .18 | 40.31 | .77 | .447 |
|  | Time | .57 | .53 | 28.00 | 1.07 | .296 |
|  | Time x BFAS I | -.08 | .15 | 28.00 | -.51 | .612 |
| BFAS Openness | Intercept | 3.32 | .74 | 40.24 | 4.51 | .000 |
|  | BFAS O | -.06 | .19 | 40.24 | -.30 | .767 |
|  | Time | .10 | .64 | 28.00 | .16 | .871 |
|  | Time x BFAS O | .05 | .17 | 28.00 | .31 | .757 |
| BIS-B Impulsivity | Intercept | 4.90 | .78 | 40.39 | 6.25 | .000 |
|  | BIS-B | -.65 | .28 | 40.39 | -2.32 | .025 |
|  | Time | -.82 | .68 | 28.00 | -1.21 | .238 |
|  | Time x BIS-B | .41 | .24 | 28.00 | 1.67 | .106 |
| MEQ Time-space | Intercept | 3.19 | .38 | 39.71 | 8.43 | .000 |
|  | MEQ Time-space | -.02 | .09 | 39.71 | -.25 | .800 |
|  | Time | -.08 | .32 | 28.00 | -.24 | .812 |
|  | Time x MEQ Time-space | .10 | .08 | 28.00 | 1.25 | .221 |
| Unemployed | Intercept | 3.15 | .15 | 40.52 | 21.20 | .000 |
|  | Unemployed | -.20 | .31 | 40.52 | -.64 | .525 |
|  | Time | .32 | .13 | 28.00 | 2.49 | .019 |
|  | Time x Unemployed | -.10 | .27 | 28.00 | -.38 | .709 |
| BIS-B Impulsivity | | | | | | |
| Age | Intercept | 2.93 | .27 | 37.65 | 10.90 | .000 |
|  | Age | .00 | .01 | 37.65 | .37 | .714 |
|  | Time | -.26 | .21 | 28.00 | -1.26 | .217 |
|  | Time x Age | .00 | .00 | 28.00 | -.70 | .490 |
| BFI Agreeableness | Intercept | 3.62 | .30 | 38.41 | 12.26 | .000 |
|  | BFI A | -.19 | .09 | 38.41 | -2.10 | .043 |
|  | Time | -.66 | .24 | 28.00 | -2.80 | .009 |
|  | Time x BFI A | .08 | .07 | 28.00 | 1.12 | .270 |
| BFI Conscientiousness | Intercept | 3.72 | .27 | 40.46 | 13.53 | .000 |
|  | BFI C | -.23 | .09 | 40.46 | -2.61 | .013 |
|  | Time | -.41 | .24 | 28.00 | -1.70 | .100 |
|  | Time x BFI C | .00 | .08 | 28.00 | .02 | .986 |
| Emotional Breakthrough | Intercept | 3.13 | .18 | 37.98 | 17.16 | .000 |
|  | EBI | .00 | .00 | 37.98 | -.64 | .526 |
|  | Time | -.39 | .14 | 28.00 | -2.68 | .012 |
|  | Time x EBI | .00 | .00 | 28.00 | -.14 | .887 |
| Education level | Intercept | 2.87 | .25 | 37.49 | 11.55 | .000 |
|  | EduLevel | .04 | .06 | 37.49 | .65 | .519 |
|  | Time | -.21 | .19 | 28.00 | -1.12 | .273 |
|  | Time x EduLevel | -.05 | .05 | 28.00 | -1.03 | .310 |
| Emotional insight | Intercept | 3.24 | .26 | 38.11 | 12.47 | .000 |
|  | Emo Insight | .00 | .00 | 38.11 | -.87 | .391 |
|  | Time | -.22 | .21 | 28.00 | -1.09 | .286 |
|  | Time x Emo Insight | .00 | .00 | 28.00 | -.90 | .374 |
| BFI Extraversion | Intercept | 2.67 | .17 | 38.51 | 15.51 | .000 |
|  | BFI E | .15 | .07 | 38.51 | 2.23 | .031 |
|  | Time | -.25 | .14 | 28.00 | -1.82 | .079 |
|  | Time x BFI E | -.06 | .05 | 28.00 | -1.18 | .248 |
| Sex | Intercept | 3.02 | .09 | 37.80 | 34.76 | .000 |
|  | Sex | .00 | .14 | 37.80 | .02 | .983 |
|  | Time | -.40 | .07 | 28.00 | -5.90 | .000 |
|  | Time x Sex | -.01 | .11 | 28.00 | -.07 | .945 |
| MEQ Ineffable | Intercept | 2.90 | .20 | 37.41 | 14.72 | .000 |
|  | MEQ Ineffable | .03 | .05 | 37.41 | .63 | .530 |
|  | Time | -.24 | .15 | 28.00 | -1.60 | .121 |
|  | Time x MEQ Ineffable | -.04 | .04 | 28.00 | -1.14 | .264 |
| Intensity | Intercept | 2.93 | .36 | 37.49 | 8.24 | .000 |
|  | Intensity | .00 | .00 | 37.49 | .25 | .806 |
|  | Time | -.14 | .27 | 28.00 | -.49 | .626 |
|  | Time x Intensity | .00 | .00 | 28.00 | -1.00 | .327 |
| MEQ Mystical | Intercept | 2.88 | .22 | 36.90 | 13.11 | .000 |
|  | MEQ Mystical | .04 | .05 | 36.90 | .68 | .500 |
|  | Time | -.13 | .16 | 28.00 | -.80 | .428 |
|  | Time x MEQ Mystical | -.07 | .04 | 28.00 | -1.74 | .093 |
| BFI Neuroticism | Intercept | 2.39 | .38 | 36.96 | 6.38 | .000 |
|  | BFI N | .16 | .09 | 36.96 | 1.70 | .098 |
|  | Time | .15 | .28 | 28.00 | .52 | .605 |
|  | Time x BFI N | -.14 | .07 | 28.00 | -1.99 | .056 |
| BFI Openness | Intercept | 2.78 | .34 | 37.79 | 8.17 | .000 |
|  | BFI O | .07 | .09 | 37.79 | .73 | .472 |
|  | Time | -.26 | .27 | 28.00 | -.96 | .345 |
|  | Time x BFI O | -.04 | .07 | 28.00 | -.57 | .573 |
| MEQ Positive mood | Intercept | 3.02 | .22 | 37.30 | 13.89 | .000 |
|  | MEQ Positive mood | .00 | .05 | 37.30 | .02 | .982 |
|  | Time | -.19 | .17 | 28.00 | -1.12 | .272 |
|  | Time x MEQ Positive mood | -.06 | .04 | 28.00 | -1.39 | .177 |
| Prev Psychedelic Use | Intercept | 3.04 | .08 | 37.19 | 37.00 | .000 |
|  | PrevPsychUse | -.07 | .15 | 37.19 | -.46 | .646 |
|  | Time | -.45 | .06 | 28.00 | -7.24 | .000 |
|  | Time x PrevPsychUse | .16 | .11 | 28.00 | 1.41 | .170 |
| BFAS Intellect | Intercept | 3.11 | .32 | 37.53 | 9.77 | .000 |
|  | BFAS I | -.03 | .09 | 37.53 | -.30 | .766 |
|  | Time | -.10 | .25 | 28.00 | -.40 | .689 |
|  | Time x BFAS I | -.09 | .07 | 28.00 | -1.27 | .216 |
| BFAS Openness | Intercept | 2.80 | .39 | 37.72 | 7.26 | .000 |
|  | BFAS O | .06 | .10 | 37.72 | .58 | .565 |
|  | Time | -.22 | .30 | 28.00 | -.74 | .466 |
|  | Time x BFAS O | -.05 | .08 | 28.00 | -.61 | .544 |
| BIS-B Impulsivity | Intercept | .69 | .22 | 55.17 | 3.09 | .003 |
|  | BIS-B | .85 | .08 | 55.17 | 10.66 | .000 |
|  | Time | .48 | .29 | 28.00 | 1.63 | .115 |
|  | Time x BIS-B | -.32** | .11 | 28.00 | -3.04 | .005 |
| BIS-B Impulsivity | Intercept | .39 | .30 | 43.00 | 1.31 | .196 |
| (without upper 20% | BIS-B | .97 | .11 | 43.00 | 8.51 | .000 |
| of patients) | Time | .62 | .38 | 22.00 | 1.62 | .120 |
|  | Time x BIS-B | -.38* | .15 | 22.00 | -2.61 | .016 |
| MEQ Time-space | Intercept | 3.04 | .20 | 37.45 | 15.36 | .000 |
|  | MEQ Time-space | -.01 | .05 | 37.45 | -.12 | .904 |
|  | Time | -.23 | .15 | 28.00 | -1.48 | .150 |
|  | Time x MEQ Time-space | -.05 | .04 | 28.00 | -1.25 | .223 |
| Unemployed | Intercept | 2.99 | .08 | 38.03 | 38.38 | .000 |
|  | Unemployed | .11 | .16 | 38.03 | .70 | .490 |
|  | Time | -.41 | .06 | 28.00 | -6.70 | .000 |
|  | Time x Unemployed | .04 | .13 | 28.00 | .30 | .768 |
| *Note.* B = unstandardized coefficient; df = degrees of freedom. BFI = Big Five Inventory; BFAS = Big Five Aspects Scale; BFI Neuroticism = mean-score without Item 4; Absorption = Modified-Tellegen Absorption Scale Absorption without MODTAS Item 5; BIS-B Impulsivity = Barrett Impulsivity Inventory-Brief mean-score. *p<.05, ***p*<.01, ****p*<.005. | | | | | | |

Figures

Supplementary Figure 1. Information regarding usage of medication, psychedelics, and therapy between Week 6 and Month 6 by condition.

**References**

Benjamini, Y., & Hochberg, Y. (1995). Controlling the false discovery rate: a practical and powerful approach to multiple testing. Journal of the Royal statistical society: series B (Methodological), 57(1), 289-300.

Byrne, B. M. (2013). Structural equation modeling with Mplus: Basic concepts, applications, *and programming*. routledge.

Carhart-Harris, R., Giribaldi, B., Watts, R., Baker-Jones, M., Murphy-Beiner, A., Murphy, R., Martell, J., Blemings, A., Erritzoe, D., & Nutt, D. J. (2021). Trial of psilocybin versus escitalopram for depression. *New England Journal of Medicine*, *384*(15), 1402-1411.

Hair, J. F., Black, W. C., Babin, B. J., & Anderson, R. E. (2019). Multivariate data analysis (8. Baskı). *Eight Edition, Cengage: Learning EMEA*.

Rush, A. J., Trivedi, M. H., Ibrahim, H. M., Carmody, T. J., Arnow, B., Klein, D. N., Markowitz, J. C., Ninan, P. T., Kornstein, S., & Manber, R. (2003). The 16-Item Quick Inventory of Depressive Symptomatology (QIDS), clinician rating (QIDS-C), and self-report (QIDS-SR): a psychometric evaluation in patients with chronic major depression. *Biological psychiatry*, *54*(5), 573-583.

Tang, T. Z., DeRubeis, R. J., Hollon, S. D., Amsterdam, J., Shelton, R., & Schalet, B. (2009). Personality change during depression treatment: a placebo-controlled trial. *Archives of general psychiatry*, *66*(12), 1322-1330.
