# Supplementary figures and images for: Personality change in a trial of psilocybin therapy v. escitalopram treatment for depression
Source: Psychol Med. Author manuscript; Available in PMC 2024 Feb 16. (PMC10692311; doi:10.1017/S0033291723001514)

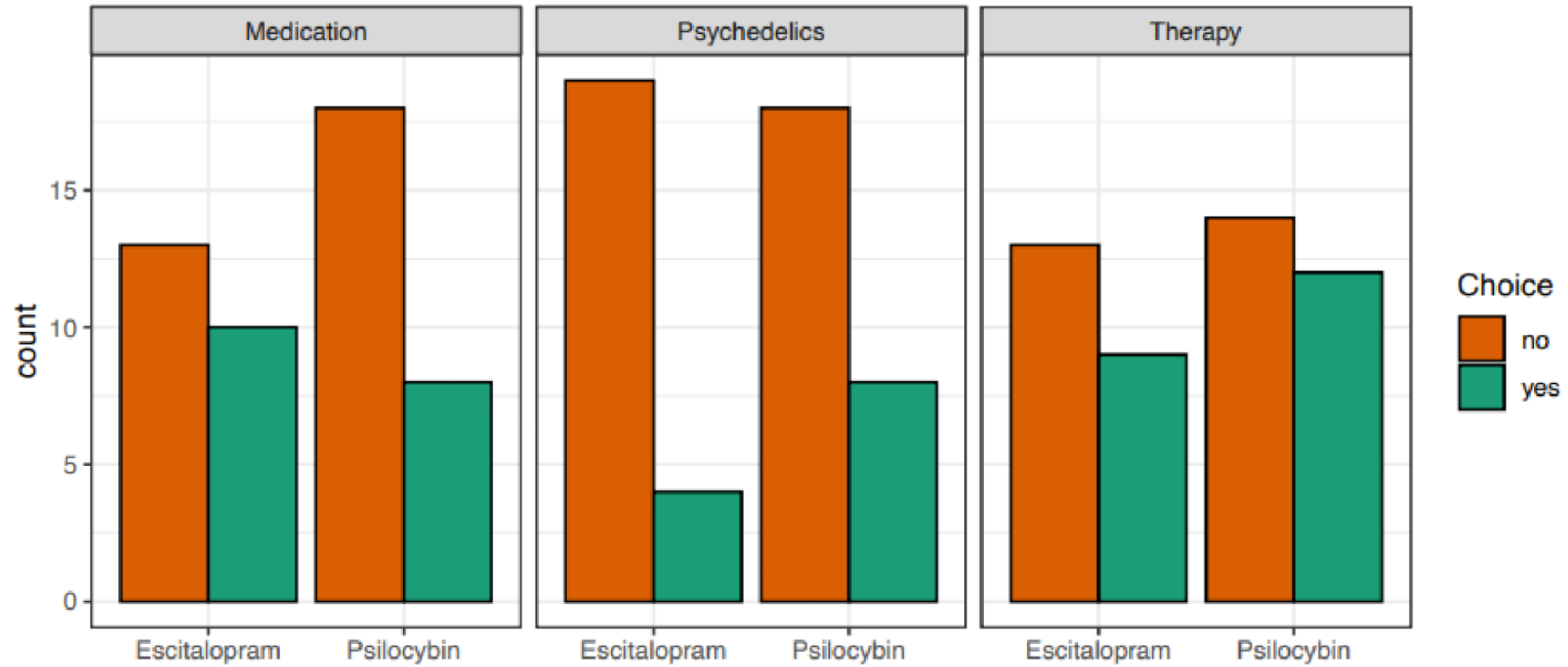

Supplement: Supplementary Figure 1 [file NIHMS1932967-supplement-Supplementary_Figure_1.pdf]
